# Binding Site Turnover Produces Pervasive Quantitative Changes in Transcription Factor Binding between Closely Related *Drosophila* Species

**DOI:** 10.1371/journal.pbio.1000343

**Published:** 2010-03-23

**Authors:** Robert K. Bradley, Xiao-Yong Li, Cole Trapnell, Stuart Davidson, Lior Pachter, Hou Cheng Chu, Leath A. Tonkin, Mark D. Biggin, Michael B. Eisen

**Affiliations:** 1 Department of Mathematics, University of California Berkeley, Berkeley, California, United States of America; 2 Department of Molecular and Cell Biology, University of California Berkeley, Berkeley, California, United States of America; 3 Howard Hughes Medical Institute, University of California Berkeley, Berkeley, California, United States of America; 4 Genomics Division, Ernest Orlando Lawrence Berkeley National Lab, Berkeley, California, United States of America; 5 Center for Bioinformatics and Computational Biology, University of Maryland, College Park, Maryland, United States of America; 6 California Institute for Quantitative Biosciences, University of California Berkeley, Berkeley, California, United States of America; 7 Vincent J. Coates Genome Sequencing Laboratory, University of California Berkeley, Berkeley, California, United States of America; Duke University, United States of America

## Abstract

Changes in gene expression play an important role in evolution, yet the molecular mechanisms underlying regulatory evolution are poorly understood. Here we compare genome-wide binding of the six transcription factors that initiate segmentation along the anterior-posterior axis in embryos of two closely related species: *Drosophila melanogaster* and *Drosophila yakuba*. Where we observe binding by a factor in one species, we almost always observe binding by that factor to the orthologous sequence in the other species. Levels of binding, however, vary considerably. The magnitude and direction of the interspecies differences in binding levels of all six factors are strongly correlated, suggesting a role for chromatin or other factor-independent forces in mediating the divergence of transcription factor binding. Nonetheless, factor-specific quantitative variation in binding is common, and we show that it is driven to a large extent by the gain and loss of cognate recognition sequences for the given factor. We find only a weak correlation between binding variation and regulatory function. These data provide the first genome-wide picture of how modest levels of sequence divergence between highly morphologically similar species affect a system of coordinately acting transcription factors during animal development, and highlight the dominant role of quantitative variation in transcription factor binding over short evolutionary distances.

## Abstract 2

Genome-wide comparison of transcription factor binding between related *Drosophila* species highlights how sequence changes affect the biochemical events that underlie animal development.

## Author Summary

The differentiation of cells, tissues, and organs during animal development is established by a process in which genes that control cell identity and behavior are turned on and off at specific times and places. This process is choreographed, to a large extent, by a collection of proteins known as transcription factors that bind to specific sequences in DNA and thereby modulate the expression of neighboring genes. Because of the central role that transcription factors play in shaping organismal form and function, they have long been suggested to be major players in phenotypic evolution. However, we have a poor understanding of how changes to DNA affect transcription factor binding in living systems. Here, we use a combination of biochemical and genomic techniques to compare, between two closely related species of fruit flies in the genus *Drosophila*, the binding of six transcription factors that help establish the characteristic segments that form along the anterior-posterior (head to tail) axis in developing flies. We show that the patterns of transcription factor binding between these closely related species are broadly conserved, consistent with the nearly identical development and appearance of these species. However, we also show that, whereas the DNA changes that have accumulated between these species in the five million years since their divergence—roughly one difference per 10 basepairs—have not altered the locations where these factors bind, they have had a considerable effect on the amount of factor bound at each site across a population of embryos. We can trace these quantitative differences in binding to the gain and loss of the short sequences known to be preferentially recognized by these factors, giving us key insights into the effect that sequence changes have on the biochemical events that underlie animal development.

## Introduction

Despite four decades of interest in the evolution of transcriptional regulation, we still have a poor understanding of the molecular bases for regulatory divergence and the constraints under which *cis*-regulatory sequences evolve. Most regulatory sequences appear to be under strong selection to maintain their transcriptional output, and as a result, binding sites for the sequence-specific transcription factors that regulate mRNA synthesis are preferentially conserved [1],[2]. However, even in regulatory sequences with highly conserved function, transcription factor binding sites can be gained and lost over time at a high rate, leading to considerable differences in the composition and arrangement of binding sites between even closely related species [2]–[10]. Whether and how this binding site turnover affects transcription factor binding, and what the consequences of changes in binding on transcription might be, remains unknown.

After years in which the study of regulatory evolution was primarily a computational exercise, a series of recent studies have compared genome-wide in vivo binding of transcription factors in the same conditions or tissues of related species [11]–[14]. Among yeasts of the genus *Saccharomyces* [11],[12] and between human and mouse [13],[14], a substantial fraction of experimentally observed interactions between transcription factors and DNA are species-specific. While these differences could, in principle, be due to divergence of transcription factors and other trans-acting factors, binding differences appear to be driven primarily in *cis* [13], suggesting that differences in the sequences, and not the factors binding to them, drive the divergence in binding. Species-specific binding is generally associated with the gain/loss of sequence motifs recognized by the relevant factor [11],[14], although the correlations are weak.

Here we examine how the binding of a group of six factors that direct temporal and spatial patterns of gene expression along the anterior-posterior (A-P) axis during early development differs between *Drosophila melanogaster* and its sister species *D. yakuba*. These two species, whose genomes have been fully sequenced [15],[16], diverged only five million years ago [17]. They are separated by a molecular distance less than half that between mouse and human [18], and *D. yakuba* orthologs of virtually all *D. melanogaster* genomic regions can be readily identified and aligned. Though there are some subtle changes in the levels of expression of key regulators between these species (our unpublished data), there is little difference in either their spatial expression patterns or those of their targets, a product at least in part of strong selection to maintain them [10].

In our earlier work on the binding of these factors in *D. melanogaster*, we showed that they bind to an overlapping set of thousands of genomic regions in vivo [19],[20], as has subsequently been observed for many other animal transcription factors [21]. A wealth of evidence suggests that, at least in *D. melanogaster*, and probably generally, only the several hundred most highly bound regions are directly involved in transcriptional regulation, with the remainder having a different, or more likely no, function [19],[20].

Thus these two fly species provide an ideal opportunity to study the effects of modest sequence divergence on transcription factor binding, its origins in changes in genomic sequence, and its functional consequences. We expected binding differences between *D. melanogaster* and *D. yakuba* to be more modest than those observed between mouse and human, or between *Saccharomyces* species. However, we hoped that the more modest differences in their genomes would improve our ability to associate sequence and binding divergence, and that our earlier work establishing the relationship for these factors between binding levels and regulatory function would provide an invaluable context for analyzing the functional consequences of the binding differences we observe.

## Results

We collected embryos spanning the hour immediately preceding gastrulation, during which the regulatory events that initiate patterning along the A-P axis occur, from large laboratory populations of *D. melanogaster* (Oregon R) and *D. yakuba* (Tai8E2), and immediately immersed them in formaldehyde to covalently stabilize protein-DNA interactions. We isolated chromatin from each species, and immunoprecipitated bound regions with affinity purified rabbit polyclonal antibodies raised against the *D. melanogaster* versions of the key A-P regulators: Bicoid (BCD), Hunchback (HB), Krüppel (KR), Giant (GT), Knirps (KNI), and Caudal (CAD). We sequenced recovered fragments on an Illumina Genome Analyzer II, mapped reads to the reference genomes of each species using Bowtie [22], and calculated fragment coverage based on the average fragment length in the immunoprecipitated material (Table 1 gives statistics on the numbers of sequenced and mapped tags for each experiment in both genomes). We normalized the signal between species so that the average binding across a diverse set of known targets of these factors was equal, and projected the normalized binding signals from each species onto the coordinates of a whole-genome pairwise alignment computed using Mercator [23] and FSA [24].

**Table 1:** Sequencing and mapping statistics.

Antibody	Average Fragment Length	Tags (*D. melanogaster*)	Fraction Mapped	Tags (*D. yakuba*)	Fraction Mapped
BCD1	225	16,937,253	57.2%	6,394,260	44.6%
HB1	225	6,047,901	57.4%	4,495,105	36.5%
HB2	225	5,765,064	52.3%	4,317,045	35.6%
KR1	250	8,943,424	57.9%	10,405,884	38.3%
KR2	250	8,411,030	60.3%	10,209,264	44.1%
GT2	225	8,005,966	58.7%	6,766,174	54.7%
KNI2	225	7,431,816	55.5%	6,231,400	47.0%
CAD1	250	8,272,512	56.8%	9,822,743	42.6%
Input	225	25,120,853	55.5%	10,430,577	53.4%
Input	250	8,653,362	61.0%	6,167,546	51.6%

We required that sequenced tags map uniquely to the genome with at most one mismatch. The Input controls were segregated based on the average fragment length (225 or 250 bp); identical fragment lengths were used in both species for particular antibodies.

**Figure 1:**
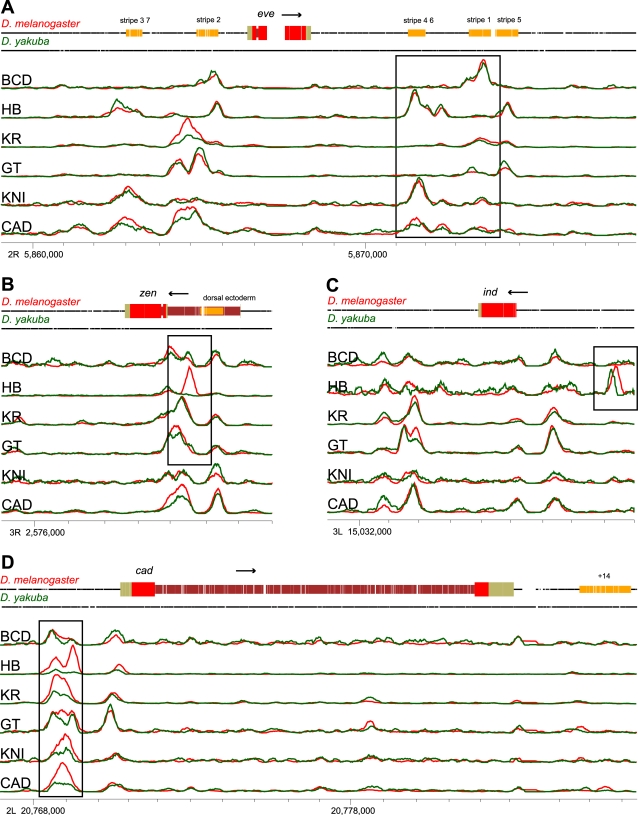
Modes of *cis*-regulatory conservation and divergence. Representative loci showing (A) broad conservation of binding, (B) complete gain and loss of binding peaks, (C) shifts in binding site location, and (D) changes in peak strength with peak location preserved. The line plots show binding to orthologous sequences in *D. melanogaster* (red) and *D. yakuba* (green), along with gene models and known regulatory elements in *D. melanogaster* (top track), where the binding signal is the inferred fragment density. Gaps in the black lines (top two tracks) for each species indicate gaps in the pairwise alignment of the two genomes. The plots are in alignment coordinates, and the chromosome positions indicated with tick marks are sequence coordinates in *D. melanogaster* (FlyBase release 5). Levels of binding were scaled for each factor and panel as appropriate for display and cannot be compared between factors or panels.

We began our analysis of binding divergence by examining previously identified targets of these six factors (Figure 1) [19]. Overall, binding to these loci is remarkably similar between species (Figure 1A), with both bound regions and their relative binding intensities similar for most factors across most loci (we note that the normalization did not consider the pattern of binding—just levels across the locus). Several types of binding divergence are evident, including the gain or loss of binding (Figure 1B), shifts in the precise location of binding (Figure 1C), and changes in the height, but not location, of binding peaks (Figure 1D). Note that with only two species it is impossible to determine whether features found in one species but not the other represent gains or losses relative to the common ancestor.

To get a comprehensive picture of this variation, we identified genomic regions significantly bound by each factor independently in both species using MACS [25] with total chromatin as controls (“Input” controls). While the signal-to-noise ratio was higher in *D. melanogaster* than in *D. yakuba*, yielding more detected peaks in *D. melanogaster* for all factors (Table 2), the relative numbers of peaks identified for each factor were similar in the two genomes. For each bound region in each species we quantified the number of sequence reads observed in the region in the source species and in the orthologous region of the other species.

**Table 2:** Gain and loss of peaks.

Factor	Species	Total Peaks Called by MACS	Peaks Absent in Ortholog (A-P Genes)	Peaks Absent in Ortholog (Non A-P Genes)
BCD	*D. melanogaster*	2,004	1/95 (1.0%)	25/1,723 (1.5%)
	*D. yakuba*	660	1/55 (1.8%)	9/567 (1.6%)
HB	*D. melanogaster*	4,434	4/123 (3.3%)	251/3,951 (6.4%)
	*D. yakuba*	1,581	6/73 (8.2%)	205/1,447 (14.2%)
KR	*D. melanogaster*	6,209	4/158 (2.5%)	150/5,599 (2.7%)
	*D. yakuba*	3,328	1/122 (0.8%)	104/3,106 (3.3%)
GT	*D. melanogaster*	2,815	1/117 (0.9%)	72/2,471 (2.9%)
	*D. yakuba*	2,508	2/102 (2.0%)	26/2,322 (1.1%)
KNI	*D. melanogaster*	547	1/55 (1.8%)	15/448 (3.3%)
	*D. yakuba*	377	0/40 (0.0%)	1/316 (0.3%)
CAD	*D. melanogaster*	4,304	2/129 (1.6%)	89/3,894 (2.3%)
	*D. yakuba*	1,870	1/98 (1.0%)	31/1,674 (1.9%)

Peaks were called as absent if the binding signal was reduced 10-fold or more in its ortholog. The denominators include only peaks where orthologs could be identified.

Before analyzing species-specific differences in binding in detail, we sought to establish that observed differences between *D. melanogaster* and *D. yakuba* were due to bona fide interspecies differences in binding, and not experimental noise or bias. As in our earlier work [19], we performed chromatin immunoprecipitation (ChIP) with antibodies recognizing different domains of several of the targeted proteins. Antibodies recognizing the N and C terminal domains of HB and KR give nearly identical results in both species, with correlations of 0.99 and 0.97 over called peaks for these antibodies in *D. melanogaster* and correlations of 0.98 and 0.94 in *D. yakuba* (Figure S1). In contrast, correlations of the binding levels for the same antibody between species range from 0.57 to 0.75, demonstrating that the binding differences are not due to experimental noise. It is also highly unlikely that these difference arise from differential affinity of the antisera for transcription factors from the two species, as there are three or fewer amino acid changes between the species for five of the six factors (KR has more than 10).

We were also concerned that differences in sequence composition or chromatin state might interact with the sequencing protocol to produce apparent interspecies differences in binding. To evaluate this, we examined genome-wide variation in the total chromatin sequencing signal (“Input” control). There was no correlation between the Input signal and binding in the individual species (Figures S2 and S3) and only a weak correlation between interspecies differences in ChIP and Input signals (from 0.04 to 0.14; Figure S4). This latter correlation is likely due to interspecies differences in chromatin state and corresponding differences in fragmentation [26], but is too weak to explain the observed differences in factor binding.

### Quantitative Changes Dominate Binding Differences between *D. melanogaster* and *D. yakuba*

Unlike in the yeast and mammalian studies described above, the gain or loss of bound regions between *D. melanogaster* and *D. yakuba* was rare, with fewer than 1% to 5% of peaks (depending on the factor) found in one species clearly absent or displaced in the other (Table 2). The rate of gain/loss near known targets of the A-P factors was similar to the genome-wide rate (Table 2).

**Figure 2:**
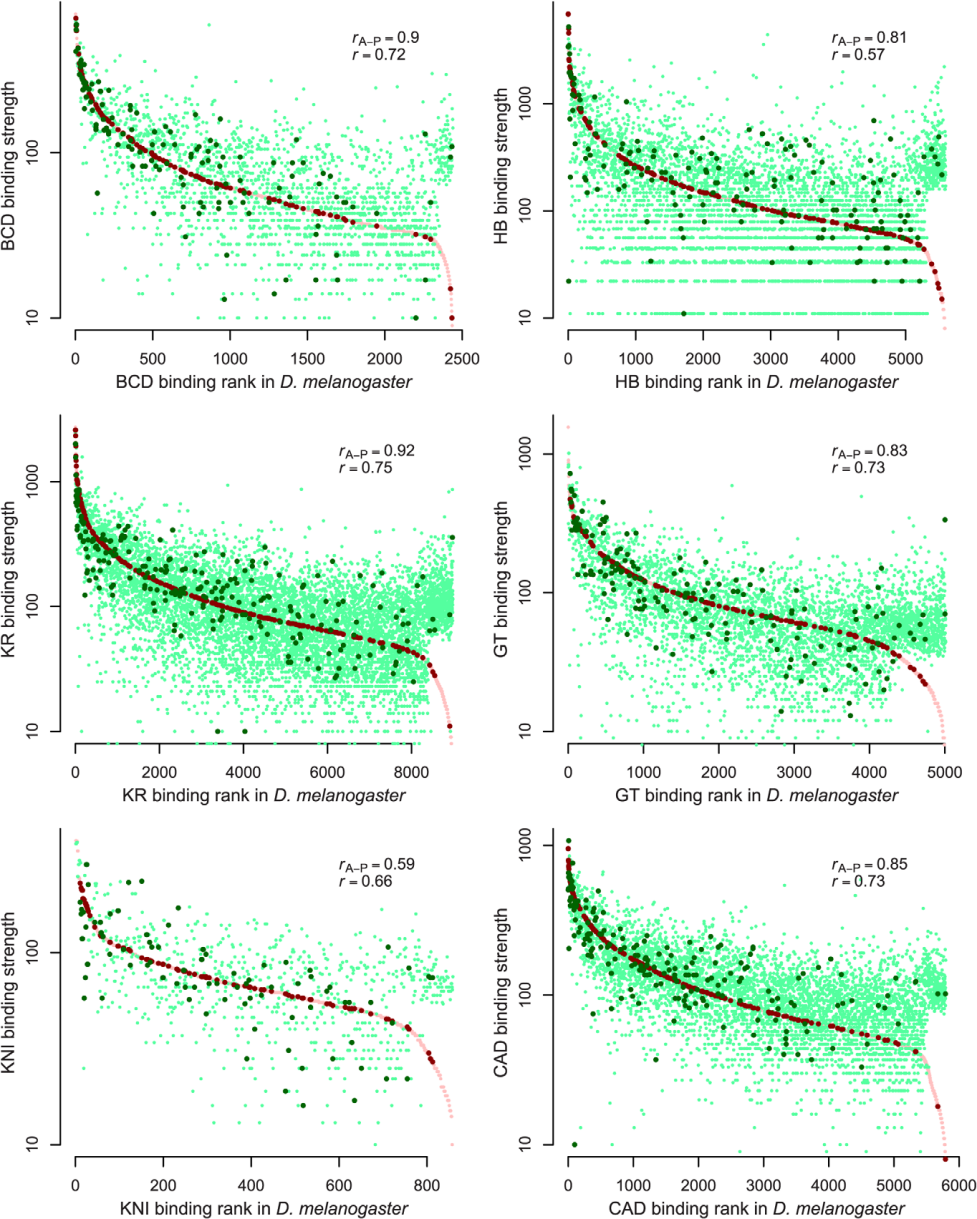
Quantitative variation in binding between species. Comparison of binding levels in *D. melanogaster* and *D. yakuba* for all identified bound regions. For each peak called in either species, we plotted the corresponding binding strengths in red for *D. melanogaster* and green for *D. yakuba*; dark colors indicate peaks near known targets of A-P regulation. Peaks are ordered left-to-right on the *x*-axis according to their binding ranks in *D. melanogaster*, and binding strengths in both genomes are plotted in log scale on the *y*-axis (binding units are arbitrary). Binding strength is well-conserved for both peaks within 10 Kb of the 5′ end of genes known to be regulated by A-P factors (“*r*_A-P_”) and those that are not (“*r*”) (list of A-P target genes given in Methods).

**Figure 3:**
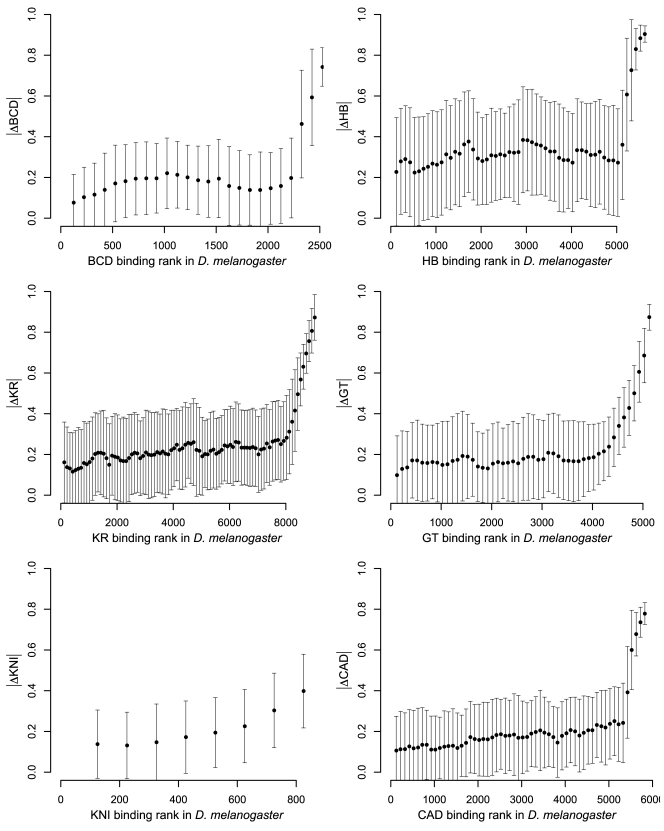
Fractional binding divergence is largely independent of binding strength. Comparison of the fractional binding divergence, computed as |*D. melanogaster* − *D. yakuba*| / (*D. melanogaster* + *D. yakuba*), with total levels of binding. Each plotted point corresponds to the median fractional binding divergence for overlapping cohorts of 250 peaks, and the error bars show the standard deviation of the fractional binding divergence within these cohorts. Note that the sharp increases at the right-hand sides of the plots correspond to peaks that are present in *D. yakuba* but not in *D. melanogaster*. Figure S7 shows the same data but displays the fractional binding divergence for every peak rather than binning by cohort (as here).

The measured binding at orthologous regions bound in both species varied considerably (Figures 2, S5, and S6) both in the highly bound regions that our previous studies suggested are functional targets of these factors [19],[20] and in the poorly bound regions that likely are not. The more highly bound regions showed a greater total variation in binding (Figure S7), with the normalized divergence (difference in binding over average binding level) roughly constant across binding levels (Figures 3 and S8) and relative to annotations (Figure S9).

The divergence was marginally lower within the 44 characterized *D. melanogaster cis*-regulatory modules (CRMs) known to be targeted by one or more of these factors (correlation *r*_A-P_ from 0.62 to 0.91 compared to 0.57 to 0.75) [27] and in peaks near genes (within 10 Kb of the 5′ end) known to be regulated by these A-P factors (correlation *r*_A-P_ from 0.59 to 0.92, depending on the factor).

### Binding Site Turnover Is a Major Source of Quantitative Variation in Binding

We sought to determine the extent to which sequence changes in the bound regions drove quantitative differences in binding. We first examined overall measures of sequence divergence. Levels of single-nucleotide divergence (sequence identity) and frequency of insertions and deletions in the 100 base pairs centered on the inferred peak of binding exhibited only low to moderate correlations with binding divergence (0.07 to 0.24; Figures S10 and S11), consistent with our expectation that changes to specific short sequences, rather than entire regions, would have a disproportionate effect on binding.

**Figure 4:**
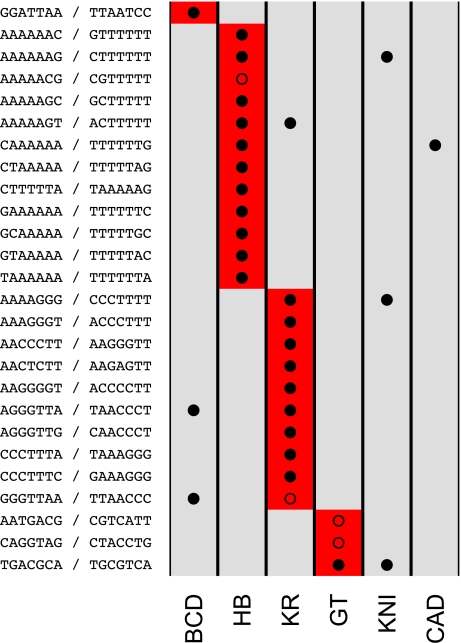
Divergence is driven by turnover of transcription factor binding sites. We identified a total of 26 divergence-driving words (7 bp) for BCD (1), HB (12), KR (10), and GT (3). A red box in a (row, column) entry indicates that the corresponding word (row) was identified as a DDW for a particular factor (column); similarly, a solid circle in a (row, column) indicates that a word (row) matches the DNA-binding specificity of a factor analyzed here (column), and an empty circle in a (row, column) indicates that a word (row) identified as a DDW for a particular factor (column) matches the specificity of an A-P transcription factor (plus Zelda), other than the six analyzed here, as characterized by [29]. Sequence motifs and their reverse complements are shown in the row labels.

We next sought to identify short sequences (e.g., transcription factor binding sites) whose gain or loss was associated with changes in binding levels. We devised an unbiased statistical approach that assessed the impact on binding of changes to a short sequence (word) by comparing the distribution of binding intensities in all bound regions where the word was conserved to the distribution in all bound regions where the word was present in one species but not the other (defining bound regions as the 100 bp centered on peaks of maximal binding intensity). If alterations to a word affect binding, then these distributions should be different. We identified such words (which we call divergence-driving words, or DDWs) by comparing the conserved and non-conserved distributions for all 16,384 words of length 7 bp and picking those that showed a statistically significant difference. We found DDWs for four of the six factors, and in each case, virtually all of these DDWs matched the known sequence specificities of the corresponding factor (Figure 4).

To quantify the fraction of binding divergence that is explained by the DDWs, we developed a method that used the gain and loss of DDWs to predict binding divergence between the species. For each factor for which we had identified DDWs, we built a simple linear model relating the divergence of DDWs in a bound region to interspecies difference in binding at that bound region. In the model, each divergent DDW in a bound region contributed a fixed amount to the predicted binding difference, with the effect of multiple divergence DDWs adding independently. The contribution of each DDW was determined by a regression using the least angle regression method [28] with extensive cross-validation (see Methods).

The correlations between predicted and observed divergence in binding of single factors across all peaks with at least one DDW in the two genomes ranged from 0.3 for HB to 0.41 for BCD (Figures S12–S27). While far from perfect, these correlations demonstrate that changes in a highly restricted collection of sequences (for example, BCD has only a single 7 bp DDW) drive an appreciable fraction of binding divergence between species. We additionally performed the same predictions using words derived from the in vitro factor binding specificities described by [29]. The correlations between predictions and observations ranged from 0.18 for HB to 0.39 in BCD, similar to or lower than the correlations resulting from our DDWs (unpublished data).

**Figure 5:**
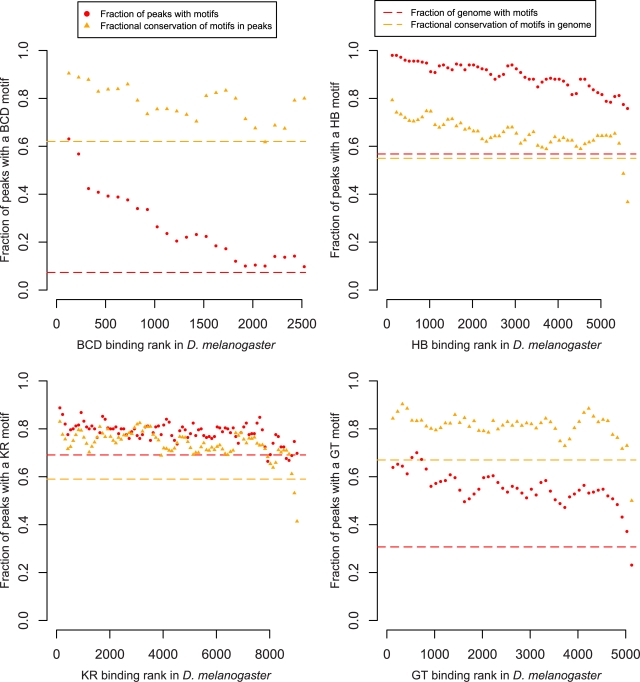
Enrichment and conservation of divergence-driving words. With the exception of BCD, we observed only a weak relationship between binding strength and enrichment (red) and conservation (yellow) of motifs identified for single factors, despite our expectation that strongly bound regions would be subject to greater functional constraint. The fraction of peaks containing one or more DDWs in *D. melanogaster* (red circles) decreased quickly with binding strength for BCD only, and was consistently higher than the background across the genome (red dashed line) for all factors. Notably, the fraction of these motifs that were conserved between *D. melanogaster* and *D. yakuba* (orange triangles) was largely independent of binding strength, and was consistently higher than the background levels of conservation of these motifs across the genome (orange dashed line). Motif enrichment (6 bp DDWs) in *D. melanogaster* and conservation in the two genomes were calculated for overlapping cohorts of 250 peaks.

We investigated whether the lack of a strong relationship between probable enhancer function and quantitative conservation of binding was associated with similar trends at the sequence level. For each factor for which we identified DDWs, we quantified motif enrichment and conservation as a function of the level of transcription factor occupancy in *D. melanogaster*. Motif enrichment and conservation were elevated within bound regions above background levels across the genome (Figure 5). The fraction of peaks with motifs showed a weak dependence on binding levels, with the most strongly bound regions exhibiting the greatest density of motifs. The level of conservation of these motifs was weakly correlated with overall binding levels, consistent with our observation that quantitative divergence in binding strength decreased slightly near genes regulated by these factors.

### Binding Divergence of All Six Factors Is Strongly Correlated

In our initial comparison of binding between species, we noticed that increases in binding of a single factor were often correlated with increases in binding of many other factors (Figures S28–S33). For example, changes in the binding of KR correlated with changes in the binding of other factors with *r* = 0.36 (KNI) to 0.62 (CAD), and such coordinated changes are recapitulated for all pairs of factors. This widespread correlated change suggests a factor-independent mode of binding divergence.

**Figure 6:**
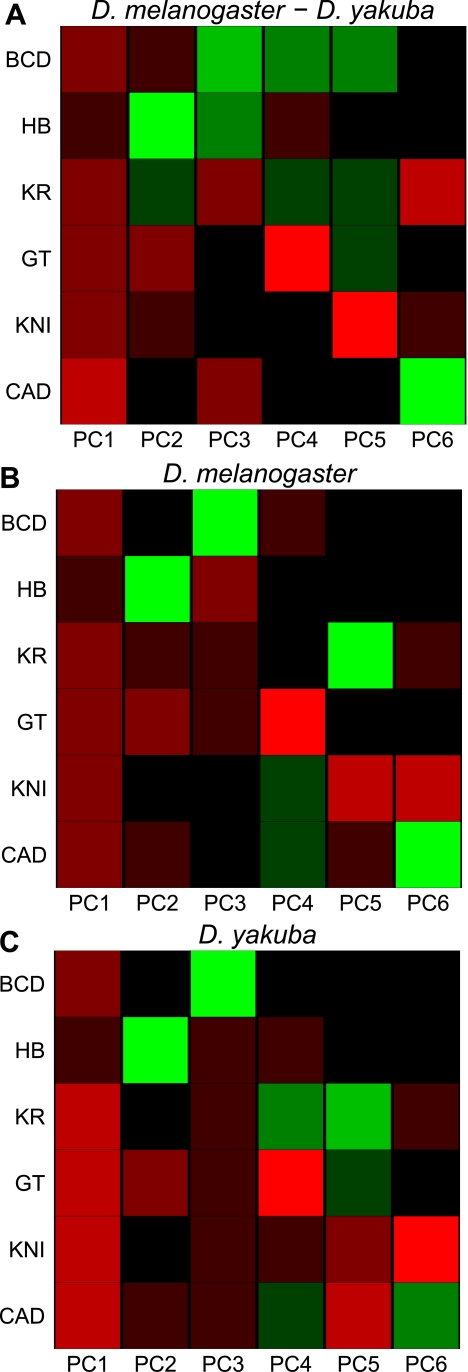
Principal component analysis of binding of all factors. PCA of (A) the relative change in binding strength across all peaks, and the binding in (B) *D. melanogaster* and (C) *D. yakuba*. Each row represents a factor, and each column is a principal component of the relevant data. The color represents the sign (red positive, green negative) and magnitude (color intensity) of each value in each principal component vector. Note that in each case the sign of the first principal component is the same for all six factors, indicating that the dominant driver of both interspecies divergence and quantitative variation within single species is a coordinated change in binding strength of all factors. This effect, which could be due to changes in chromatin state, explained 38% of the variation between species, and 62% (*D. melanogaster*) and 55% (*D. yakuba*) of the variation within species.

To obtain an unbiased assessment of the extent of these correlated changes in binding, we quantified binding divergence for all six factors in all regions significantly bound by any factor and performed principal component analysis (PCA), a method for analyzing variation between many factors simultaneously rather than only pairs of factors, on these data (Figure 6A). The first principal component, which represents the most significant axis of variation in the dataset, has the same direction and similar magnitude for all six factors, demonstrating that a pan-factor coordinated binding shift is the dominant driver of A-P factor binding divergence (this principle component explains 38% of the overall variation in binding between the species). A similar effect was observed when we performed PCA on the binding levels in each species independently (Figure 6B and 6C), suggesting that a common effect is responsible for much of the variation in binding both between species and within a single genome.

The single-genome PCA revealed several interesting factor-specific correlations: increases in binding of the repressor GT are associated with decreases in binding of the activator HB (PC2 in Figure 6B), increases in HB are associated with decreases in BCD (PC3 in Figure 6B), etc. As expected, given the overall similarity of binding between the species, the single-genome PCA analyses of *D. melanogaster* and *D. yakuba* yielded essentially identical results.

**Figure 7:**
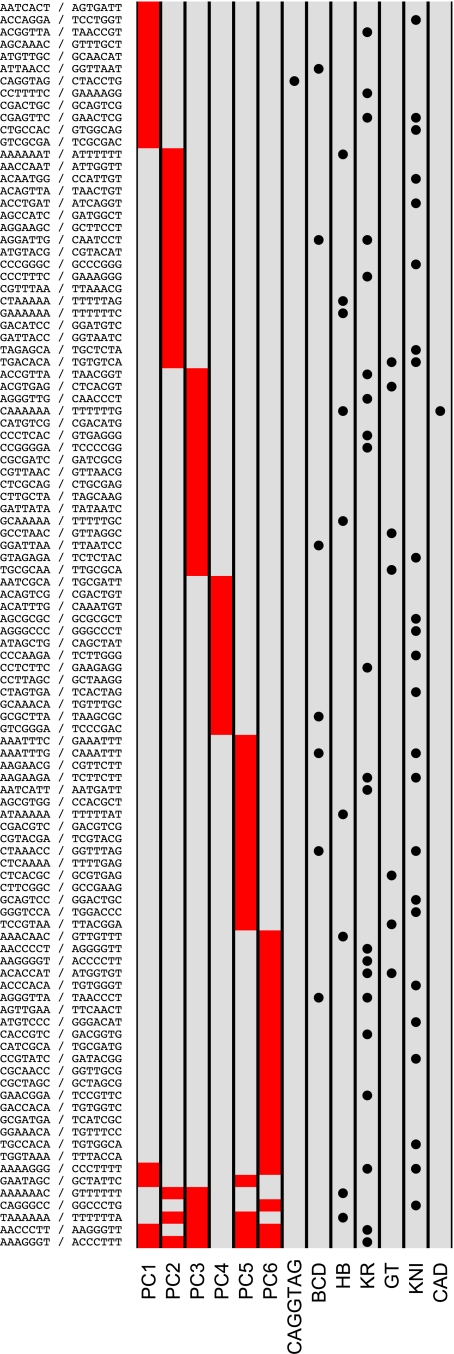
Divergence-driving words for principal components. We identified divergence-driving words for each of the principal components in Figure 6A. Few words are shared by the different principal components, suggesting that distinct sets of motifs and A-P regulators govern the different patterns of variation revealed by PCA. The CAGGTAG binding site for the early zygotic activator Zelda drives divergence of coherent binding of all factors (principal component 1). A red box in a (row, column) entry indicates that the corresponding word (row) was identified as a DDW for a particular principal component (column); a solid circle in a (row, column) indicates that a word (row) matches the DNA-binding specificity of a factor analyzed here, plus Zelda (column). In contrast to Figure 4, where empty circles are used to indicate matches to A-P factors other than the six analyzed here, no empty circles are shown here because all words match the specificities of one or more additional A-P regulators, as characterized by [29]. Sequence motifs and their reverse complements are shown in the row labels.

To investigate whether the features captured by these different principal components are related to specific sequences, we applied the same motif discovery method described above to projections of the binding data along each of the principal components shown in Figure 6A. We discovered substantially more motifs in this analysis (Figure 7) than in the single-factor analyses, likely because of the increased statistical power derived from considering all regions bound by any, as opposed to a single, factor.

Interestingly, one of the words whose divergence is associated with the first principal component is the “TAGteam” motif, CAGGTAG *[30], the binding site for Zelda, an activator of the early zygotic genome [31]. Zelda's mechanism of action is unknown, but the strong correlation between gain and loss of its binding site with variation in changes in binding of all factors supports a direct or indirect role for Zelda in nucleosome positioning and chromatin remodeling.*

## Discussion

We have provided the first genome-wide picture of how modest levels of sequence divergence between highly morphologically similar species affect a system of coordinately acting transcription factors during animal development. The pervasiveness of changes in binding levels highlights the importance of treating transcription factor binding as a quantitative trait. This is in contrast to previous interspecies studies of in vivo binding [11],[13],[14], which focused on the gain and loss of bound regions.

Although the gain/loss of bound regions is often associated with the gain/loss of cognate binding sites, we establish here that the primary biochemical effect of binding site turnover is to alter levels of binding to existing bound regions. What remains unclear is whether and how the small changes in the amount of bound factor affect transcription, and under what circumstances such changes have demonstrable phenotypic consequences. That there are no clear differences in binding divergence between functional and non-functional targets, and that the most strongly bound (and presumably functional) regions show more absolute, and equal relative, divergence suggests that much of the variation we observe between these two species does not significantly affect organismal fitness, consistent with the observation that binding site gain and loss in active CRMs often does not result in significant changes in regulatory function [6],[10]. This is, however, far from definitive proof, and there are many alternative explanations for this observation, such as compensatory or directional selection on binding to functional regulatory sequences. Exploring the molecular and developmental consequences of quantitative variation in transcription factor binding will be an exciting avenue for future research.

Although we and others have previously described a broad correlation between factor binding in the *Drosophila* blastoderm [19],[20],[32] and other systems, we were surprised at how strong this common effect was in driving interspecies binding differences. It is tempting to speculate that this effect arises from interspecies differences in chromatin structure, which could readily produce such a uniform effect on the binding of a large collection of factors. However, the only direct evidence that chromatin differences may cause binding differences is the association of the gain/loss of CAGGTAG with the increase/decrease of the common factor signal (PC1). CAGGTAG is the binding site for the factor Zelda, a general zygotic activator of transcription with a putative association with chromatin. CAGGTAG, however, explains only a small fraction of the common signal.

Indirect cooperativity between factors, in which binding of one factor alters chromatin state and thereby facilitates the binding of other factors, may also play a significant role in binding divergence. We have examined only six of the approximately 40 transcription factors active at this stage of embryogenesis. Given the extensive cross-binding of A-P and dorsal-ventral regulators [19],[33], it is likely that changes in the binding of some of these additional factors influences the A-P factor binding.

### Lessons for Future Studies

Although *D. melanogaster* and *D. yakuba* are closely related, we were not always able to accurately identify orthologous sequences, largely due to ambiguities in the draft *D. yakuba* assembly. Even where the orthology of regions was unambiguous, and despite this close evolutionary distance, base-level alignments were frequently uncertain. Our analysis of sequence-specific effects required a precise alignment, and inevitable alignment errors will make nucleotide-level analysis of regulatory changes challenging for more distantly related species (although the alignment accuracy estimates produced by FSA may help to identify reliably aligned loci).

Several aspects of this experiment should help direct future efforts to use comparative ChIP-Seq to study the relationship between sequence and binding divergence. The widespread quantitative binding divergence between *D. melanogaster* and *D. yakuba* demonstrates that even relatively similar species can be used to study binding changes. Indeed, given the magnitude of the binding divergence that we observe, we expect there to be quantitative differences between *D. melanogaster* and more closely related species, such as *D. simulans*, as well as among *D. melanogaster* individuals. While comparisons with more distantly related species will likely reveal greater binding divergence, and will help explain how such divergence affects expression and phenotype, the difficulties with aligning genomes at this distance, and comparing embryonic stages, may render sequence-based analyses less powerful.

Even though we were working with very similar organisms, with similar timing and structure of embryonic development, there were undoubtedly subtle differences in our sampling of developmental stages in the two species. Because transcription factor binding is dynamic, such sampling differences have the potential to manifest themselves as apparent interspecies differences in binding. We do not believe this effect was significant in our data, however, as it is unlikely that this type of false-positive binding divergence would be associated with the specific sequence changes that we repeatedly observed. Nonetheless, this will be a major difficulty in future studies, especially when developmentally and morphologically different organisms are compared, as precisely those changes that make such comparisons interesting also make them far more difficult.

## Methods

### In Vivo Formaldehyde Cross-Linking of Embryos and ChIP in *D. melanogaster* and *D. yakuba*

Both *D. melanogaster* and *D. yakuba* embryos were collected from population cages for 1 h, and then allowed to develop to late stage 4 and early stage 5 before being harvested and fixed with formaldehyde. The embryos from the two species developed very similarly, and the aging times to reach the desired age were 2 h for *D. melanogaster* embryos and 1 h and 45 min for *D. yakuba* embryos. The staged embryos were harvested and cross-linked with formaldehyde, and the chromatin was isolated through CsCl gradient ultracentrifugation essentially as previously described [19].

The chromatin used for immunoprecipitation was fragmented through sonication using a Branson Sonifier 450 to an average fragment size of 225 to 250 bp, which is shorter than the average size of chromatin used in our previous ChIP-chip experiments [19]. ChIP was carried out using affinity purified rabbit polyclonal antibodies, and for two of the factors, HB and KR, two affinity purified antibodies that recognize non-overlapping parts of each factor were used. These antibodies and the ChIP procedure were identical to those described in [19].

### Sequencing of DNA from ChIP

The DNA libraries for sequencing were prepared from the ChIP reaction and from Input DNA following the Illumina protocol for preparing samples for ChIP sequencing of DNA using the reagents provided in the genomic-DNA or ChIP-DNA sample preparation kits, with some modifications. Briefly, the DNA fragments were converted to phosphorylated blunt ends using T4 DNA polymerase, Klenow DNA polymerase, and T4 polymerase kinase, a 3′ A base overhang was added using Klenow DNA polymerase exo- (3′ to 5′ exo minus), and Illumina adapters were ligated to the fragments. We carried out the PCR step for enrichment of adapter-modified DNA prior to the library size selection, and limited the amplification to 10–13 cycles to minimize the potential bias associated with PCR amplification. After the amplification step, we size-selected DNA fragments of 150–500 bp (including the adapter sequence) for BCD, HB, GT, and KNI samples, and 200–500 bp for KR and CAD. The DNA library was quantified by QPCR using ABI Power SYBR green PCR master mix and pair primers that match the adapter sequences. We used a Solexa DNA library, which we generated with known concentration as a standard. Due to the extreme sensitivity, the DNA used in the reactions ranged from 0.0001–0.01 ng. The sequencing of the library DNA was performed on the Solexa/Illumina platform according to the manufacturer's instruction. Each library was analyzed in two lanes on the flow cell.

### Mapping Sequenced Tags to Genomes

We used the Apr. 2006 assembly (dm3, BDGP Release 5) of the *D. melanogaster* genome, downloaded from http://hgdownload.cse.ucsc.edu/goldenPath/dm3/bigZips/chromFa.tar.gz, and the Nov. 2005 assembly (droYak2) of the *D. yakuba* genome, downloaded from http://hgdownload.cse.ucsc.edu/goldenPath/droYak2/bigZips/chromFa.tar.gz.

We trimmed all sequenced tags to 20 bp and mapped the tags to the genomes using Bowtie v0.9.9.1 [22] with command-line options ‘-v 1 -m 1’, thereby keeping only tags that mapped uniquely to the genome with at most one mismatch. Table 1 gives statistics on the total numbers of sequenced and mapped tags for all experiments. Note that while we mapped tags to the entire genomes, we did not use the heterochromatic chromosomes or unassembled sequence for any analyses.

We used annotations from FlyBase r5.15 [34] for analyses using genes in *D. melanogaster*.

### Peak Calling

We called peaks for each experiment using MACS v1.3.5 [25] with the option ‘--*p*value 0.00001’. We used total chromatin as background controls, and set the ‘--mfold’ option to the maximum value for which MACS could find a sufficient number of paired peaks. In order to only consider peaks for which we could reliably assign orthology and to control for potential assembly errors in the draft *D. yakuba* genome, we used exonerate [35] to search for peaks whose associated sequence was duplicated in either genome. For each peak, we (1) searched for duplicated sequence in the genome where the peak was called and (2) used the whole-genome alignment to pull out the orthologous sequence in the other genome and searched for duplicates of that sequence in the other genome, which frequently indicated a potential assembly error due to the unfinished nature of the *D. yakuba* assembly. We discarded any peaks whose associated sequence was duplicated in either genome.

### Whole-Genome Alignment and Orthology Comparisons

We used a large-scale orthology mapping created by Mercator [23] to identify syntenic regions of the genomes, which were each aligned with FSA v1.11.0 with the options ‘--exonerate --softmasked --refinement -1 --mercator cons seqs.fasta’. The resulting whole-genome alignment can be downloaded here: http://www.biostat.wisc.edu/~cdewey/data/fsa_mercator_alignments/drosophila_melanogaster-5.0-drosophila_yakuba-2.0-1.0.tar.gz.

### Signal Normalization between Genomes

We first normalized the total number of sequenced tags to a fixed number for each experiment, the standard method of controlling for the variable success of amplification and sequencing. This normalization, however, is insufficient for our purposes, since it does not take into account differences in genome size and background between the species. We therefore performed an additional comparative normalization step. Assuming that the total amount of binding near known regulatory targets of the six factors studied here (A-P and D-V genes, as identified in [19] and listed below) is constant, we scaled the total number of sequenced tags in *D. yakuba* for each factor such that the total difference in inferred binding strength across the 50 most highly bound peaks in each genome (for a total of 100) within 10 kb of A-P targets was minimized (using a least-squares linear regression).

This comparative normalization procedure assumes there are no differences in the total number of molecules bound to A-P targets in the two genomes. Although this may not always be the case, we do not expect to see such global differences between such closely related species. It is also possible that by using the 50 most highly bound peaks near known A-P target genes for normalization we would underestimate variation in these genes. However, the effect of any single peak on the normalization was minimal, and the inferred divergence for any of these peaks did not change significantly when they were not included in the normalization (unpublished data).

### Binding Strength Comparison between Genomes

We assessed binding strength by estimating a fragment density by extending each sequenced tag to the average fragment length based on the selected size distribution. We modified the SynPlot program [36] to display quantitative data along an alignment in order to create the plot in Figure 1.

We compared binding between the two genomes as follows: Given a peak called in one genome, we used the whole-genome alignment to project the 100 bp containing the peak onto the other genome and computed the maximum binding strength within that homologous sequence in the other genome. Note that therefore our maximum spatial resolution when assessing binding divergence is 50 bp, implying that if, for example, a binding site is present in *D. melanogaster,* and lost in *D. yakuba* but replaced by another site 30 bp away, then we will not detect any binding divergence if the two sites are bound at similar levels.

We labeled peaks that were within 10 Kb of a gene in *D. melanogaster* known to be regulated by A-P factors as A-P target loci. We used the following list of genes: *Brk*, *D*, *Doc1*, *Doc2*, *E(spl)*, *Kr*, *Phm*, *SoxN*, *Vnd*, *bowl*, *btd*, *cad*, *croc*, *dpp*, *ems*, *eve*, *fkh*, *ftz*, *gt*, *h*, *hb*, *hkb*, *ind*, *kni*, *knil*, *noc*, *nub*, *oc*, *odd*, *opa*, *os*, *pdm2*, *pnr*, *prd*, *pxb*, *rho*, *run*, *salm*, *shn*, *sim*, *slp1*, *slp2*, *sna*, *sob*, *sog*, *ths*, *tld*, *tll*, *tsh*, *tup*, *twi*, *vn*, *wntD*, *zen*.

### Sequence Motif Identification

We identified DDWs for each factor as follows. For each word of a fixed length *k*, we identified all (non-softmasked) instances of the word (on both strands) within a 100 bp window centered on the empirical maximum of peaks called in *D. melanogaster* for that factor. We then accumulated two distributions of binding strength divergence (*D. melanogaster* − *D. yakuba*) for the word, *p_cons_* and *p_div_*, with *p_cons_* consisting of instances where the word was exactly conserved in *D. yakuba* and *p_div_* consisting of instances where the word was diverged in *D. yakuba*. We used a non-parametric statistical test, Kolmogorov-Smirnov test, to test for equality of distribution *p_cons_* ∼ *p_div_*. If equality of distribution could be rejected with *p*<0.01, then we called the word a candidate DDW. We then performed the identical procedure in the opposite direction, wherein we examined peaks called in *D. yakuba* and assessed the conservation of words in *D. melanogaster*, and identified a second set of candidate DDWs. We took the intersection of these two sets to obtain final lists of DDWs. We performed this procedure to identify words of length *k* = 6 and 7.

We assessed whether sequence motifs matched the known DNA-binding specificities of A-P factors with position weight matrices (PWM) from [29]. When creating Figures 4 and 7, we said that a word matched the specificity for a factor if it matched a subsequence of the corresponding PWM with ln (*p* value) <−4 as reported by Patser [37].

### Prediction of Binding Divergence

We used the Least Angle Regression (LARS) algorithm [28], implemented in the package lars for R [38], to learn a linear model of binding divergence using DDWs of length *k* = 6. We performed 5-fold cross-validation to estimate the mean-squared prediction error (MSE) associated with each value of the lasso regularization parameter β and then chose the model given by the β that yielded the lowest MSE. This cross-validation procedure helps to prevent the over-fitting characteristic of standard least-squares linear regression, making the correlations that we estimated robust to generalization error.

In order to ensure that (1) the DDWs that we identified truly have predictive value and (2) that the correlations reported are not due solely to base-composition effects, we randomly shuffled the nucleotides of each DDW to create a set of shuffled words with unchanged base composition, and then built a predictive model using these shuffled words. Models constructed using these shuffled words had no predictive value, indicating that the correlations that we report for our DDWs are not statistical artifacts. Figures S12–S27 show lasso variable selection curves and cross-validation curves for all values of β, as well as scatterplots of predicted and observed binding divergences, for predictive models constructed using our DDWs as well as their shuffled counterparts. The cross-validation curves make clear that while the DDWs are correlated with binding strength, the shuffled words are not: MSE decreases as more DDWs are included into the model, indicating the gain and loss of these words correlates with changes in observed binding strength, whereas MSE increases as more shuffled words are included into the model, indicating that these words are uncorrelated with binding. This provides clear evidence that our cross-validation procedure correctly chooses the model with the minimum generalization error, for example, that the models are not over-fit to the data.

We performed an identical analysis using words derived from the in vitro binding specificity data described in [29]. We enumerated all *k*-mers that matched a subsequence of the corresponding PWM with ln (*p* value) <−8 as reported by Patser [37], identifying four 6-mers for BCD, HB, and GT and sixteen 6-mers for KR, and then used the learning procedure described above to learn models of binding divergence using these words.

### PCA

We calculated binding strengths of the six factors across all called peaks, subtracted the empirical means for each factor, and scaled the data for each factor such that it had unit variance. We used the singular value decomposition routine in IT++, a linear algebra library for C++, to perform PCA, and created heatmaps of the PCA results using a modified version of the aspectHeatmap function in the ClassDiscovery package.

In order to confirm that the putative chromatin signal represented by the first principal component did reflect coherent increases and decreases in binding of all six factors in our data, we randomly interchanged the measured binding strengths for a single factor across called peaks while holding all others unchanged (Figure S34, panels A–F) and similarly randomly interchanged the binding strengths of all factors (Figure S34, panel G), thereby removing spatial correlations between the binding of single factors and the other five (Figure S34, panels A–F) and removing spatial correlations between the binding of any factors (Figure S34, panel G). As expected, the chromatin signal disappeared after performing any of these transformations on the data.

We identified sequence motifs associated with interspecies divergence of each principal component using the same procedure described above, but with the data projected along the principal component of interest. For each principal component, we accumulated the distributions *p_cons_* and *p_div_* across all peaks called for any of the six factors.

### Data Availability

All sequence reads from the experiments described are available from the NCBI's GEO database with accession number GSE20369. Processed datasets, including mapped reads, called regions and peaks, *D. melanogaster* − *D. yakuba* alignments, and all software described here, are available at http://rana.lbl.gov/data/melyak.

## Supporting Information

Figure S1 **Correlation between binding levels for peaks called for distinct antibodies (A) HB in *D. melanogaster*, (B) KR in *D. melanogaster*, (C) HB in *D. yakuba*, and (D) KR in *D. yakuba*.** Correlations rounded to two significant digits.
(1.75 MB PDF)

Figure S2 **Correlations between Input and ChIP signals for *D. melanogaster.*** Binding strength (goldenrod) and Input signal (black) for each peak called in *D. melanogaster*.
(2.16 MB PDF)

Figure S3 **Correlations between Input and ChIP signals for *D. yakuba*.** Binding strength (goldenrod) and Input signal (black) for each peak called in *D. yakuba*.
(1.14 MB PDF)

Figure S4 **Correlations between divergence in Input and ChIP signals.** Divergence in binding strength and Input signal for peaks called in either species.
(1.65 MB PDF)

Figure S5 **Comparison of binding between *D. melanogaster* and *D. yakuba*.** Scatterplots of binding strengths at peaks called in either species for each factor. The *x*- and *y*-axes show the logarithms of the binding strengths in *D. melanogaster* and *D. yakuba*.
(1.63 MB PDF)

Figure S6 **Quantitative variation in binding between species.** Comparison of binding levels in *D. melanogaster* and *D. yakuba* for all identified bound regions. For each peak called in either species, we plotted the corresponding binding strengths in red for *D. melanogaster* and green for *D. yakuba*; dark colors indicate known *cis*-regulatory modules (compare with Figure 2, where dark colors indicate peaks near genes regulated by A-P factors). Peaks are ordered left-to-right on the *x*-axis according to their binding ranks in *D. melanogaster* and binding strengths in both genomes are plotted in log scale on the *y*-axis (binding units are arbitrary).
(3.26 MB PDF)

Figure S7 **Absolute binding divergence as a function of binding strength.** Absolute binding divergence, computed as |*D. melanogaster* − *D. yakuba*|, for overlapping cohorts of 250 peaks called in either species. The error bars indicate the standard deviations of each cohort. As with Figure S7, the rising tails are due to peaks called in *D. yakuba* with little or no binding in *D. melanogaster*.
(1.64 MB PDF)

Figure S8 **Fractional binding divergence for all peaks.** Fractional binding divergence, computed as (*D. melanogaster* − *D. yakuba*) / (*D. melanogaster* + *D. yakuba*), for peaks called in either species. The downward trend of the datapoints from left to right is due to our comparative normalization procedure, which is based only on the ∼100 most highly bound regions near genes regulated by the A-P factors, and the ordering of the *x*-axis by binding rank in *D. melanogaster*. Peaks that are highly bound in *D. yakuba* but not in *D. melanogaster* tend to be placed on the right-hand side of the plot (since they are of low rank in *D. melanogaster*), and furthermore frequently have a negative fractional binding divergence since they are highly bound in *D. yakuba* but not in *D. melanogaster*. Similarly, the tails to the right correspond to peaks called in *D. yakuba* with little or no binding in *D. melanogaster*. Fractional binding divergence is similar for both highly bound and weakly bound regions. Figure 3 is similar to this figure, but shows median fractional binding divergence for cohorts of peaks rather than for every peak.
(0.04 MB PDF)

Figure S9 **Relationship between fractional binding divergence and distance to nearest gene.**
(0.08 MB PDF)

Figure S10 **Correlation between single-nucleotide divergence and fractional binding divergence.** Best-fit linear models shown.
(1.64 MB PDF)

Figure S11 **Correlation between insertion/deletion frequency and fractional binding divergence.** Best-fit linear models shown.
(1.63 MB PDF)

Figure S12 **Linear model of BCD binding divergence.** Linear model of binding divergence using divergence-driving words (6 bp) identified for BCD. Left panel shows the model coefficients for each word as a function of the lasso regularization parameter β; right panel shows the mean-squared prediction error associated with each value of β based on a 5-fold cross-validation procedure. The constant decrease in prediction error indicates that including more words in the linear model helps with prediction, suggesting that the DDWs that we identified do guide factor binding. Figure created by the lars package in R.
(0.02 MB PDF)

Figure S13 **Accuracy of linear model predictions for BCD.** Scatterplot of measured and predicted binding divergence for BCD. Predictions used the linear model illustrated in Figure S12, where model coefficients were chosen to minimize the cross-validation prediction error.
(0.05 MB PDF)

Figure S14 **Linear model of HB binding divergence.** Linear model of binding divergence using divergence-driving words (6 bp) identified for HB. Left panel shows the model coefficients for each word as a function of the lasso regularization parameter β; right panel shows the mean-squared prediction error associated with each value of β based on a 5-fold cross-validation procedure. The constant decrease in prediction error indicates that including more words in the linear model helps with prediction, suggesting that the DDWs that we identified do guide factor binding. Figure created by the lars package in R.
(0.06 MB PDF)

Figure S15 **Accuracy of linear model predictions for HB.** Scatterplot of measured and predicted binding divergence for HB. Predictions used the linear model illustrated in Figure S14, where model coefficients were chosen to minimize the cross-validation prediction error.
(0.30 MB PDF)

Figure S16 **Linear model of KR binding divergence.** Linear model of binding divergence using divergence-driving words (6 bp) identified for KR. Left panel shows the model coefficients for each word as a function of the lasso regularization parameter β; right panel shows the mean-squared prediction error associated with each value of β based on a 5-fold cross-validation procedure. The constant decrease in prediction error indicates that including more words in the linear model helps with prediction, suggesting that the DDWs that we identified do guide factor binding. Figure created by the lars package in R.
(0.13 MB PDF)

Figure S17 **Accuracy of linear model predictions for KR.** Scatterplot of measured and predicted binding divergence for KR. Predictions used the linear model illustrated in Figure S16, where model coefficients were chosen to minimize the cross-validation prediction error.
(0.43 MB PDF)

Figure S18 **Linear model of GT binding divergence.** Linear model of binding divergence using divergence-driving words (6 bp) identified for GT. Left panel shows the model coefficients for each word as a function of the lasso regularization parameter β; right panel shows the mean-squared prediction error associated with each value of β based on a 5-fold cross-validation procedure. The constant decrease in prediction error indicates that including more words in the linear model helps with prediction, suggesting that the DDWs that we identified do guide factor binding. Figure created by the lars package in R.
(0.06 MB PDF)

Figure S19 **Accuracy of linear model predictions for GT.** Scatterplot of measured and predicted binding divergence for GT. Predictions used the linear model illustrated in Figure S18, where model coefficients were chosen to minimize the cross-validation prediction error.
(0.18 MB PDF)

Figure S20 **Linear model of BCD binding divergence (control with shuffled words).** Linear model of binding divergence using shuffled versions of the divergence-driving words (6 bp) identified for BCD. The increase in prediction error indicates that including more shuffled words in the linear model does not help with prediction, suggesting that the shuffled DDWs that we identified do not guide factor binding. Compare with Figure S12.
(0.03 MB PDF)

Figure S21 **Accuracy of linear model predictions for BCD (control with shuffled words).** Scatterplot of measured and predicted binding divergence for BCD. Predictions used the linear model illustrated in Figure S20, where model coefficients were chosen to minimize the cross-validation prediction error. Compare with Figure S13.
(0.05 MB PDF)

Figure S22 **Linear model of HB binding divergence (control with shuffled words).** Linear model of binding divergence using shuffled versions of the divergence-driving words (6 bp) identified for BCD. The increase in prediction error indicates that including more shuffled words in the linear model does not help with prediction, suggesting that the shuffled DDWs that we identified do not guide factor binding. Compare with .
(0.10 MB PDF)

Figure S23 **Accuracy of linear model predictions for HB (control with shuffled words).** Scatterplot of measured and predicted binding divergence for BCD. Predictions used the linear model illustrated in Figure S22, where model coefficients were chosen to minimize the cross-validation prediction error. Compare with Figure S15.
(0.30 MB PDF)

Figure S24 **Linear model of KR binding divergence (control with shuffled words).** Linear model of binding divergence using shuffled versions of the divergence-driving words (6 bp) identified for BCD. The increase in prediction error indicates that including more shuffled words in the linear model does not help with prediction, suggesting that the shuffled DDWs that we identified do not guide factor binding. Compare with .
(0.36 MB PDF)

Figure S25 **Accuracy of linear model predictions with KR (control with shuffled words).** Scatterplot of measured and predicted binding divergence for BCD. Predictions used the linear model illustrated in Figure S24, where model coefficients were chosen to minimize the cross-validation prediction error. Compare with Figure S17.
(0.43 MB PDF)

Figure S26 **Linear model of GT binding divergence (control with shuffled words).** Linear model of binding divergence using shuffled versions of the divergence-driving words (6 bp) identified for BCD. The increase in prediction error indicates that including more shuffled words in the linear model does not help with prediction, suggesting that the shuffled DDWs that we identified do not guide factor binding. Compare with Figure S18.
(0.13 MB PDF)

Figure S27 **Accuracy of linear model predictions with GT (control with shuffled words).** Scatterplot of measured and predicted binding divergence for BCD. Predictions used the linear model illustrated in Figure S26, where model coefficients were chosen to minimize the cross-validation prediction error. Compare with Figure S19.
(0.18 MB PDF)

Figure S28 **Correlations of fractional binding divergence of BCD with other factors.** Correlations for fractional binding divergence, defined as (*D. melanogaster* − *D. yakuba*) / (*D. melanogaster* + *D. yakuba*).
(2.34 MB PDF)

Figure S29 **Correlations of fractional binding divergence of HB with other factors.** Correlations for fractional binding divergence, defined as (*D. melanogaster* − *D. yakuba*) / (*D. melanogaster* + *D. yakuba*).
(3.24 MB PDF)

Figure S30 **Correlations of fractional binding divergence of KR with other factors.** Correlations for fractional binding divergence, defined as (*D. melanogaster* − *D. yakuba*) / (*D. melanogaster* + *D. yakuba*).
(4.21 MB PDF)

Figure S31 **Correlations of fractional binding divergence of GT with other factors.** Correlations for fractional binding divergence, defined as (*D. melanogaster* − *D. yakuba*) / (*D. melanogaster* + *D. yakuba*).
(3.08 MB PDF)

Figure S32 **Correlations of fractional binding divergence of KNI with other factors.** Correlations for fractional binding divergence, defined as (*D. melanogaster* − *D. yakuba*) / (*D. melanogaster* + *D. yakuba*).
(1.89 MB PDF)

Figure S33 **Correlations of fractional binding divergence of CAD with other factors.** Correlations for fractional binding divergence, defined as (*D. melanogaster* − *D. yakuba*) / (*D. melanogaster* + *D. yakuba*).
(3.30 MB PDF)

Figure S34 **PCA controls.** PCA after (A–F) randomly interchanging the measured binding strengths for single factors across called peaks while holding all others unchanged, and (G) similarly randomly interchanging the binding strengths of all factors. These operations remove spatial correlations between the binding of single factors and the other five (A–F) and spatial correlations between the binding of any factors (G). As expected, the chromatin signal disappeared after performing any of these transformations on the data.
(0.43 MB PDF)

## Abbreviations

A-P: anterior–posterior
BCD: Bicoid
CAD: Caudal
ChIP: chromatin immunoprecipitation
CRM: *cis*-regulatory module
DDW: divergence-driving word
GT: Giant
HB: hunchback
kb: kilobase
KNI: Knirps
KR: Krüppel
LARS: Least Angle Regression
MSE: mean-squared prediction error
PCA: principal component analysis
PWM: position weight matrices
