# Supplementary figures and images for: Binding Site Turnover Produces Pervasive Quantitative Changes in Transcription Factor Binding between Closely Related Drosophila Species
Source: PLoS Biol. 2010 Mar 23;8(3):e1000343. doi: 10.1371/journal.pbio.1000343 (PMC2843597; doi:10.1371/journal.pbio.1000343)

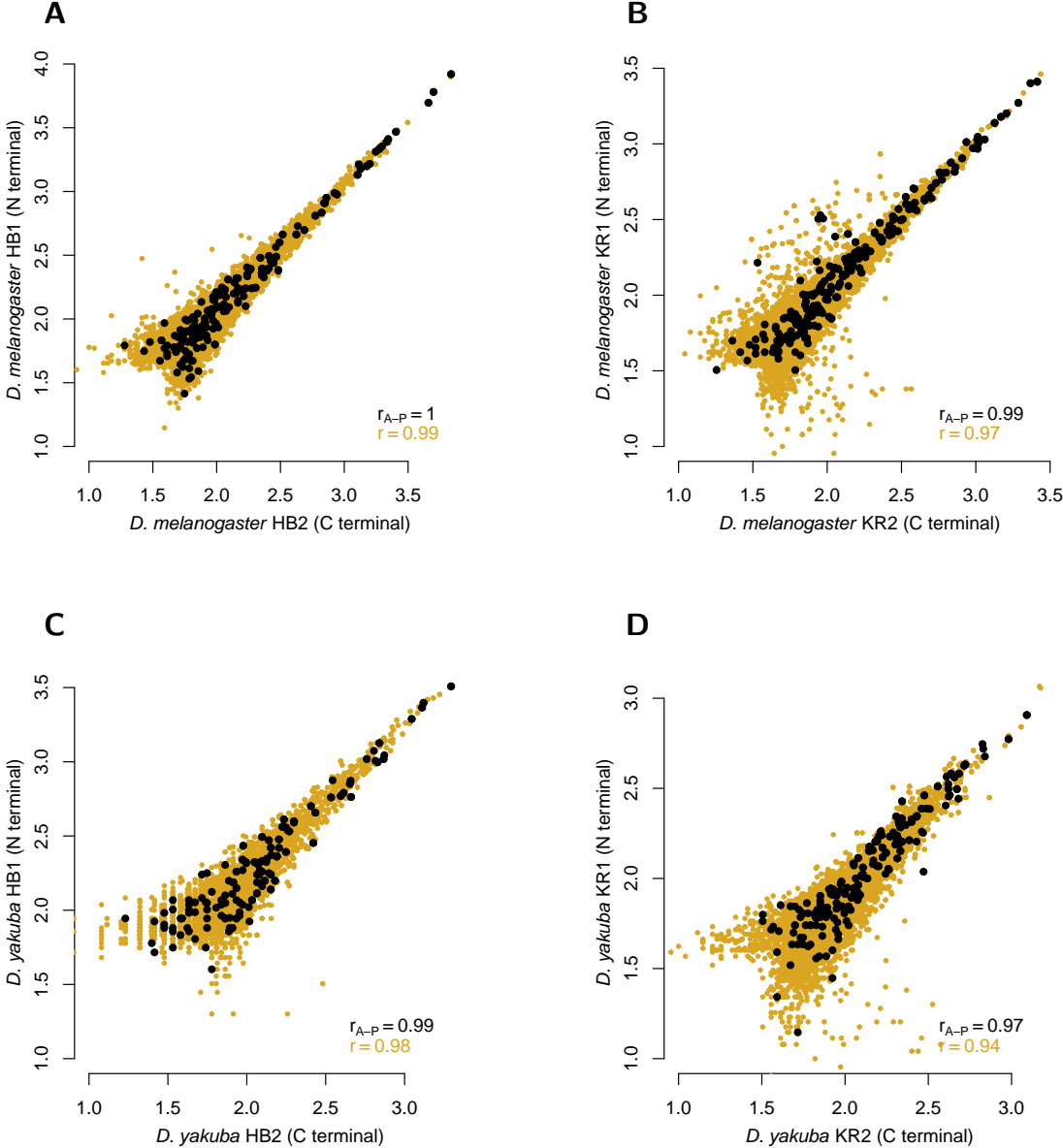

Supplement: Figure S1 — Correlation between binding levels for peaks called for distinct antibodies (A) HB in D. melanogaster, (B) KR in D. melanogaster, (C) HB in D. yakuba, and (D) KR in D. yakuba. Correlations rounded to two significant digits. (1.75 MB PDF) [file pbio.1000343.s001.pdf]

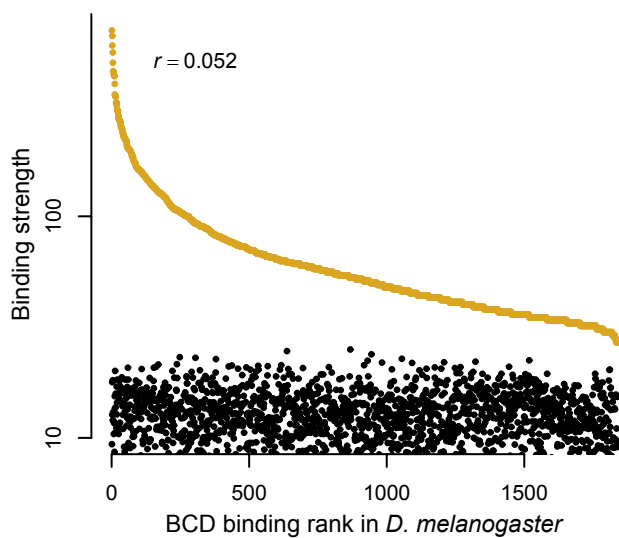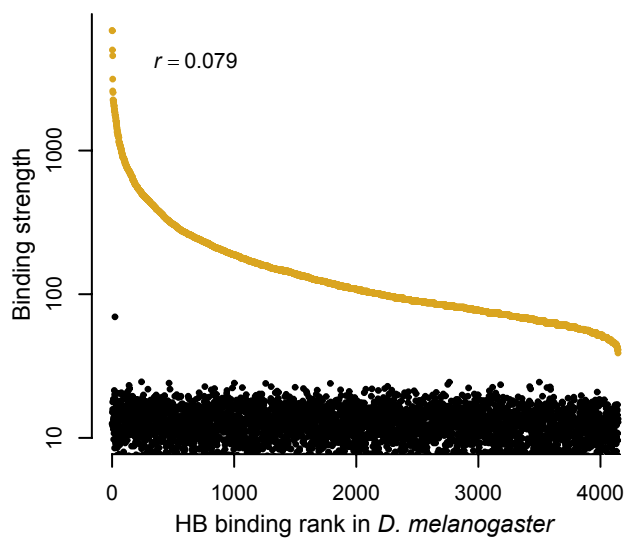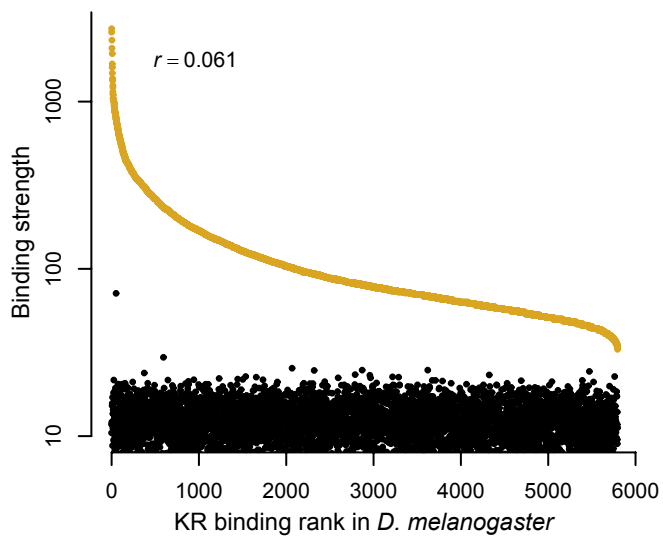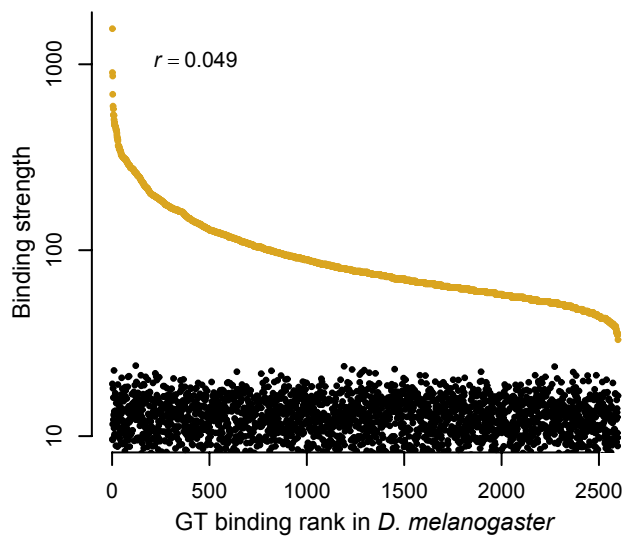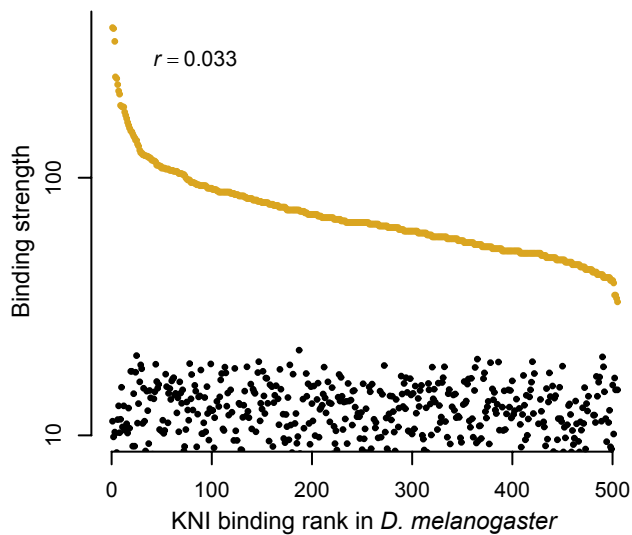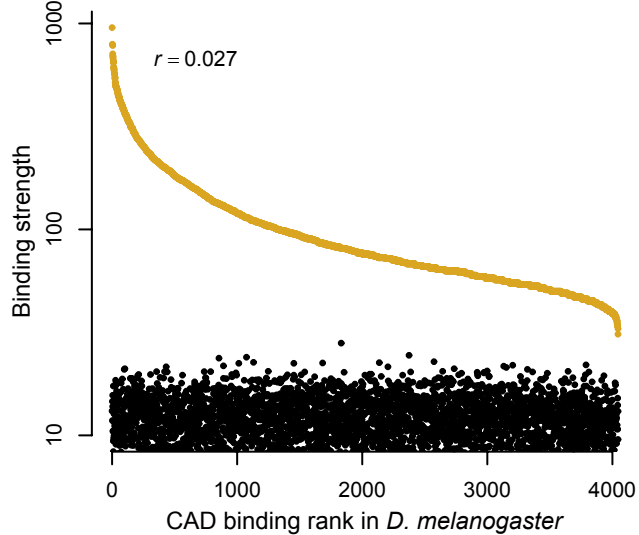

Supplement: Figure S2 — Correlations between Input and ChIP signals for D. melanogaster. Binding strength (goldenrod) and Input signal (black) for each peak called in D. melanogaster. (2.16 MB PDF) [file pbio.1000343.s002.pdf]

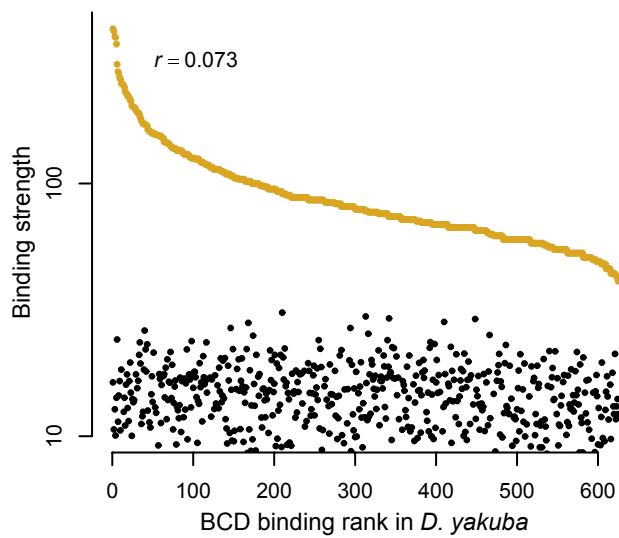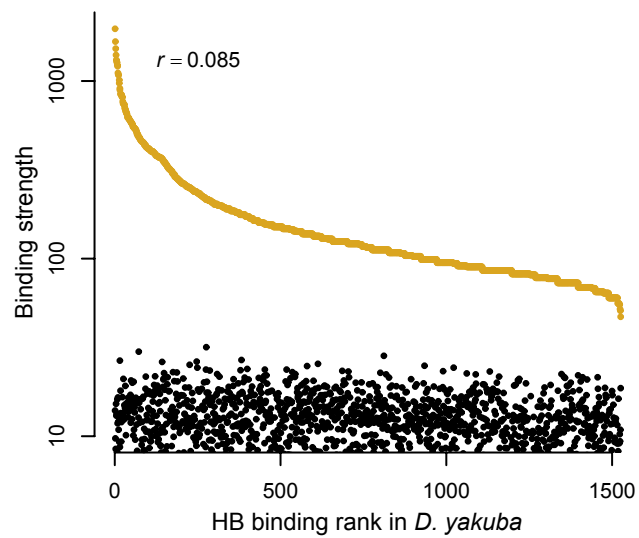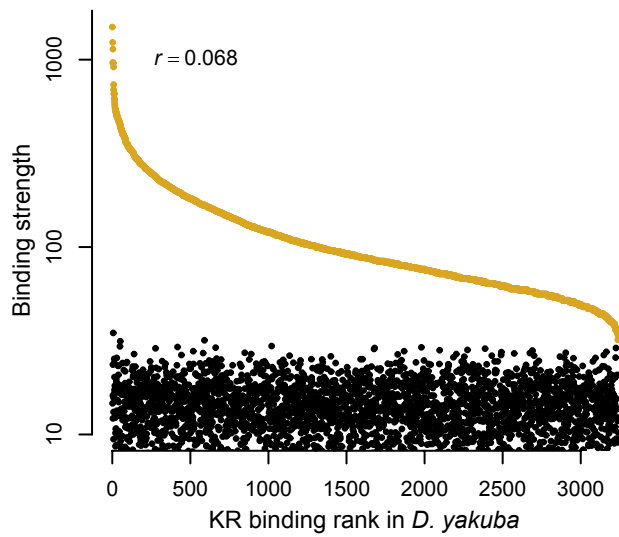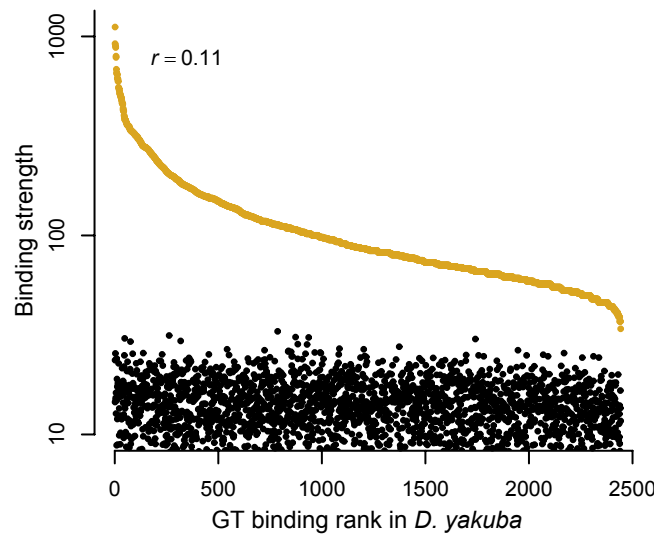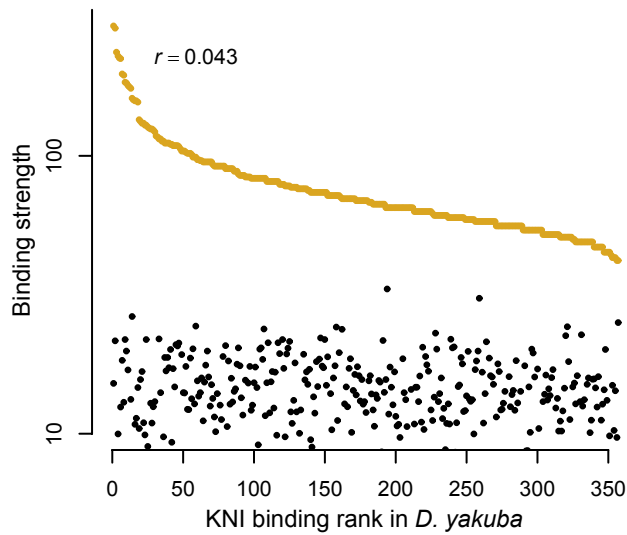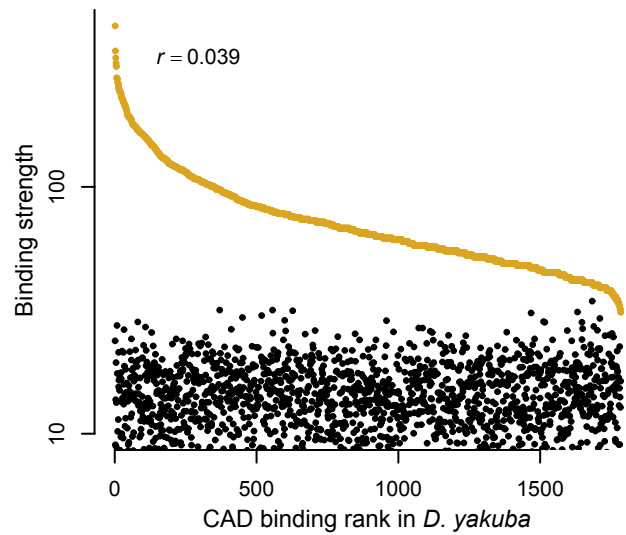

Supplement: Figure S3 — Correlations between Input and ChIP signals for D. yakuba. Binding strength (goldenrod) and Input signal (black) for each peak called in D. yakuba. (1.14 MB PDF) [file pbio.1000343.s003.pdf]

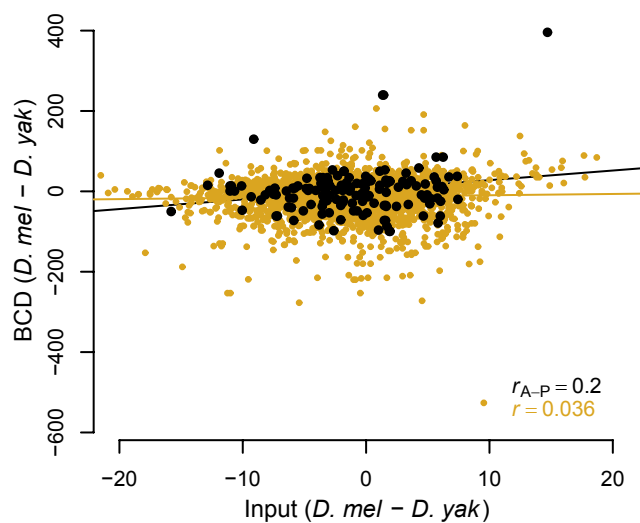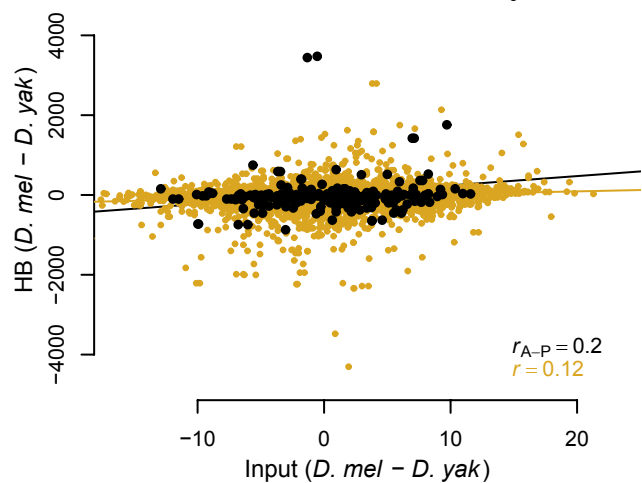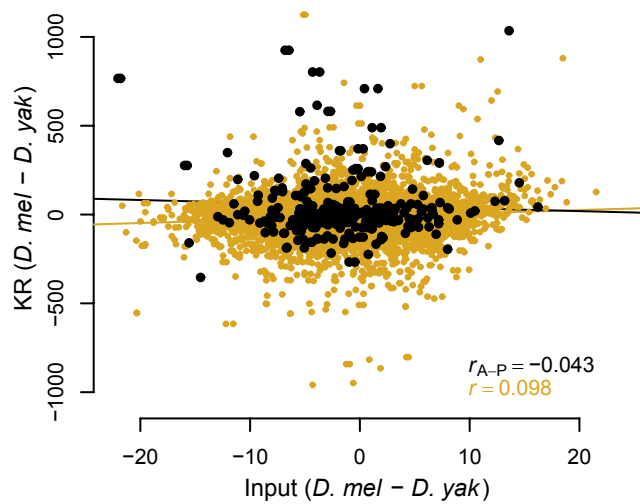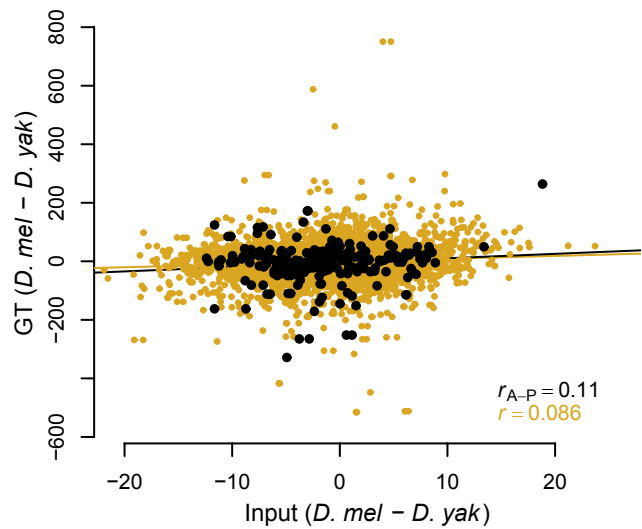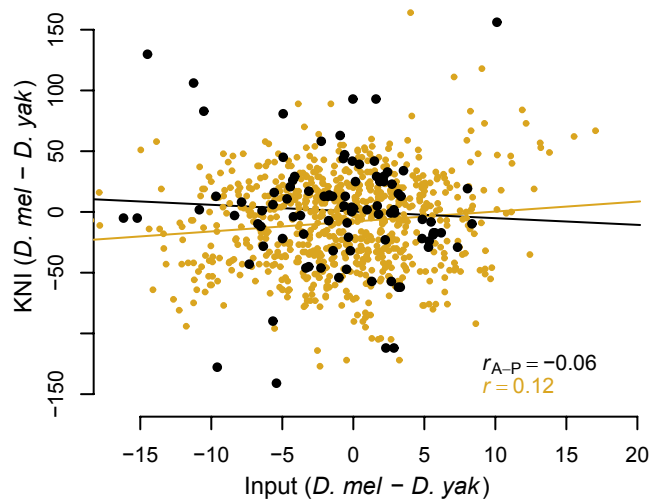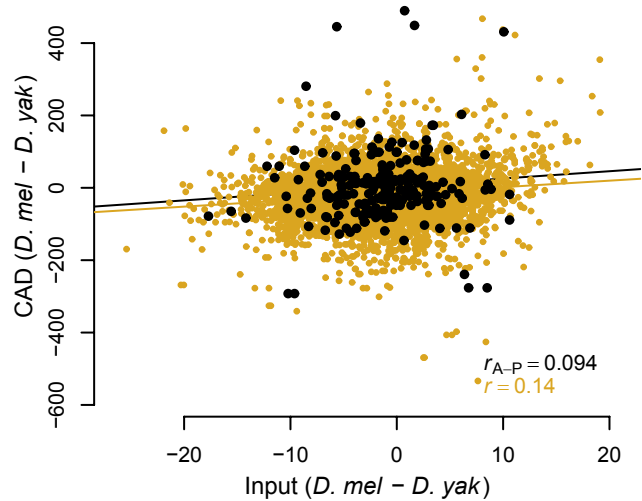

Supplement: Figure S4 — Correlations between divergence in Input and ChIP signals. Divergence in binding strength and Input signal for peaks called in either species. (1.65 MB PDF) [file pbio.1000343.s004.pdf]

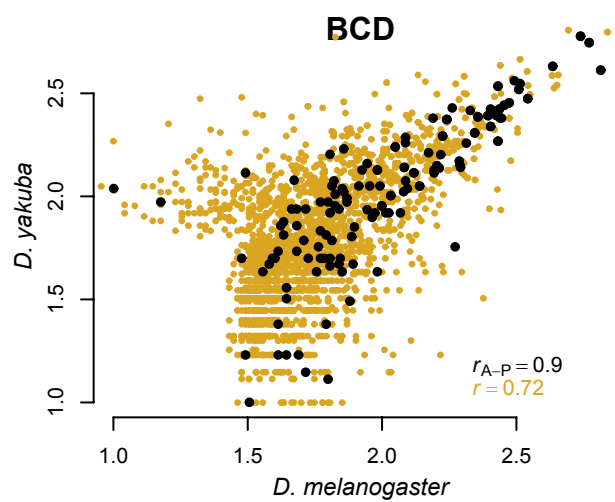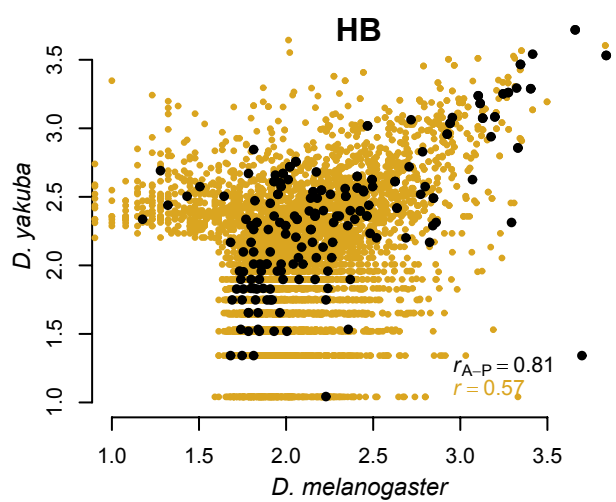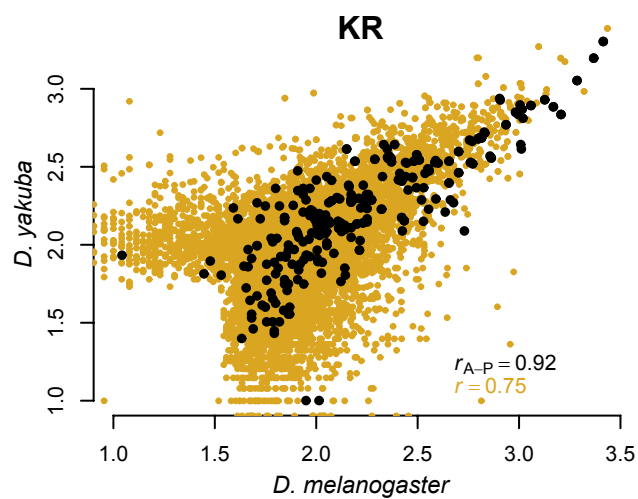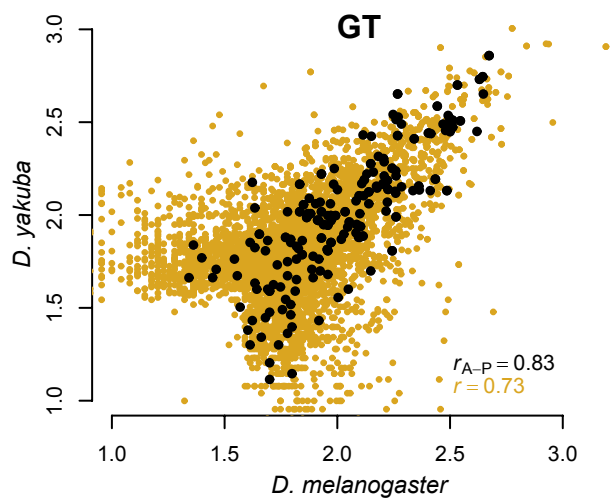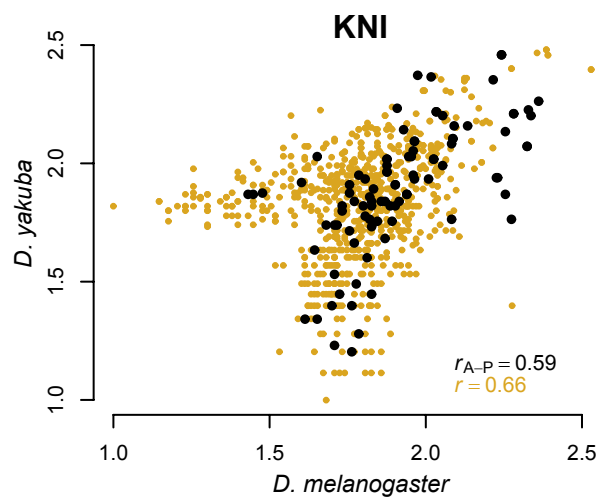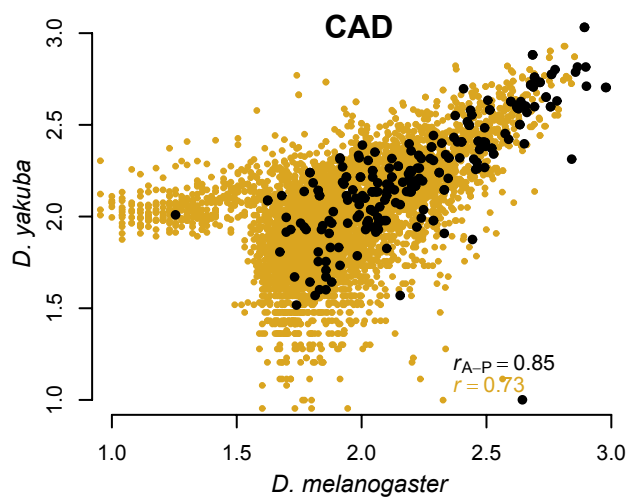

Supplement: Figure S5 — Comparison of binding between D. melanogaster and D. yakuba. Scatterplots of binding strengths at peaks called in either species for each factor. The x- and y-axes show the logarithms of the binding strengths in D. melanogaster and D. yakuba. (1.63 MB PDF) [file pbio.1000343.s005.pdf]

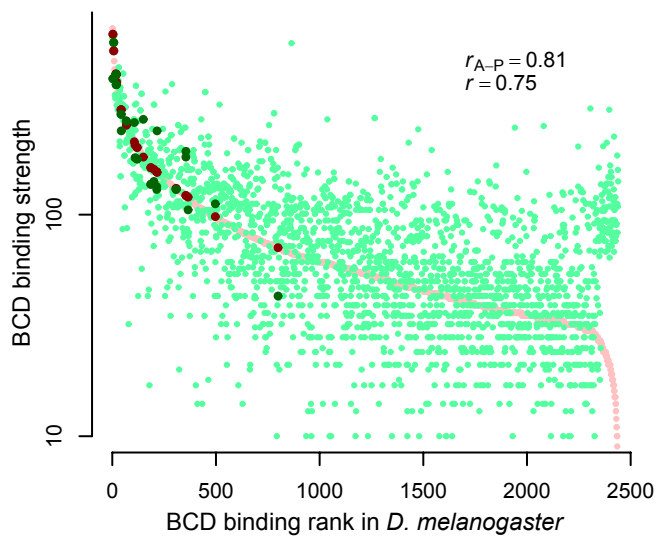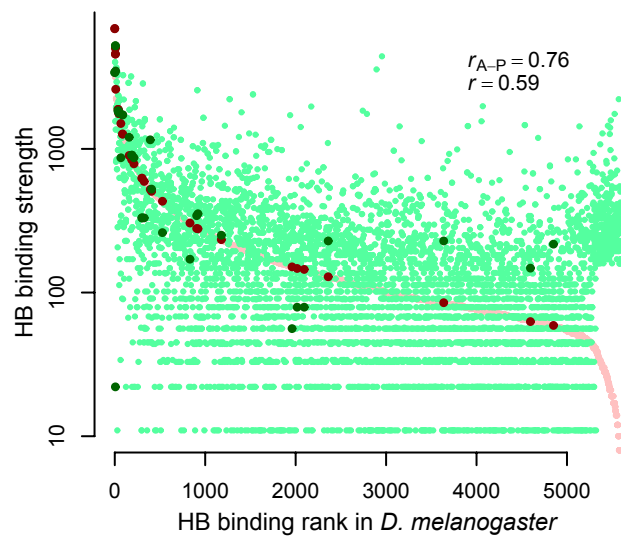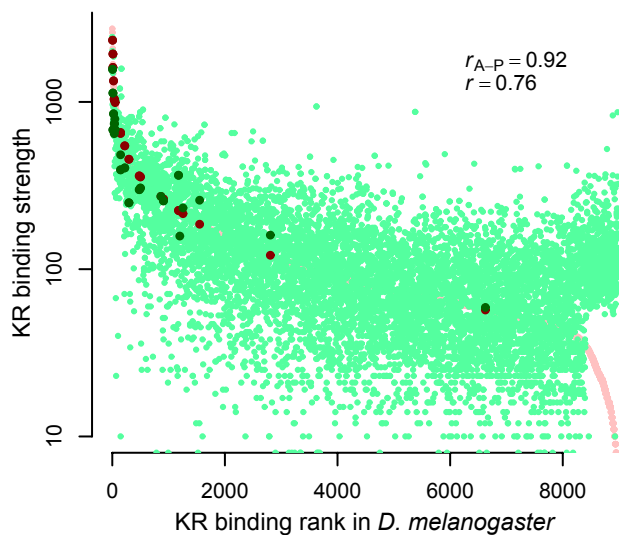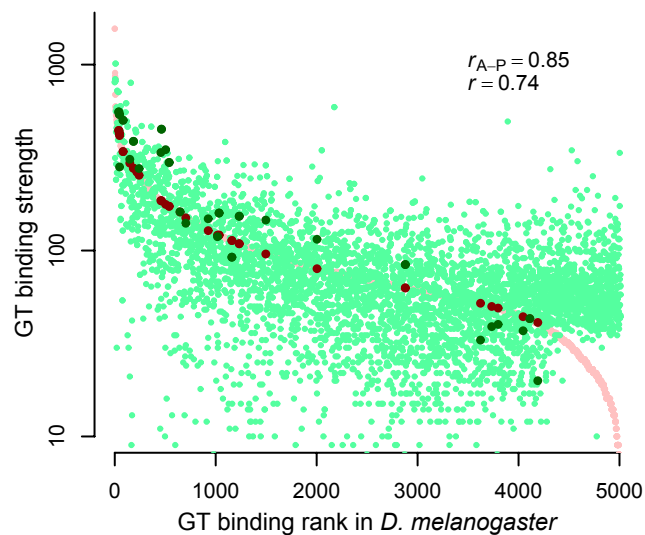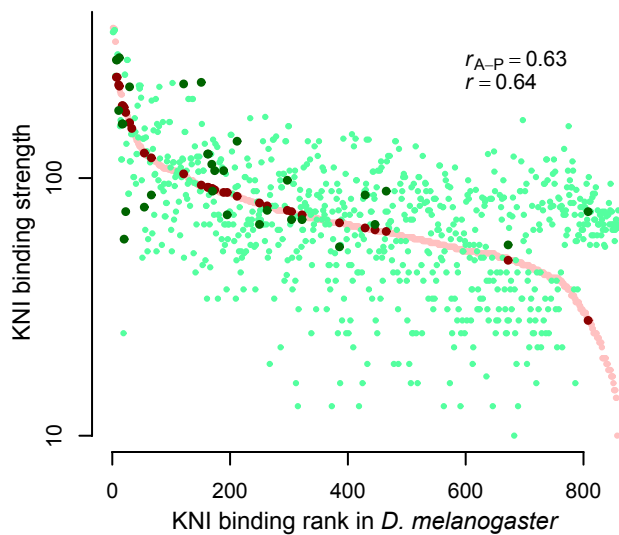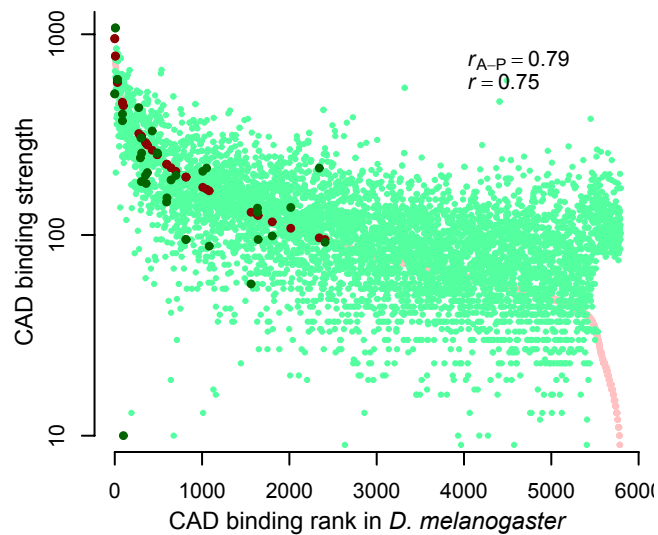

Supplement: Figure S6 — Quantitative variation in binding between species. Comparison of binding levels in D. melanogaster and D. yakuba for all identified bound regions. For each peak called in either species, we plotted the corresponding binding strengths in red for D. melanogaster and green for D. yakuba; dark colors indicate known cis-regulatory modules (compare with Figure 2, where dark colors indicate peaks near genes regulated by A-P factors). Peaks are ordered left-to-right on the x-axis according to their binding ranks in D. melanogaster and binding strengths in both genomes are plotted in log scale on the y-axis (binding units are arbitrary). (3.26 MB PDF) [file pbio.1000343.s006.pdf]

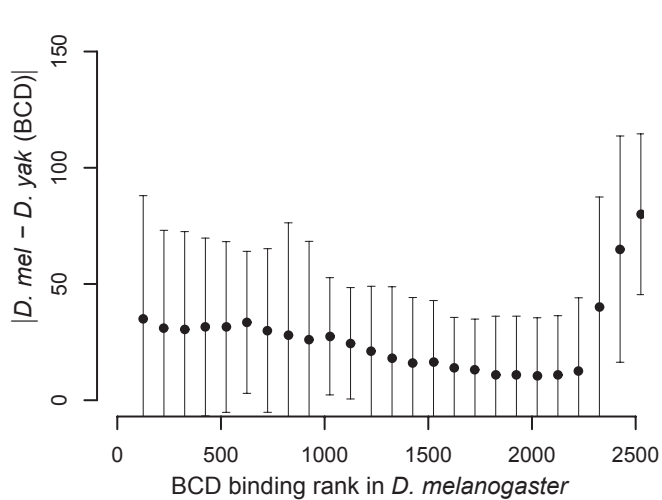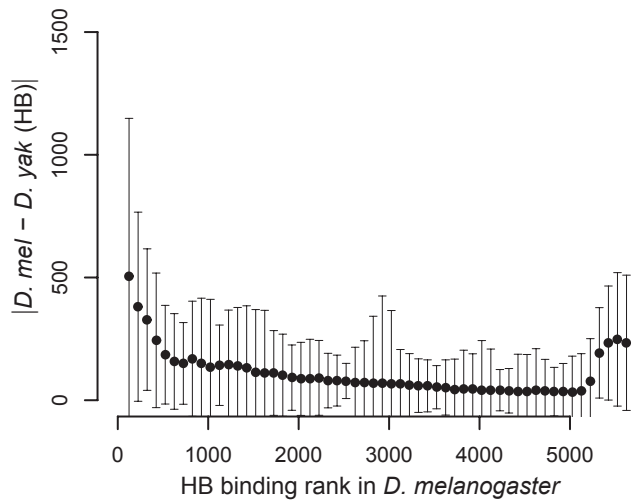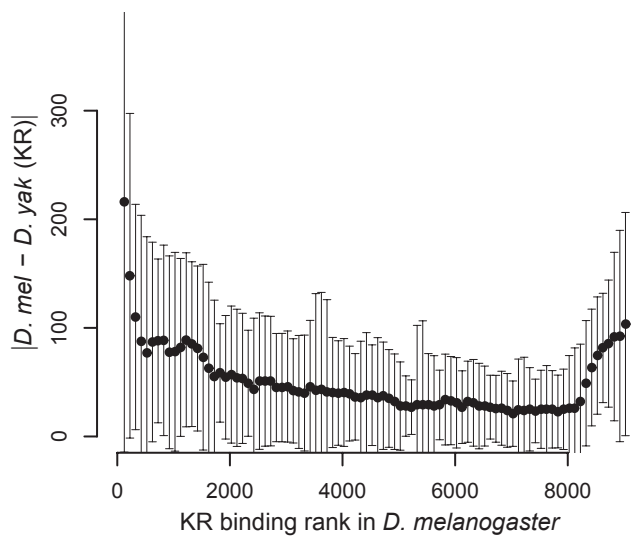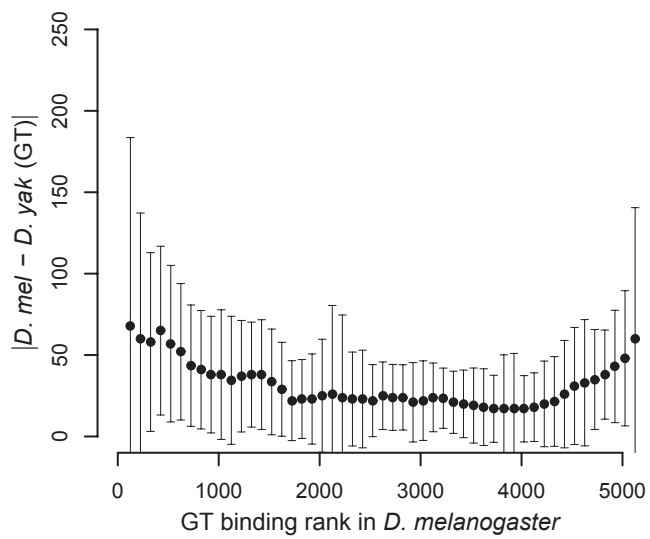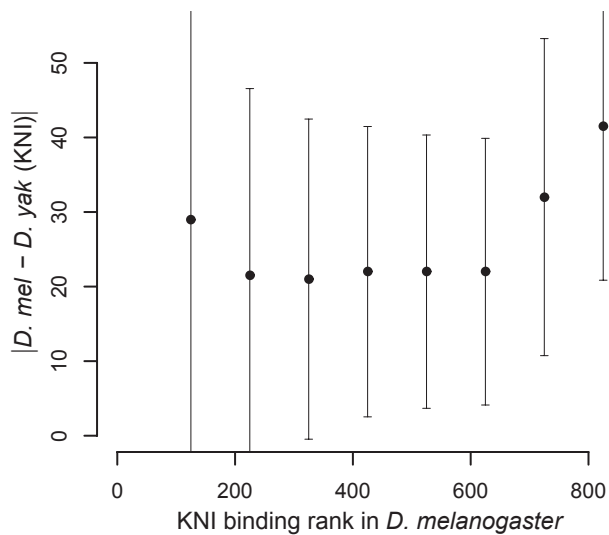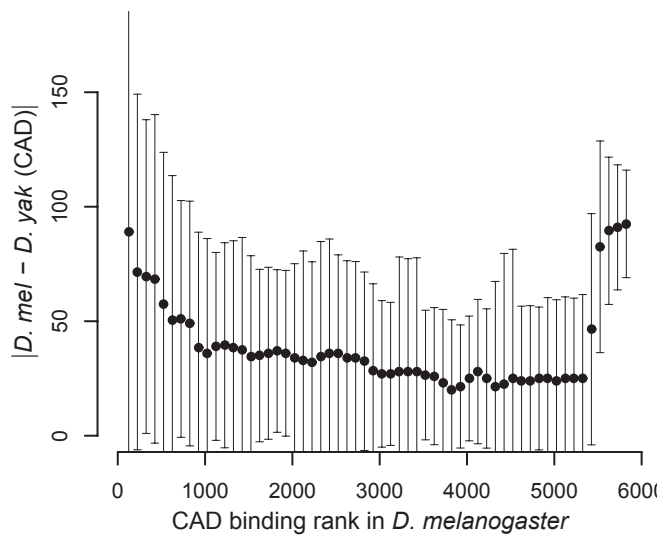

Supplement: Figure S7 — Absolute binding divergence as a function of binding strength. Absolute binding divergence, computed as |D. melanogaster − D. yakuba|, for overlapping cohorts of 250 peaks called in either species. The error bars indicate the standard deviations of each cohort. As with Figure S7, the rising tails are due to peaks called in D. yakuba with little or no binding in D. melanogaster. (1.64 MB PDF) [file pbio.1000343.s007.pdf]

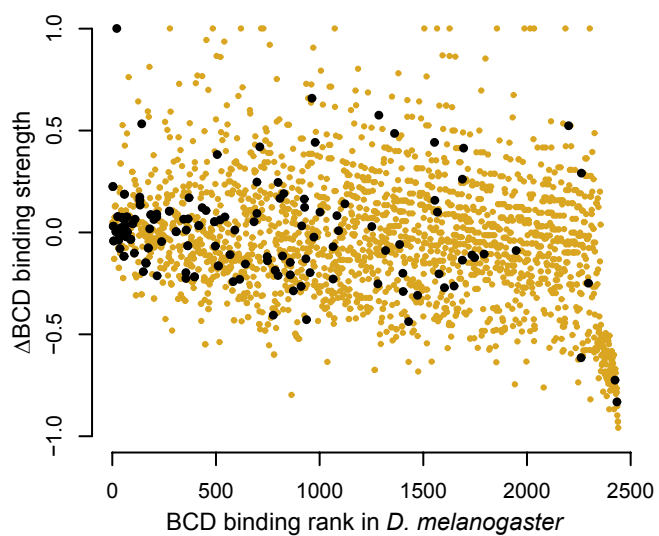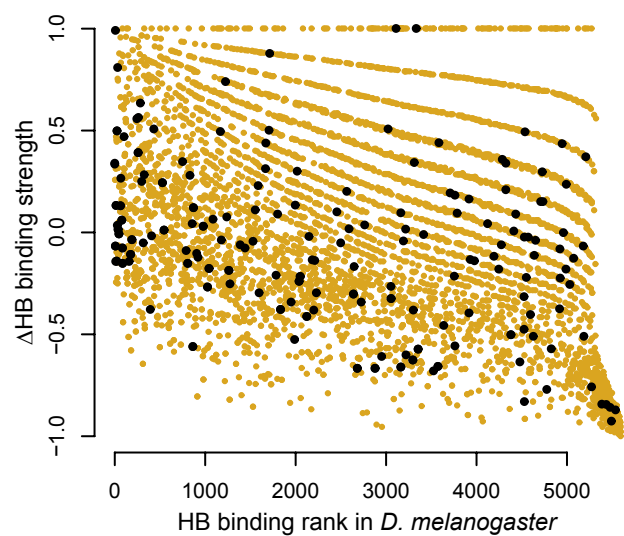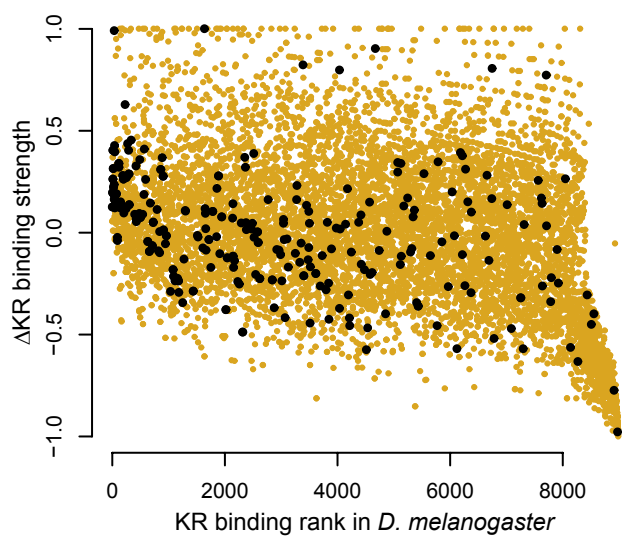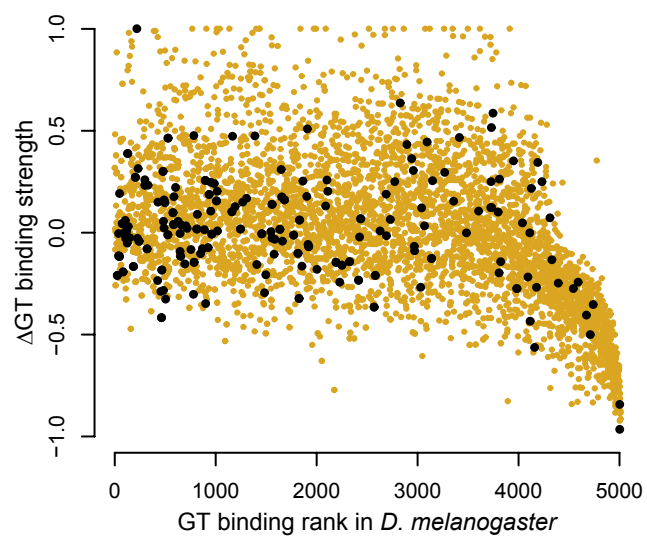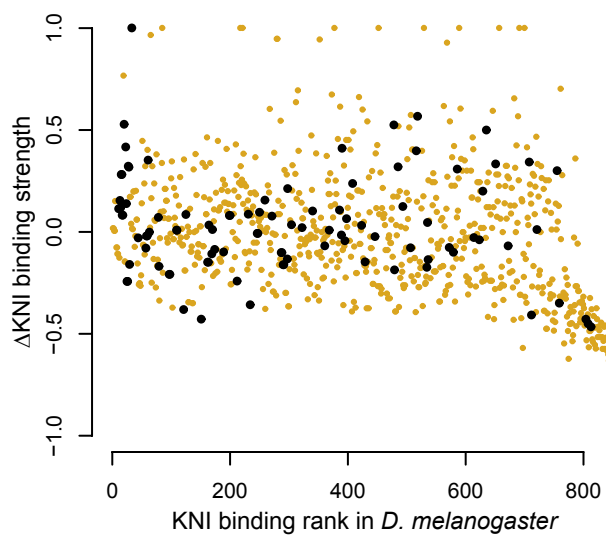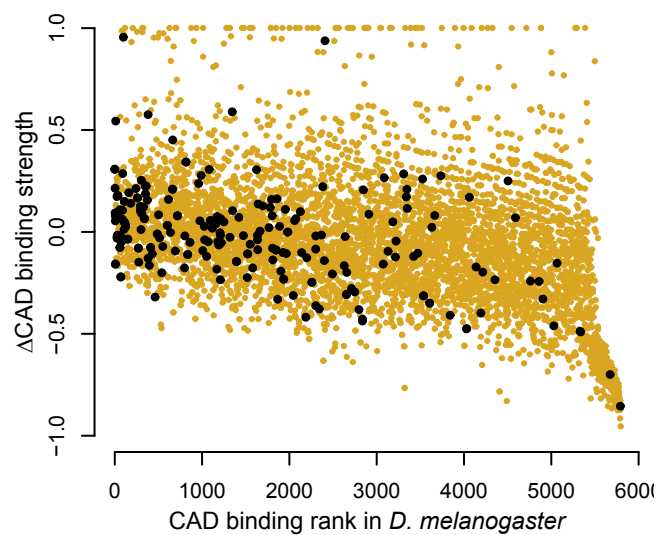

Supplement: Figure S8 — Fractional binding divergence for all peaks. Fractional binding divergence, computed as (D. melanogaster − D. yakuba) / (D. melanogaster + D. yakuba), for peaks called in either species. The downward trend of the datapoints from left to right is due to our comparative normalization procedure, which is based only on the ∼100 most highly bound regions near genes regulated by the A-P factors, and the ordering of the x-axis by binding rank in D. melanogaster. Peaks that are highly bound in D. yakuba but not in D. melanogaster tend to be placed on the right-hand side of the plot (since they are of low rank in D. melanogaster), and furthermore frequently have a negative fractional binding divergence since they are highly bound in D. yakuba but not in D. melanogaster. Similarly, the tails to the right correspond to peaks called in D. yakuba with little or no binding in D. melanogaster. Fractional binding divergence is similar for both highly bound and weakly bound regions. Figure 3 is similar to this figure, but shows median fractional binding divergence for cohorts of peaks rather than for every peak. (0.04 MB PDF) [file pbio.1000343.s008.pdf]

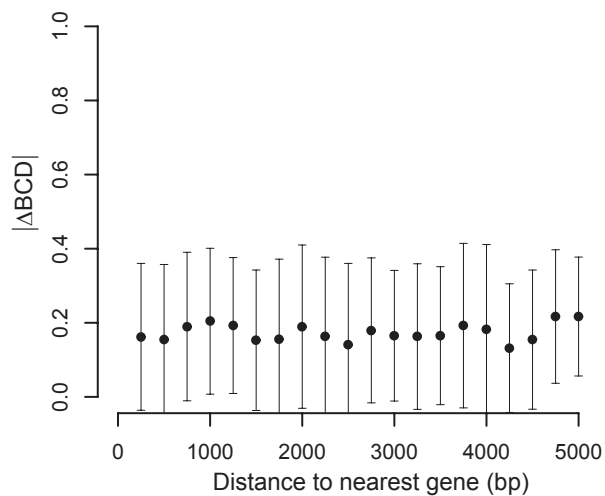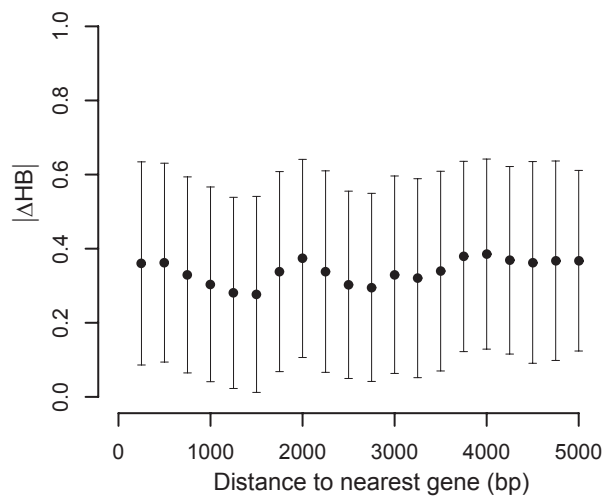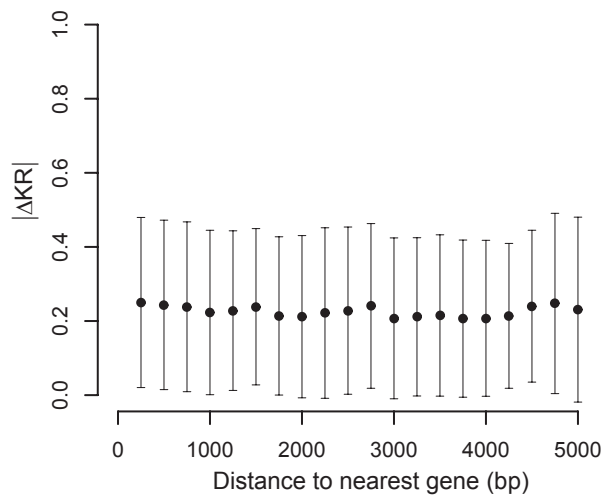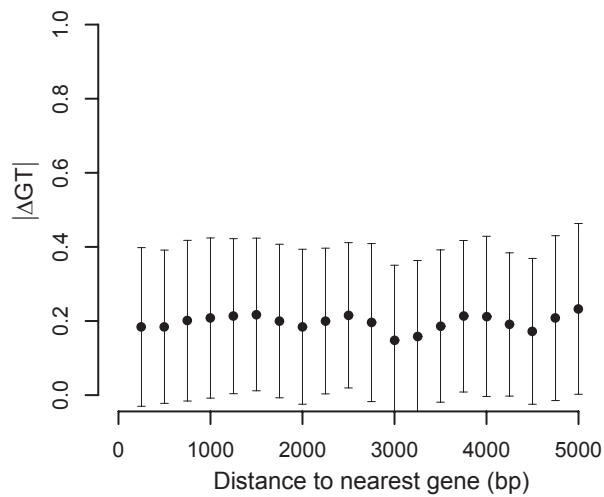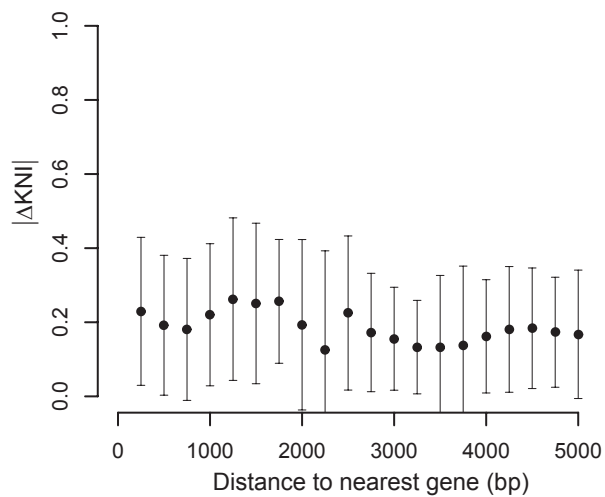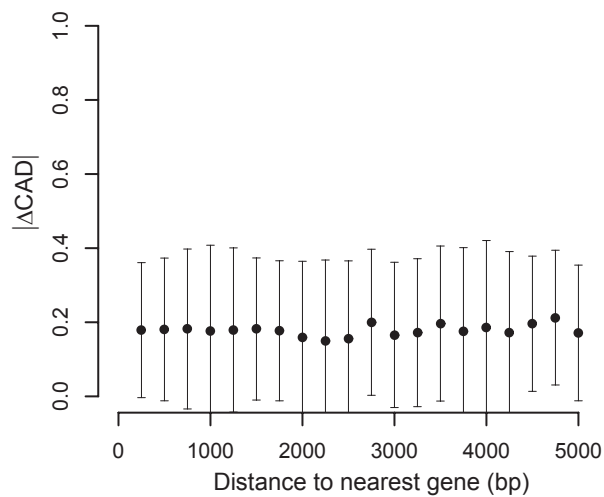

Supplement: Figure S9 — Relationship between fractional binding divergence and distance to nearest gene. (0.08 MB PDF) [file pbio.1000343.s009.pdf]

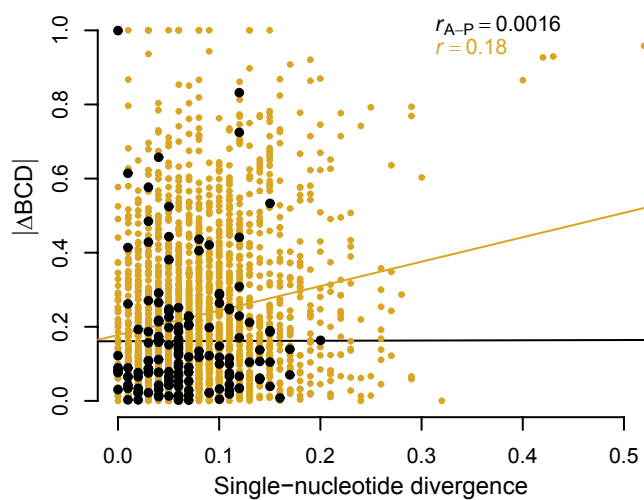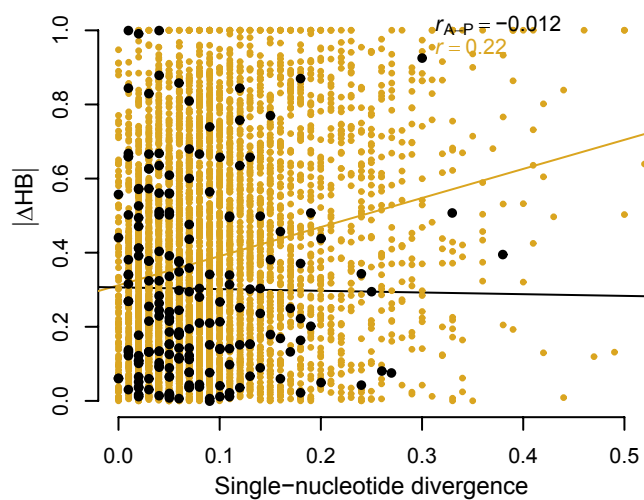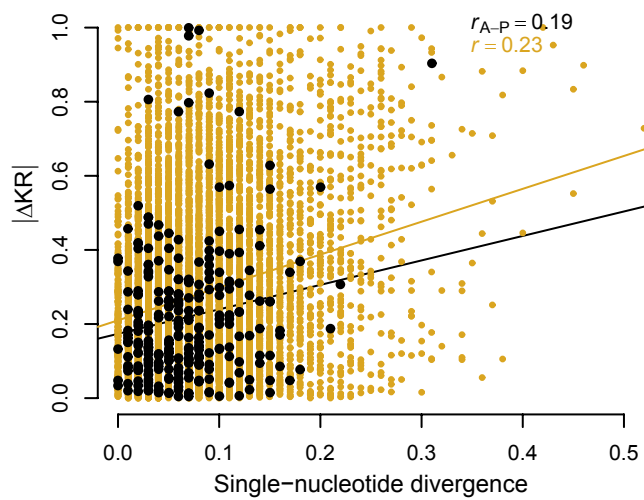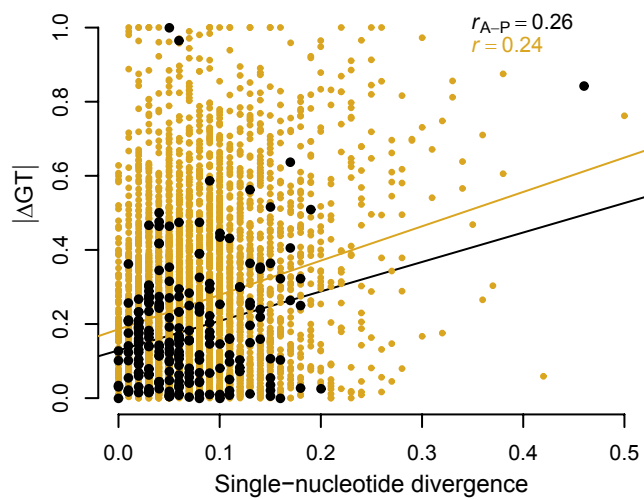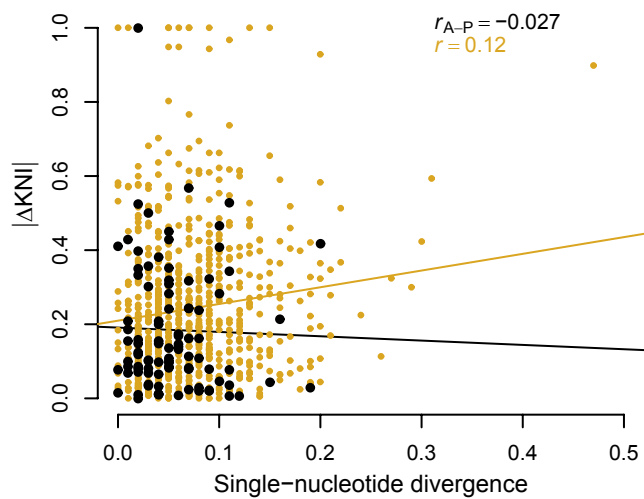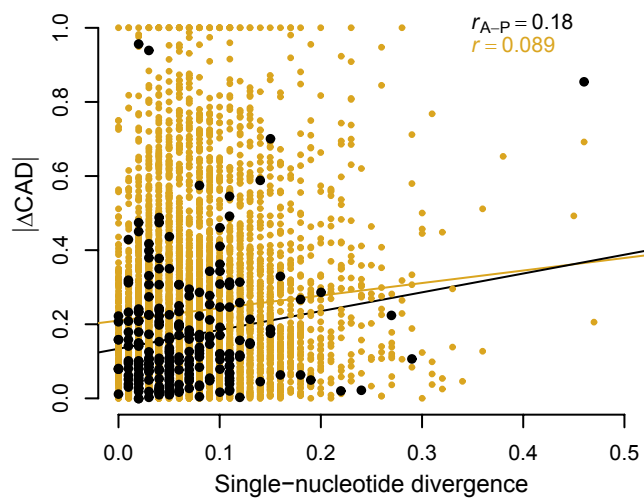

Supplement: Figure S10 — Correlation between single-nucleotide divergence and fractional binding divergence. Best-fit linear models shown. (1.64 MB PDF) [file pbio.1000343.s010.pdf]

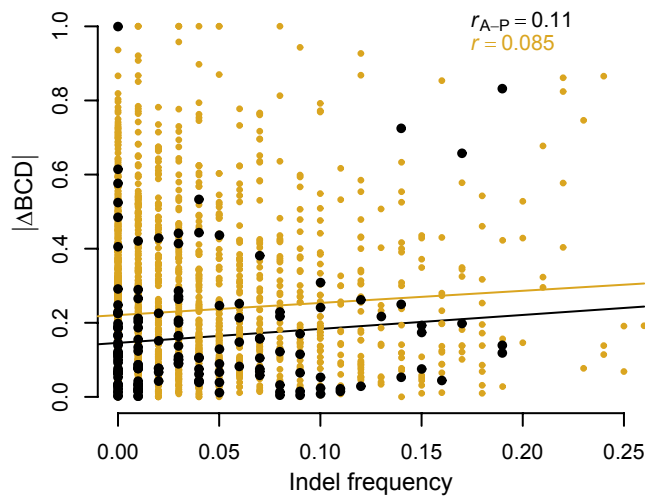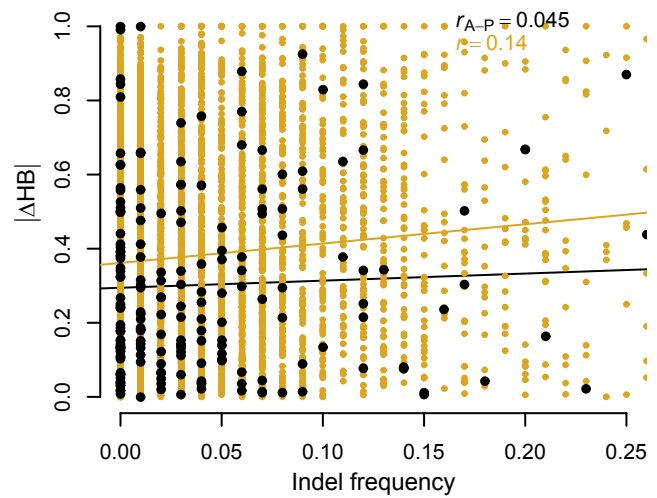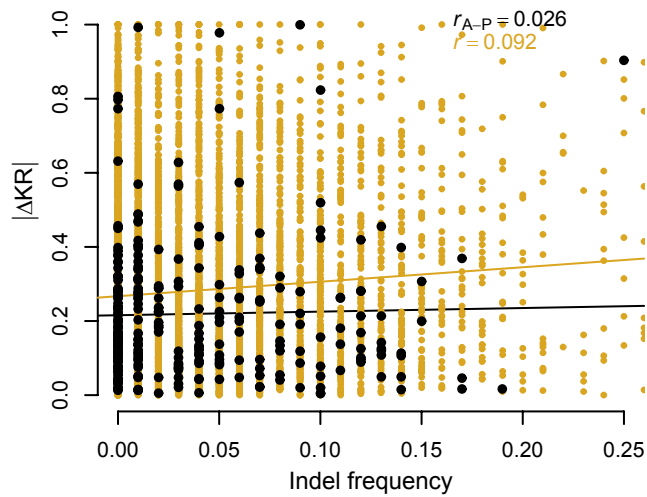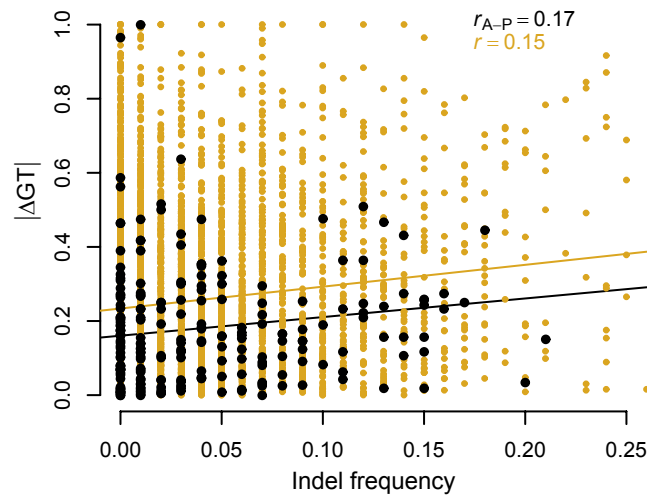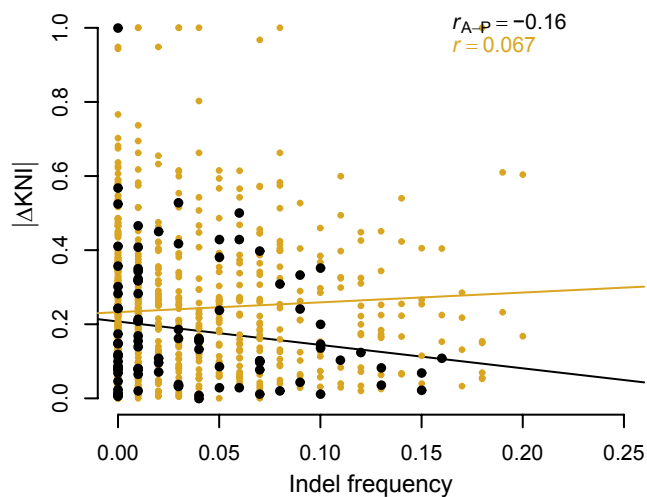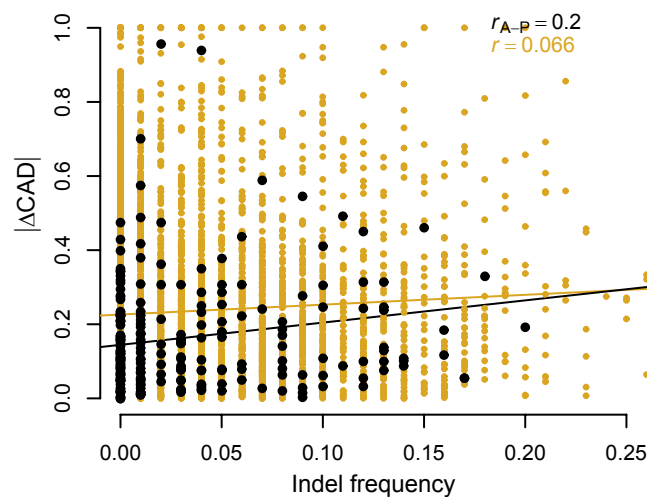

Supplement: Figure S11 — Correlation between insertion/deletion frequency and fractional binding divergence. Best-fit linear models shown. (1.63 MB PDF) [file pbio.1000343.s011.pdf]

Binding divergence of BCD

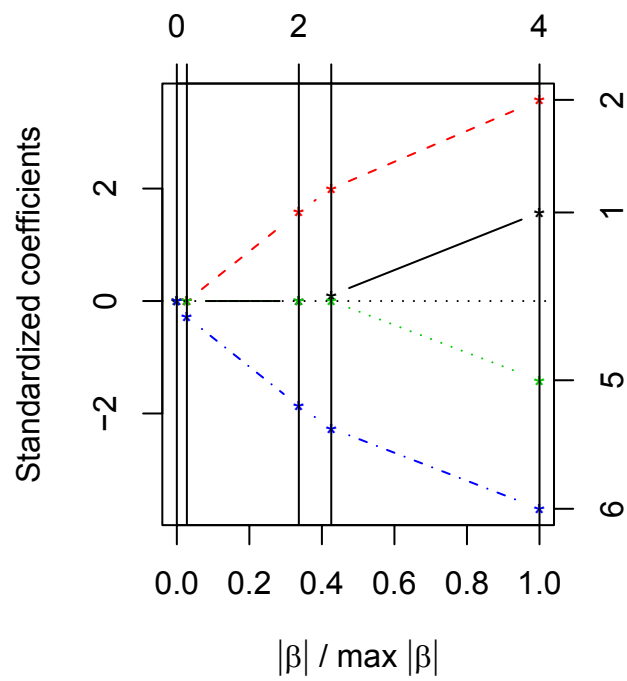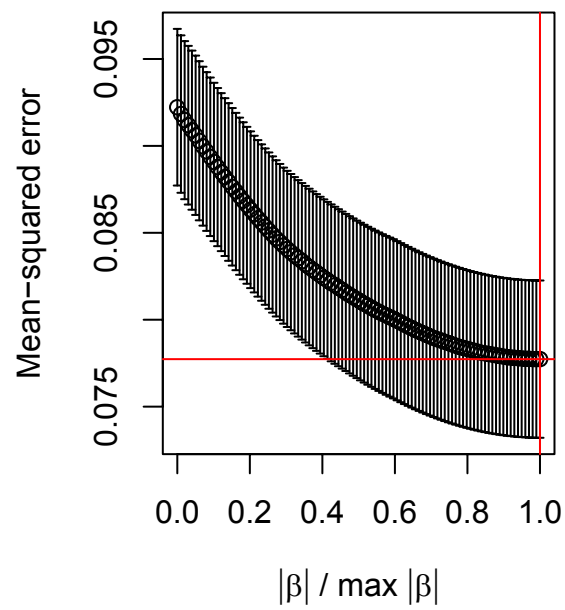

Supplement: Figure S12 — Linear model of BCD binding divergence. Linear model of binding divergence using divergence-driving words (6 bp) identified for BCD. Left panel shows the model coefficients for each word as a function of the lasso regularization parameter β; right panel shows the mean-squared prediction error associated with each value of β based on a 5-fold cross-validation procedure. The constant decrease in prediction error indicates that including more words in the linear model helps with prediction, suggesting that the DDWs that we identified do guide factor binding. Figure created by the lars package in R. (0.02 MB PDF) [file pbio.1000343.s012.pdf]

### Binding divergence of BCD

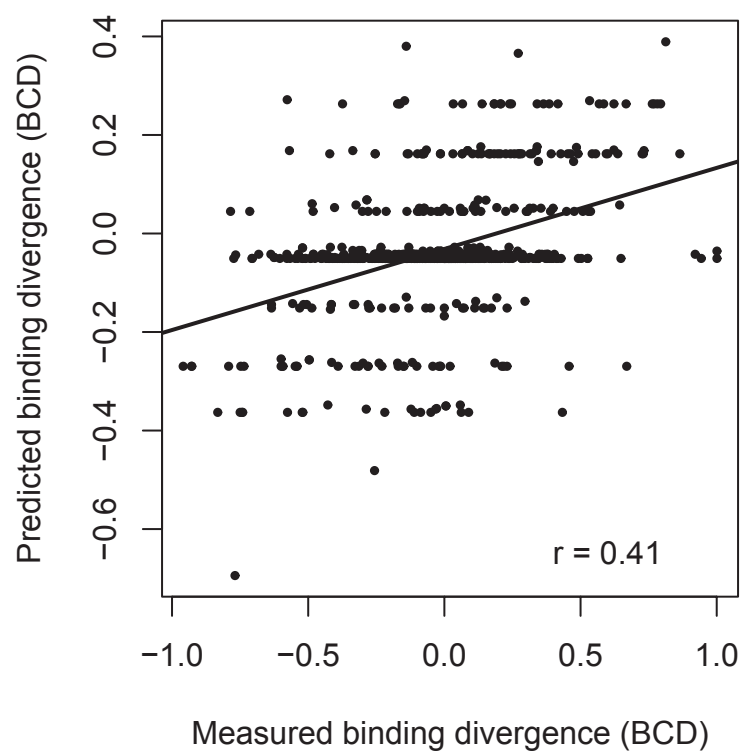

Supplement: Figure S13 — Accuracy of linear model predictions for BCD. Scatterplot of measured and predicted binding divergence for BCD. Predictions used the linear model illustrated in Figure S12, where model coefficients were chosen to minimize the cross-validation prediction error. (0.05 MB PDF) [file pbio.1000343.s013.pdf]

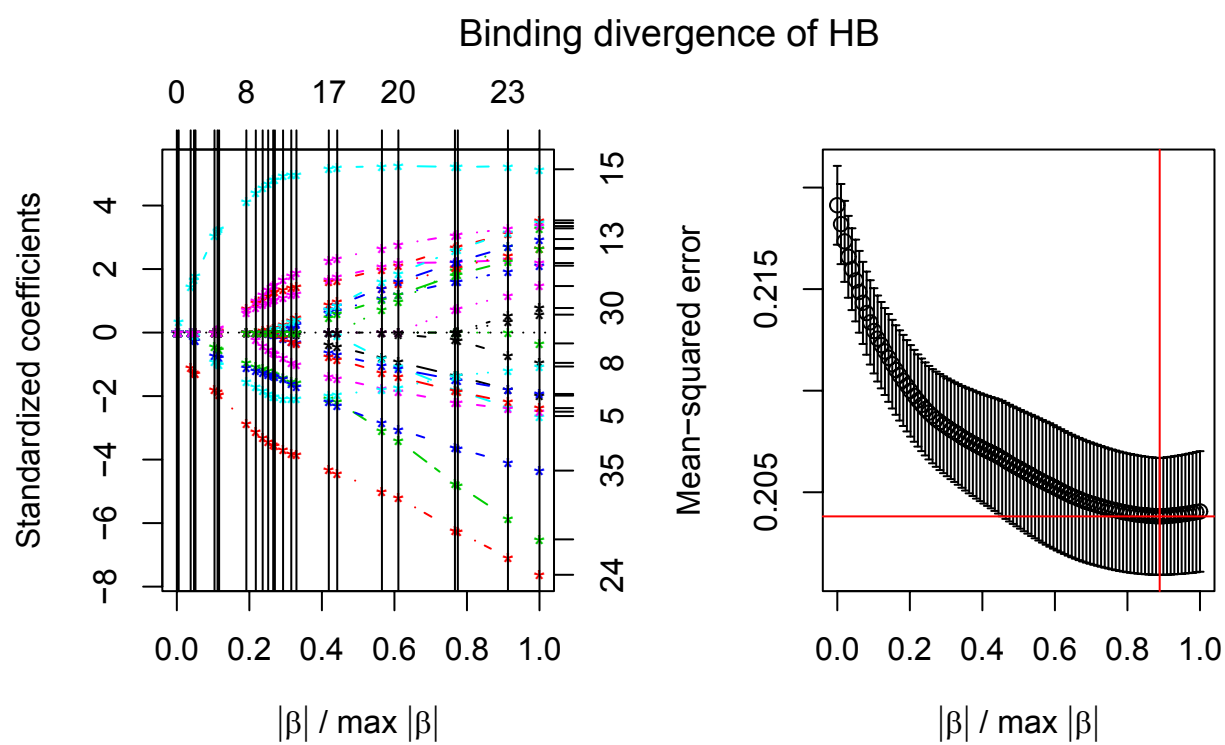

Supplement: Figure S14 — Linear model of HB binding divergence. Linear model of binding divergence using divergence-driving words (6 bp) identified for HB. Left panel shows the model coefficients for each word as a function of the lasso regularization parameter β; right panel shows the mean-squared prediction error associated with each value of β based on a 5-fold cross-validation procedure. The constant decrease in prediction error indicates that including more words in the linear model helps with prediction, suggesting that the DDWs that we identified do guide factor binding. Figure created by the lars package in R. (0.06 MB PDF) [file pbio.1000343.s014.pdf]

### Binding divergence of HB

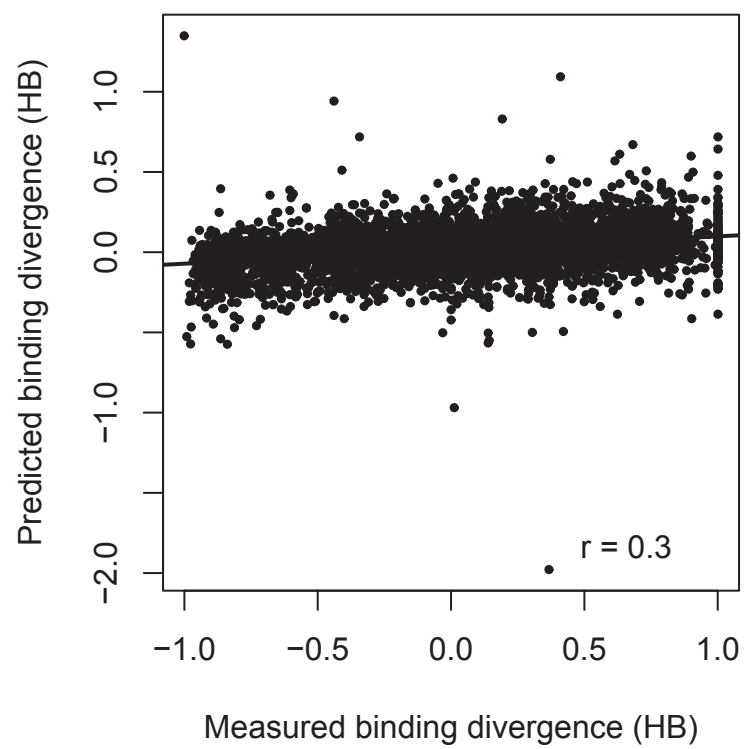

Supplement: Figure S15 — Accuracy of linear model predictions for HB. Scatterplot of measured and predicted binding divergence for HB. Predictions used the linear model illustrated in Figure S14, where model coefficients were chosen to minimize the cross-validation prediction error. (0.30 MB PDF) [file pbio.1000343.s015.pdf]

Binding divergence of KR

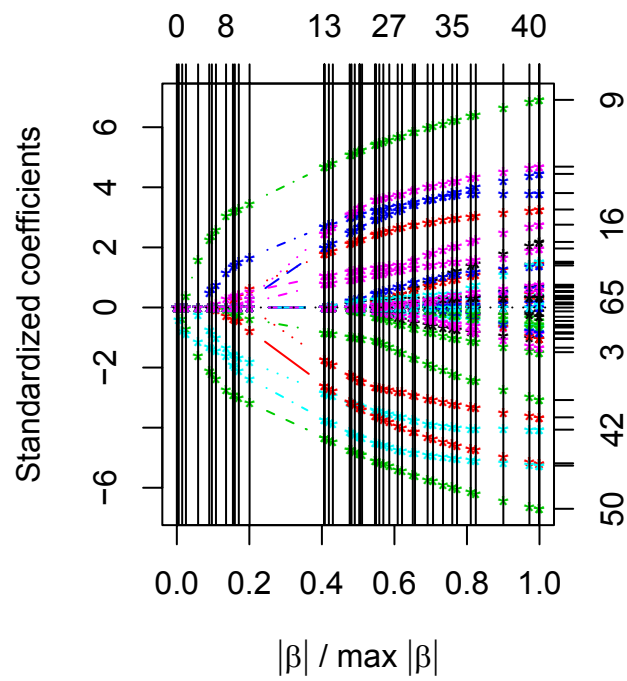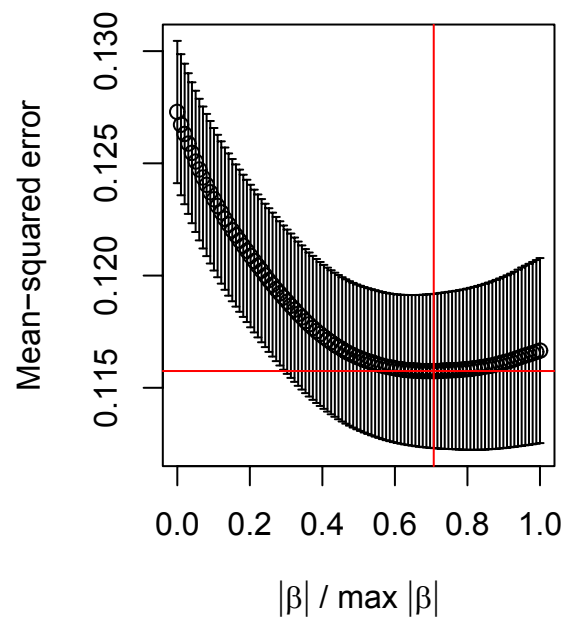

Supplement: Figure S16 — Linear model of KR binding divergence. Linear model of binding divergence using divergence-driving words (6 bp) identified for KR. Left panel shows the model coefficients for each word as a function of the lasso regularization parameter β; right panel shows the mean-squared prediction error associated with each value of β based on a 5-fold cross-validation procedure. The constant decrease in prediction error indicates that including more words in the linear model helps with prediction, suggesting that the DDWs that we identified do guide factor binding. Figure created by the lars package in R. (0.13 MB PDF) [file pbio.1000343.s016.pdf]

### Binding divergence of KR

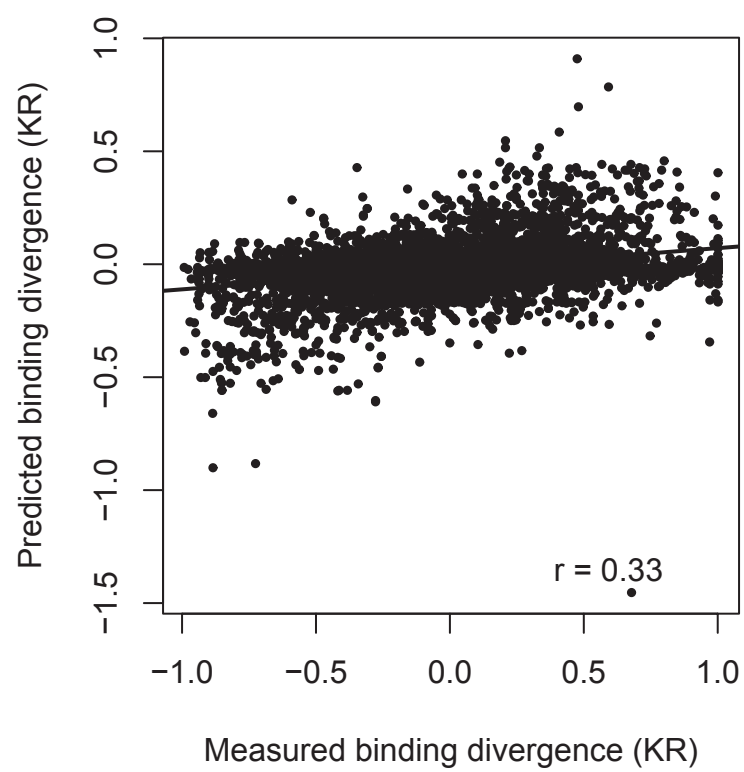

Supplement: Figure S17 — Accuracy of linear model predictions for KR. Scatterplot of measured and predicted binding divergence for KR. Predictions used the linear model illustrated in Figure S16, where model coefficients were chosen to minimize the cross-validation prediction error. (0.43 MB PDF) [file pbio.1000343.s017.pdf]

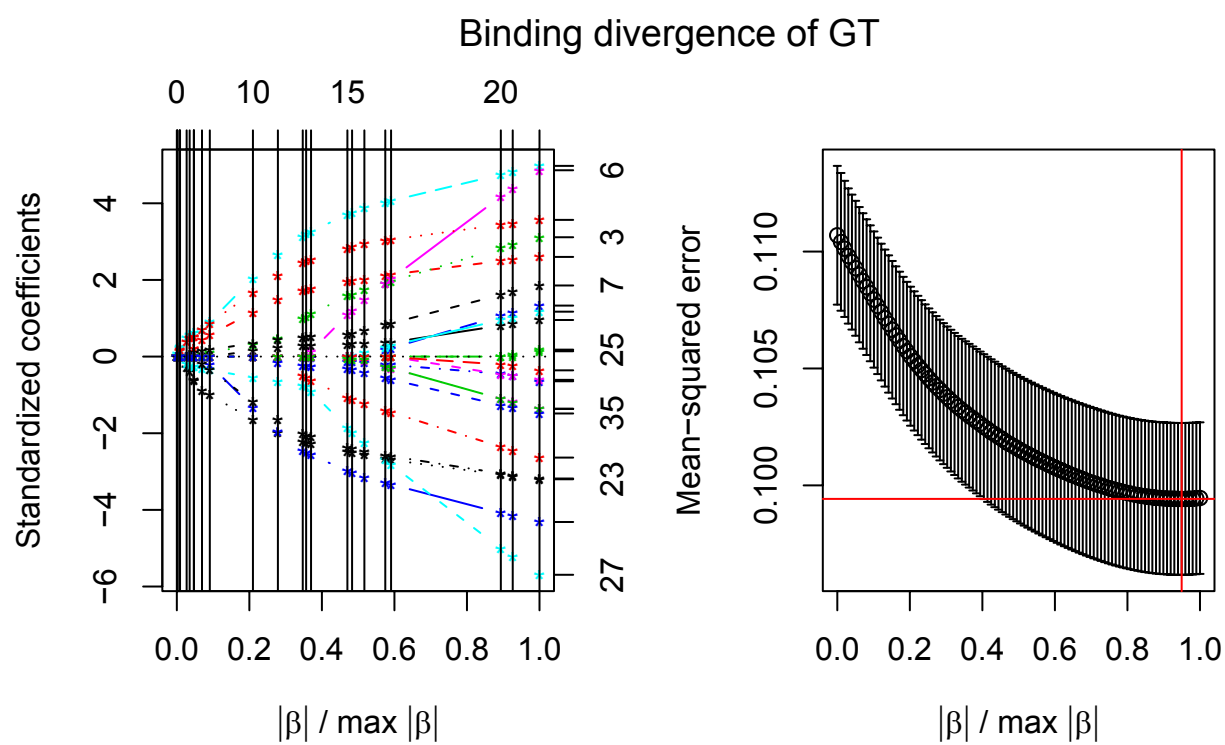

Supplement: Figure S18 — Linear model of GT binding divergence. Linear model of binding divergence using divergence-driving words (6 bp) identified for GT. Left panel shows the model coefficients for each word as a function of the lasso regularization parameter β; right panel shows the mean-squared prediction error associated with each value of β based on a 5-fold cross-validation procedure. The constant decrease in prediction error indicates that including more words in the linear model helps with prediction, suggesting that the DDWs that we identified do guide factor binding. Figure created by the lars package in R. (0.06 MB PDF) [file pbio.1000343.s018.pdf]

### Binding divergence of GT

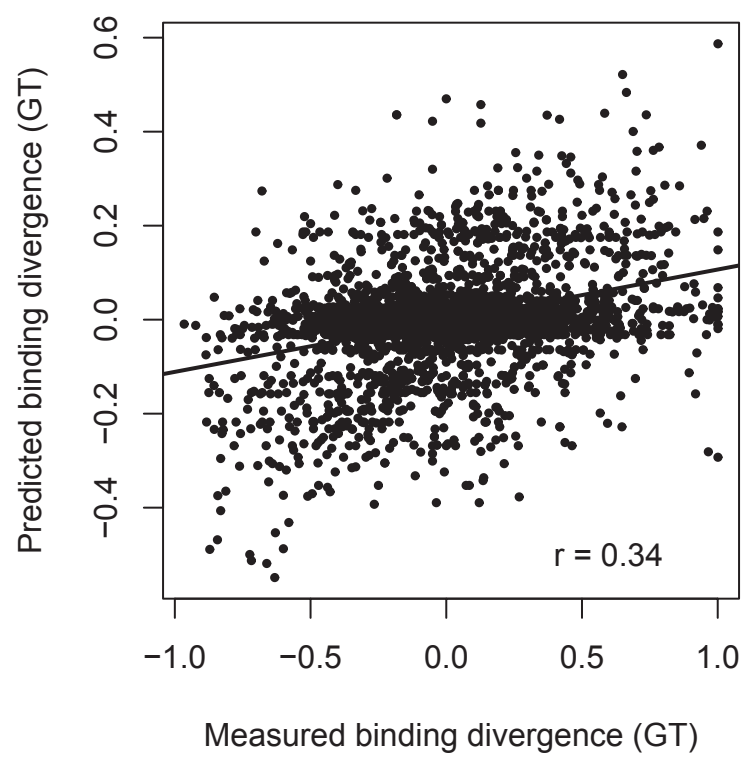

Supplement: Figure S19 — Accuracy of linear model predictions for GT. Scatterplot of measured and predicted binding divergence for GT. Predictions used the linear model illustrated in Figure S18, where model coefficients were chosen to minimize the cross-validation prediction error. (0.18 MB PDF) [file pbio.1000343.s019.pdf]

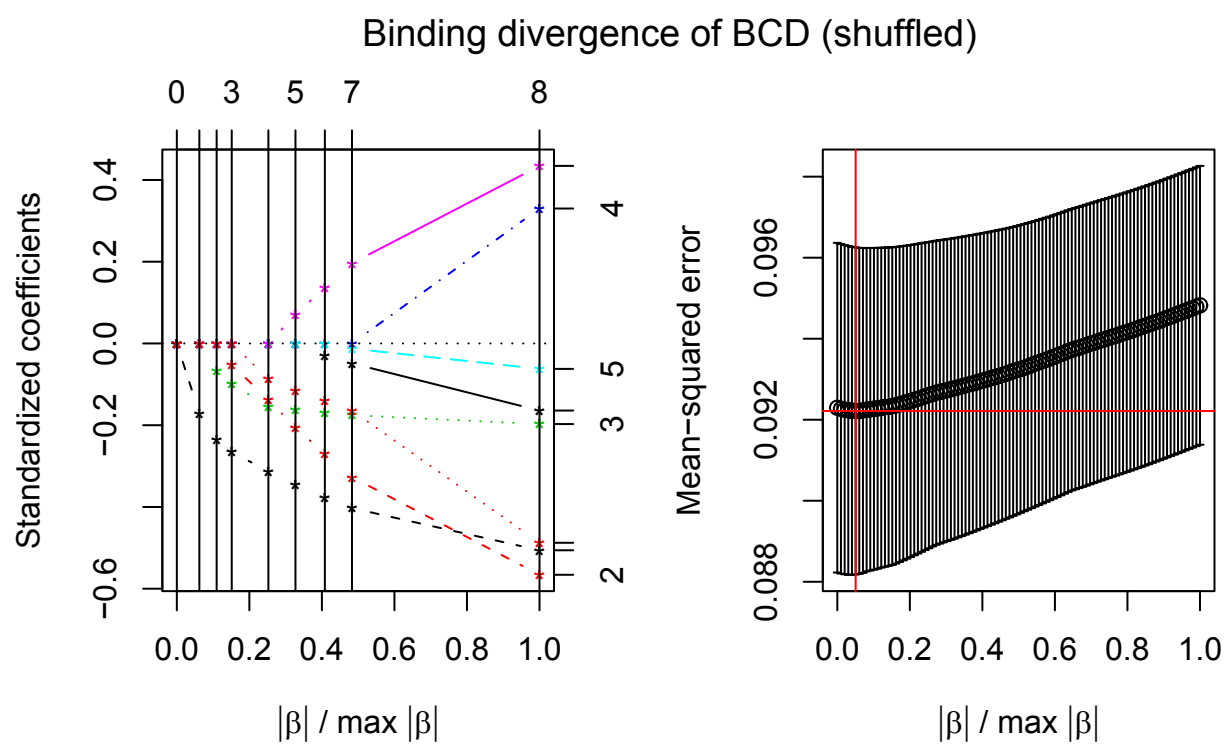

Supplement: Figure S20 — Linear model of BCD binding divergence (control with shuffled words). Linear model of binding divergence using shuffled versions of the divergence-driving words (6 bp) identified for BCD. The increase in prediction error indicates that including more shuffled words in the linear model does not help with prediction, suggesting that the shuffled DDWs that we identified do not guide factor binding. Compare with Figure S12. (0.03 MB PDF) [file pbio.1000343.s020.pdf]

### Binding divergence of BCD (shuffled)

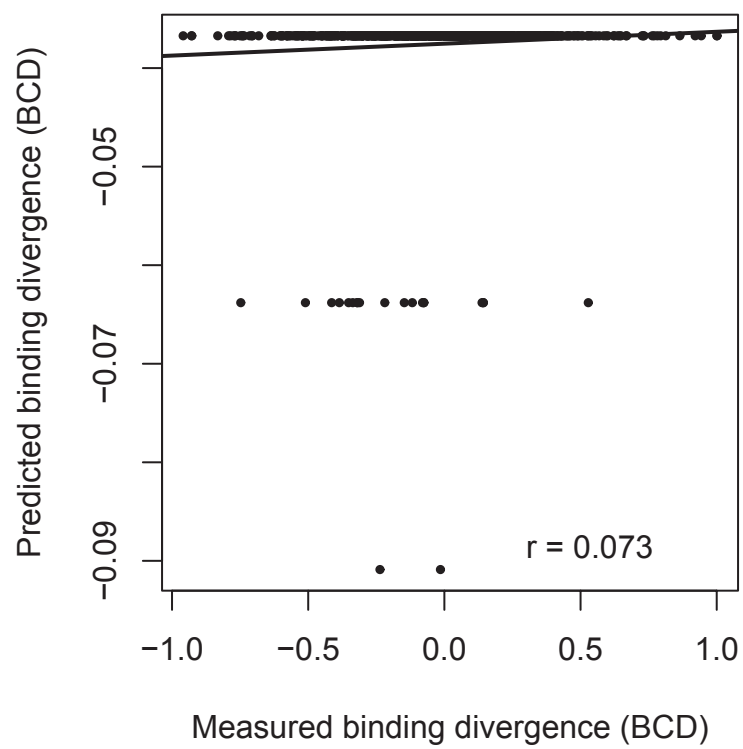

Supplement: Figure S21 — Accuracy of linear model predictions for BCD (control with shuffled words). Scatterplot of measured and predicted binding divergence for BCD. Predictions used the linear model illustrated in Figure S20, where model coefficients were chosen to minimize the cross-validation prediction error. Compare with Figure S13. (0.05 MB PDF) [file pbio.1000343.s021.pdf]

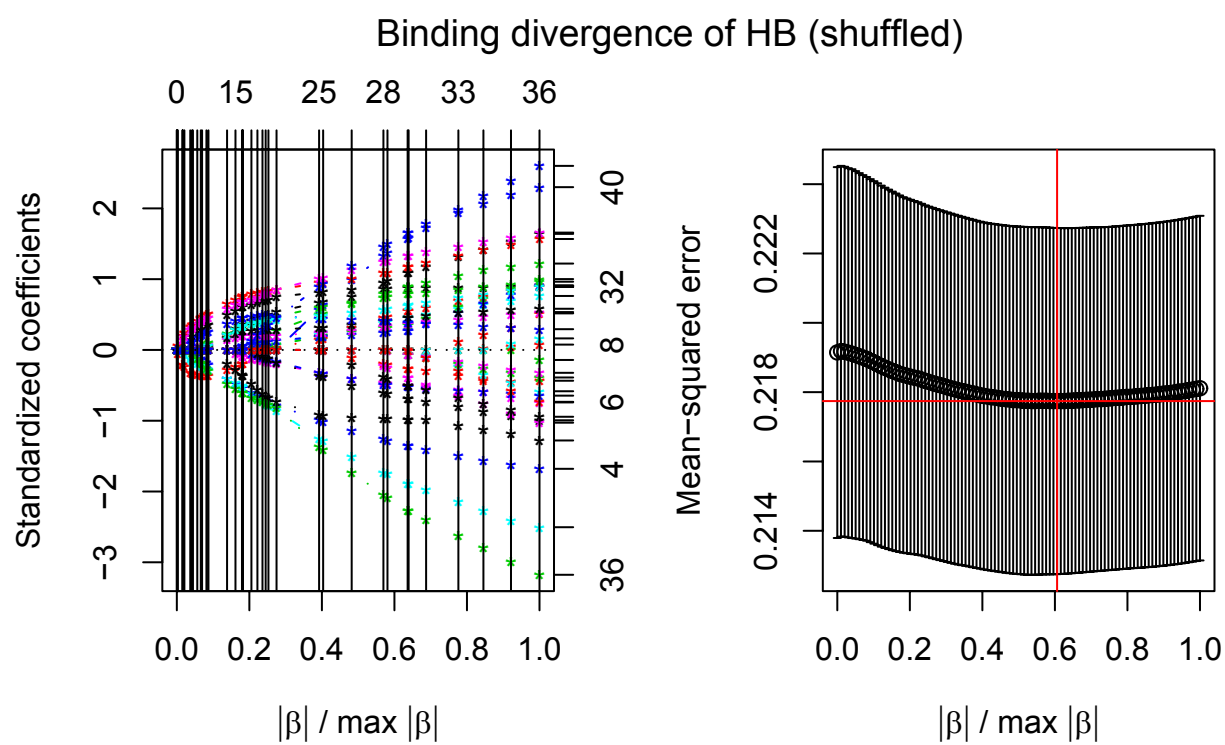

Supplement: Figure S22 — Linear model of HB binding divergence (control with shuffled words). Linear model of binding divergence using shuffled versions of the divergence-driving words (6 bp) identified for BCD. The increase in prediction error indicates that including more shuffled words in the linear model does not help with prediction, suggesting that the shuffled DDWs that we identified do not guide factor binding. Compare with . (0.10 MB PDF) [file pbio.1000343.s022.pdf]

### Binding divergence of HB (shuffled)

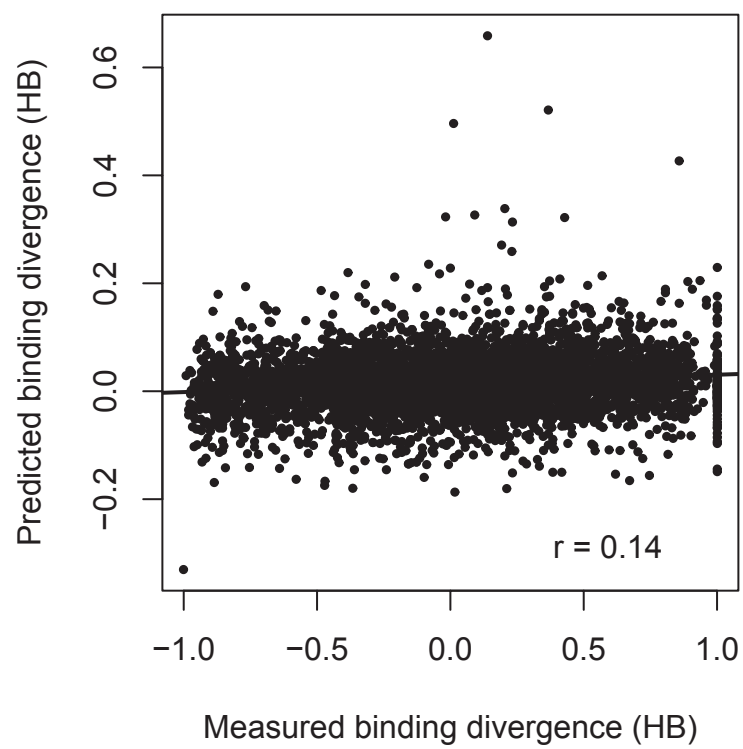

Supplement: Figure S23 — Accuracy of linear model predictions for HB (control with shuffled words). Scatterplot of measured and predicted binding divergence for BCD. Predictions used the linear model illustrated in Figure S22, where model coefficients were chosen to minimize the cross-validation prediction error. Compare with Figure S15. (0.30 MB PDF) [file pbio.1000343.s023.pdf]

Binding divergence of KR (shuffled)

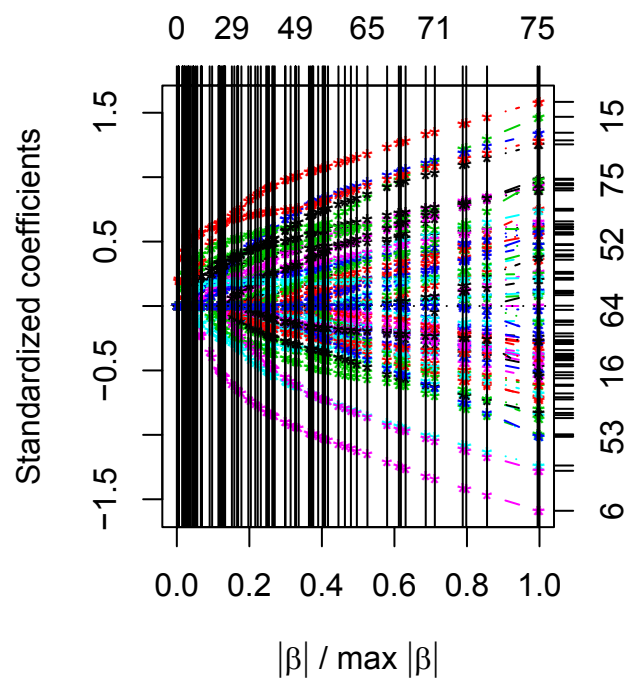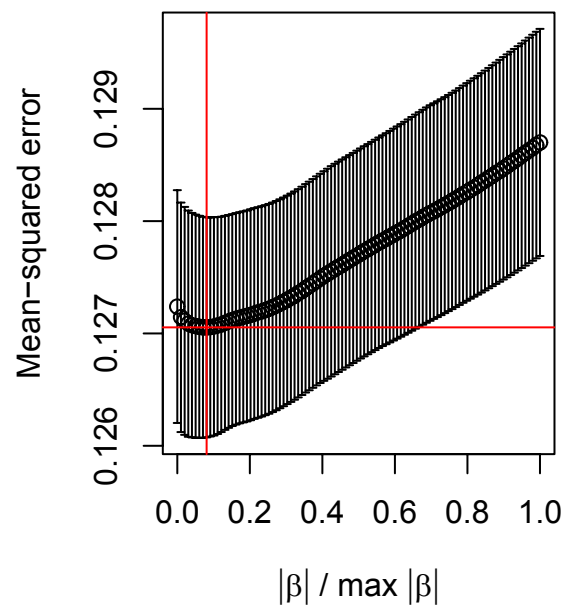

Supplement: Figure S24 — Linear model of KR binding divergence (control with shuffled words). Linear model of binding divergence using shuffled versions of the divergence-driving words (6 bp) identified for BCD. The increase in prediction error indicates that including more shuffled words in the linear model does not help with prediction, suggesting that the shuffled DDWs that we identified do not guide factor binding. Compare with . (0.36 MB PDF) [file pbio.1000343.s024.pdf]

Binding divergence of KR (shuffled)

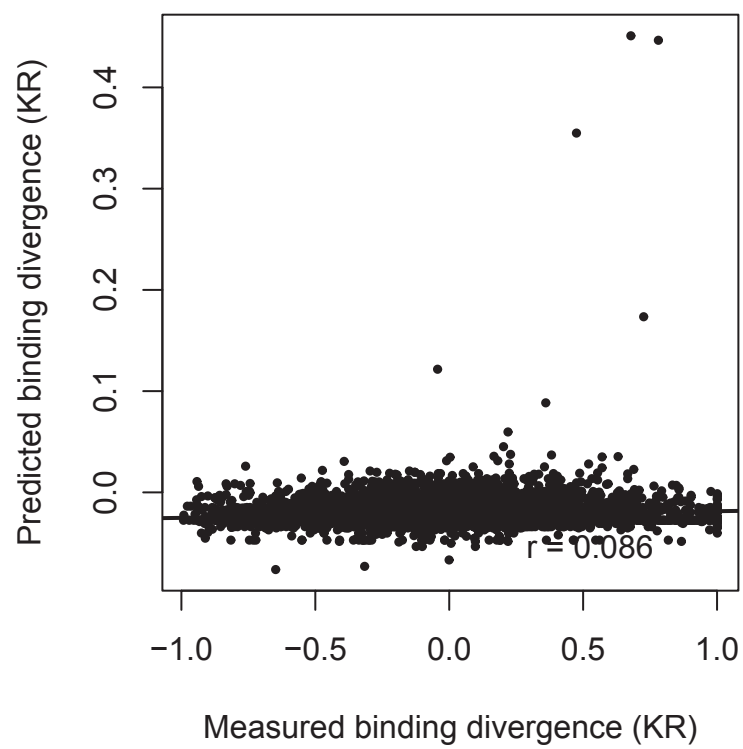

Supplement: Figure S25 — Accuracy of linear model predictions with KR (control with shuffled words). Scatterplot of measured and predicted binding divergence for BCD. Predictions used the linear model illustrated in Figure S24, where model coefficients were chosen to minimize the cross-validation prediction error. Compare with Figure S17. (0.43 MB PDF) [file pbio.1000343.s025.pdf]

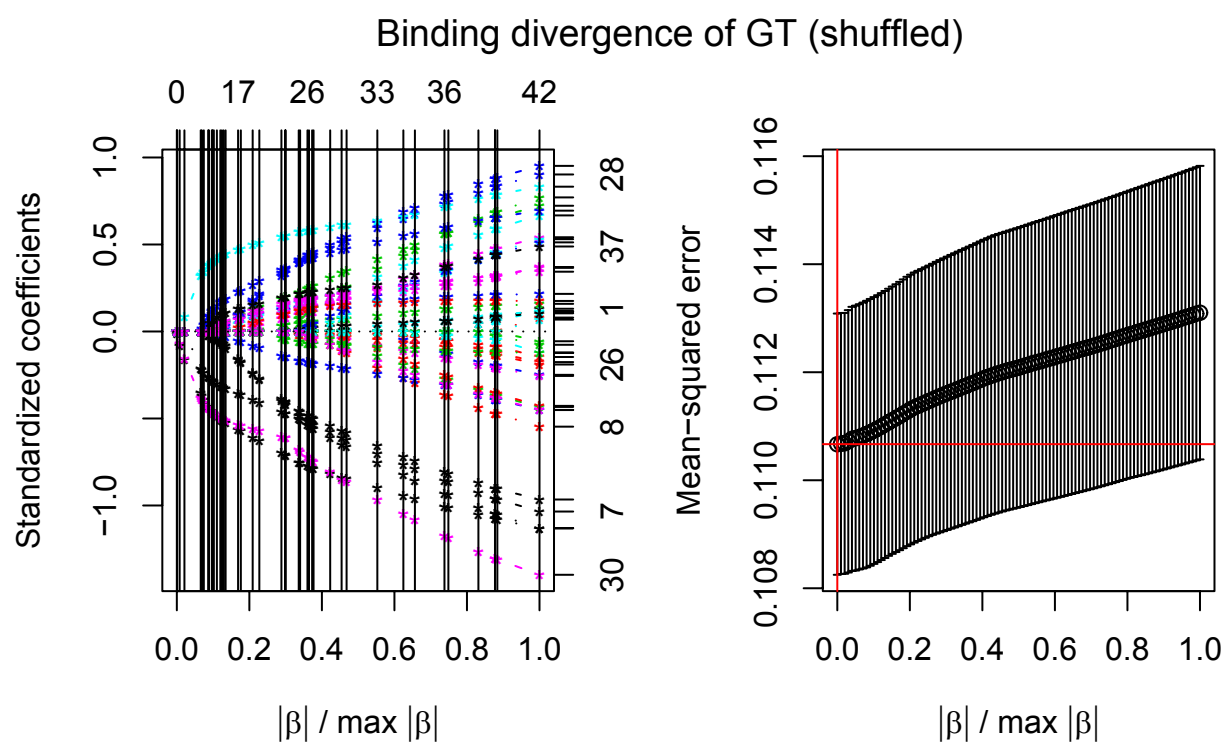

Supplement: Figure S26 — Linear model of GT binding divergence (control with shuffled words). Linear model of binding divergence using shuffled versions of the divergence-driving words (6 bp) identified for BCD. The increase in prediction error indicates that including more shuffled words in the linear model does not help with prediction, suggesting that the shuffled DDWs that we identified do not guide factor binding. Compare with Figure S18. (0.13 MB PDF) [file pbio.1000343.s026.pdf]

### Binding divergence of GT (shuffled)

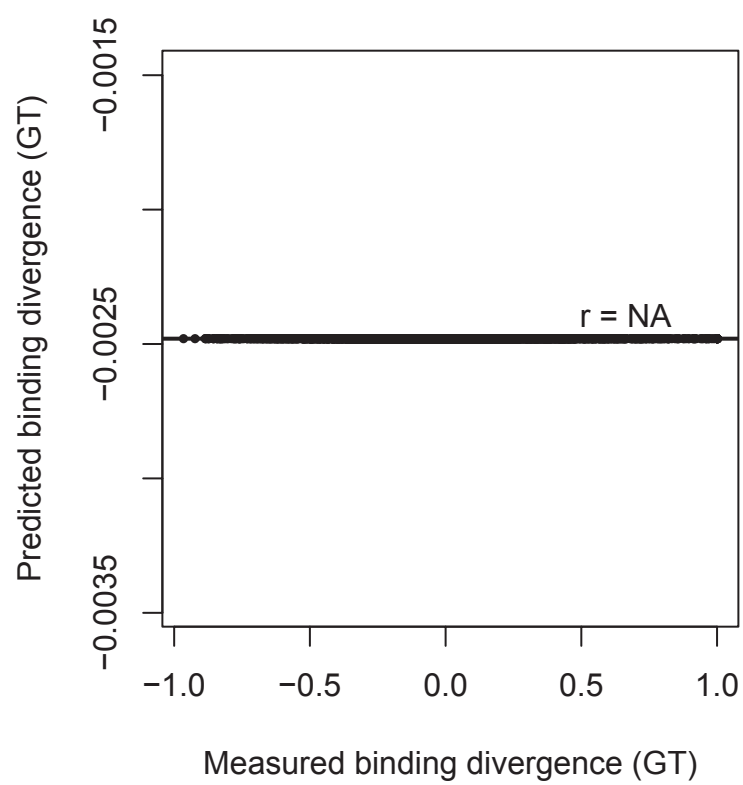

Supplement: Figure S27 — Accuracy of linear model predictions with GT (control with shuffled words). Scatterplot of measured and predicted binding divergence for BCD. Predictions used the linear model illustrated in Figure S26, where model coefficients were chosen to minimize the cross-validation prediction error. Compare with Figure S19. (0.18 MB PDF) [file pbio.1000343.s027.pdf]

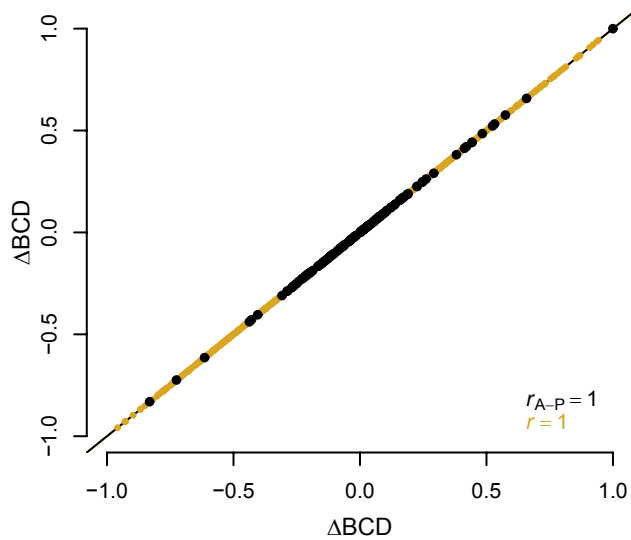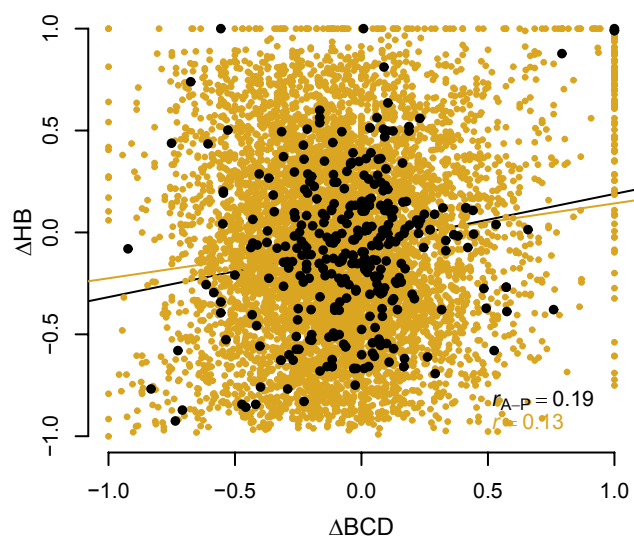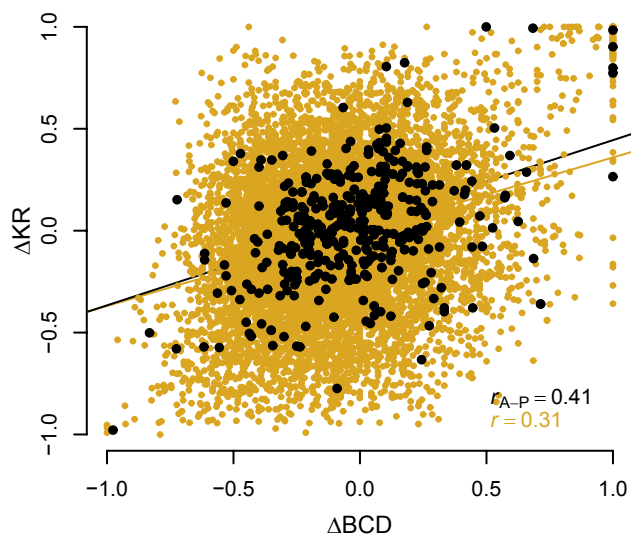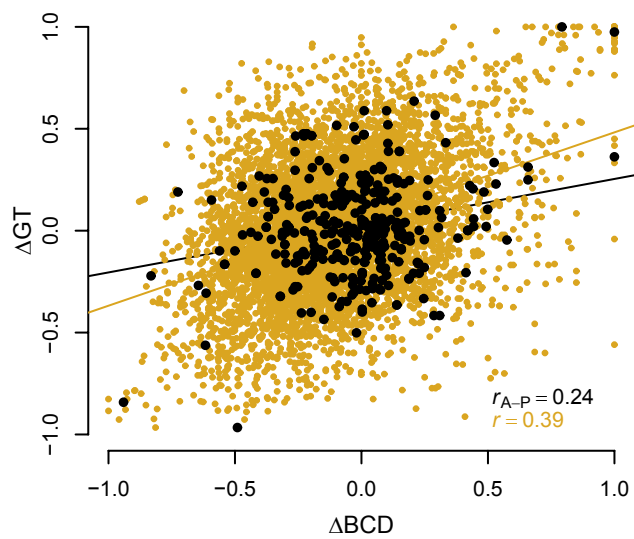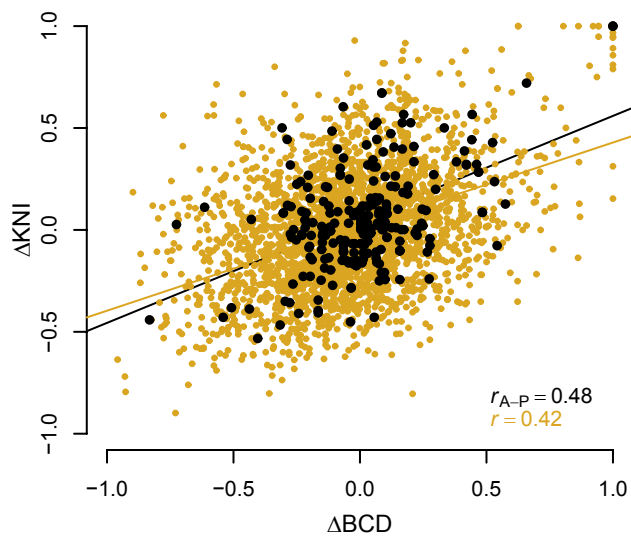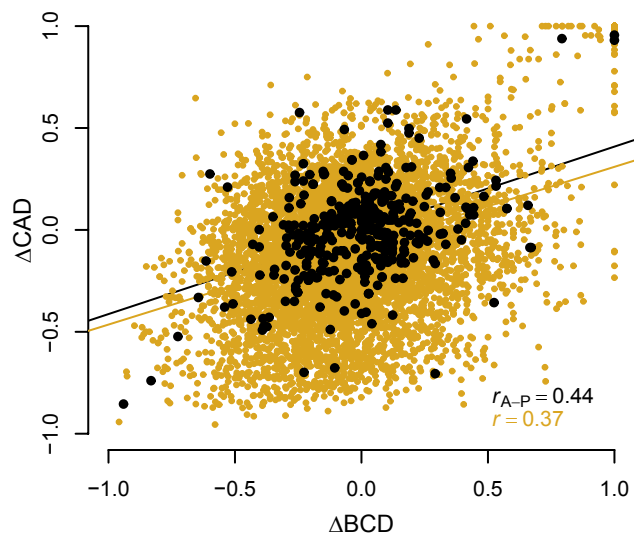

Supplement: Figure S28 — Correlations of fractional binding divergence of BCD with other factors. Correlations for fractional binding divergence, defined as (D. melanogaster − D. yakuba) / (D. melanogaster + D. yakuba). (2.34 MB PDF) [file pbio.1000343.s028.pdf]

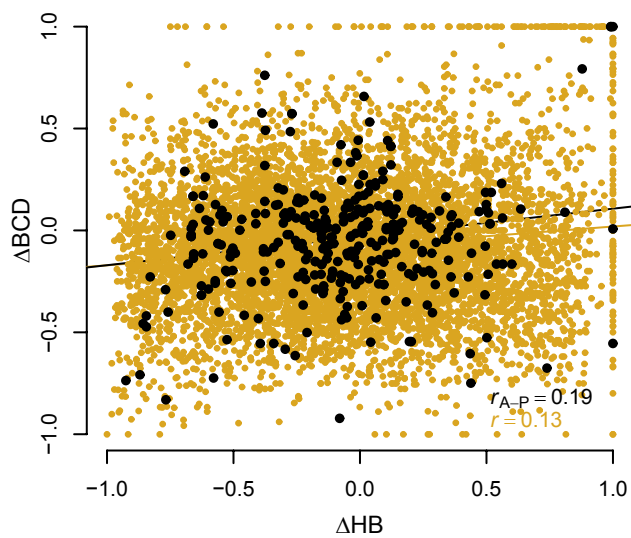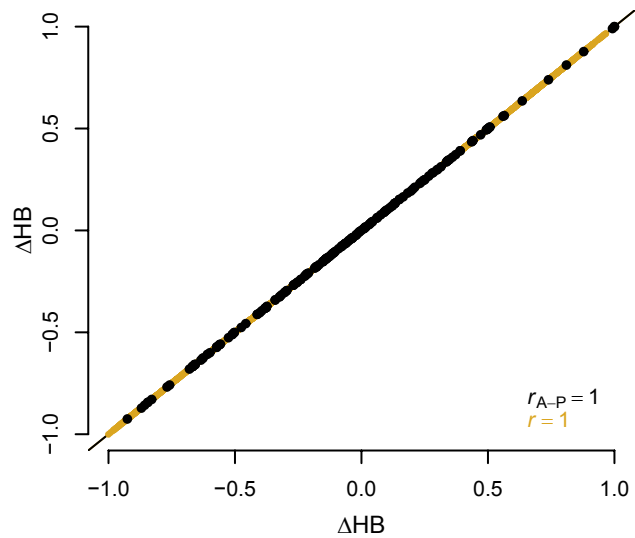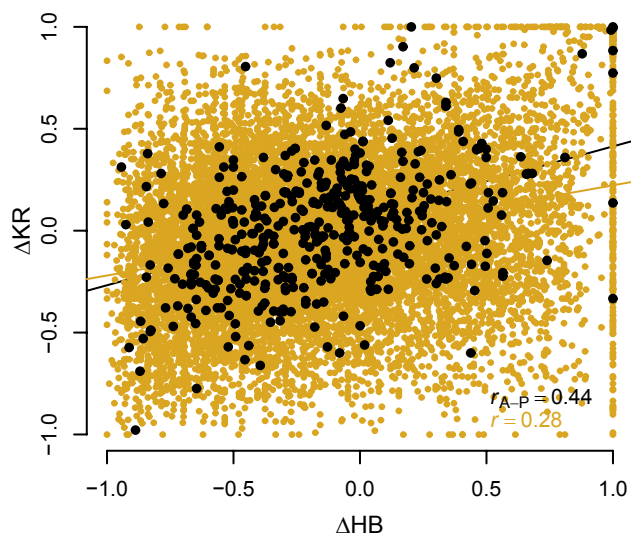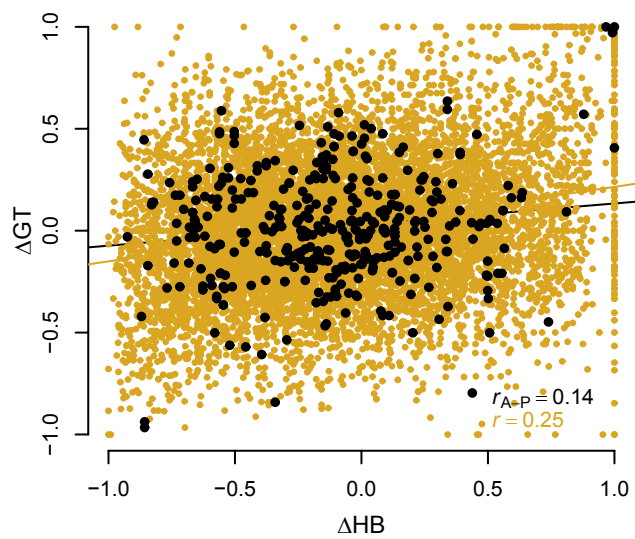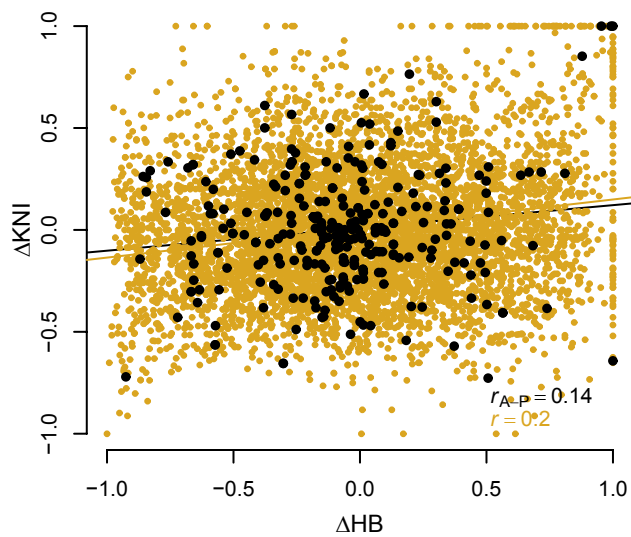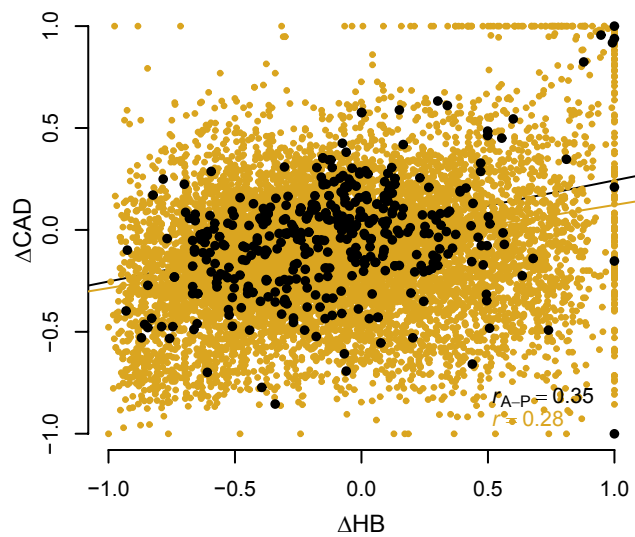

Supplement: Figure S29 — Correlations of fractional binding divergence of HB with other factors. Correlations for fractional binding divergence, defined as (D. melanogaster − D. yakuba) / (D. melanogaster + D. yakuba). (3.24 MB PDF) [file pbio.1000343.s029.pdf]

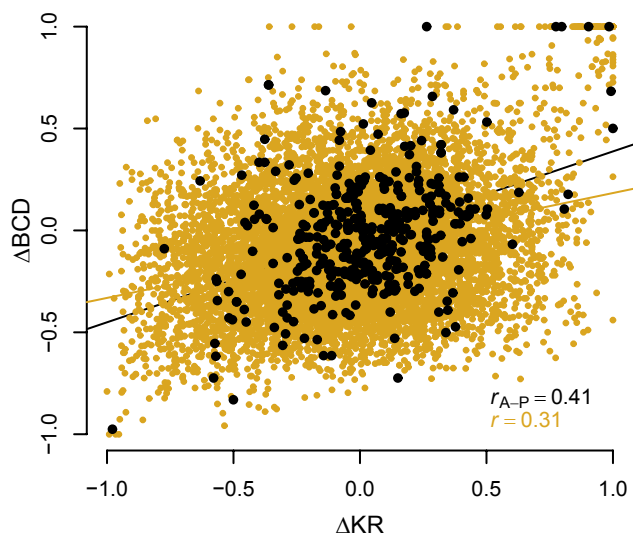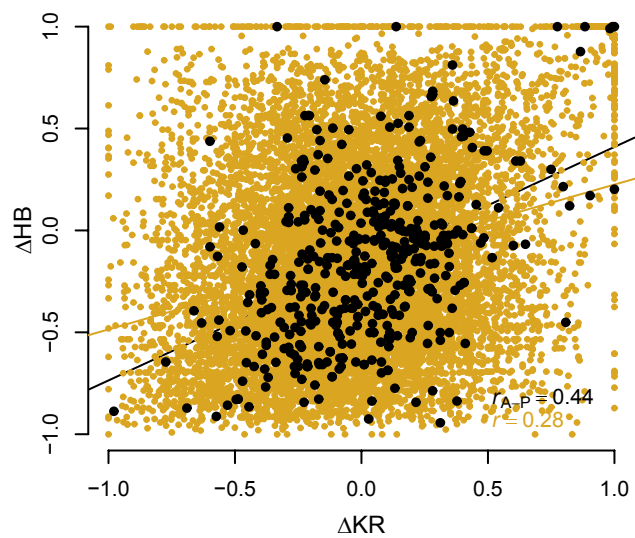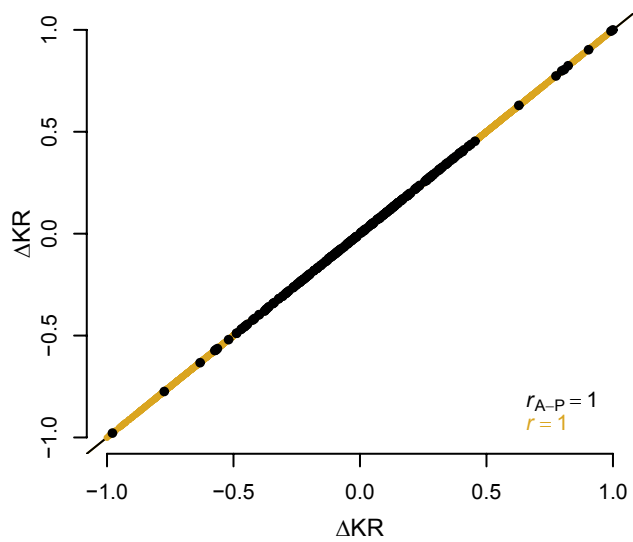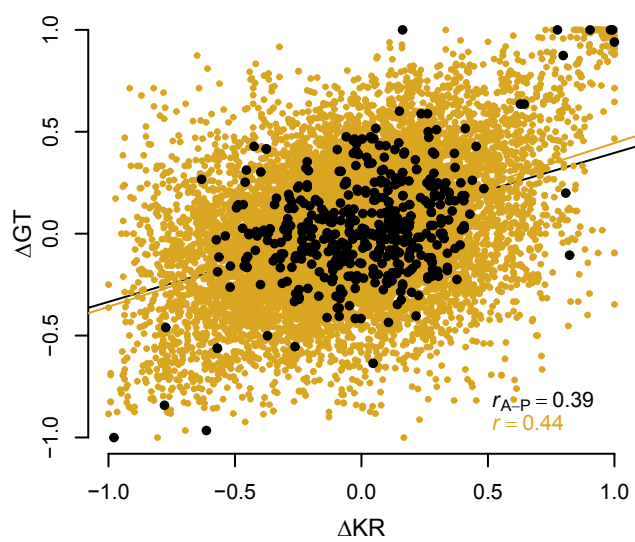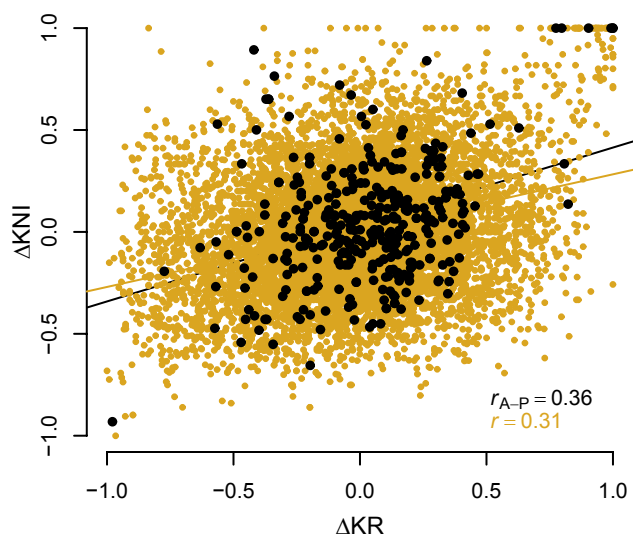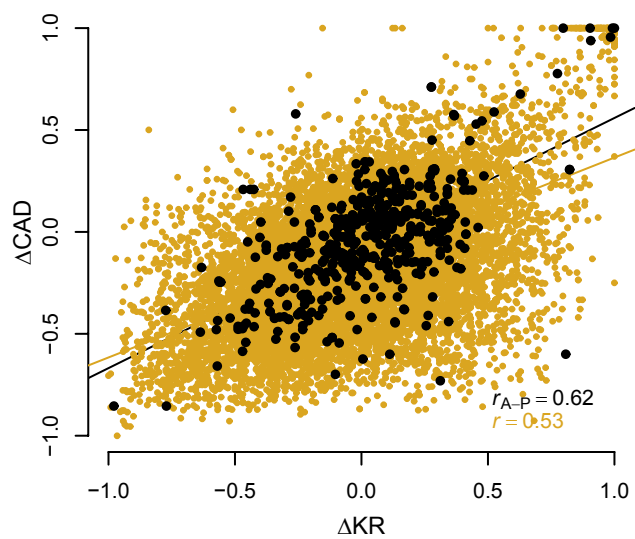

Supplement: Figure S30 — Correlations of fractional binding divergence of KR with other factors. Correlations for fractional binding divergence, defined as (D. melanogaster − D. yakuba) / (D. melanogaster + D. yakuba). (4.21 MB PDF) [file pbio.1000343.s030.pdf]

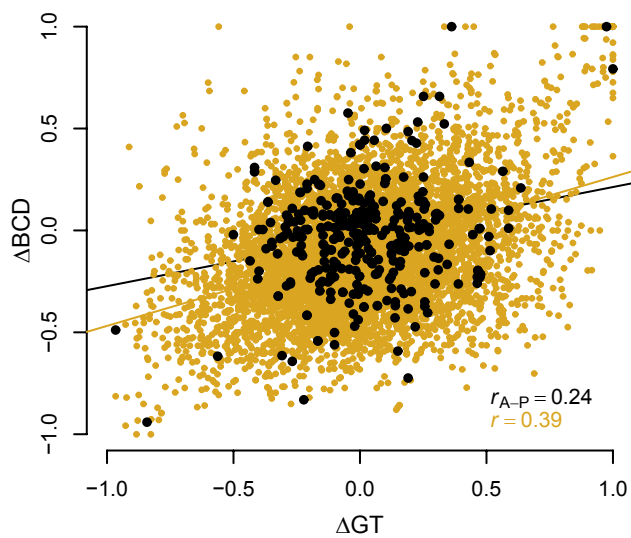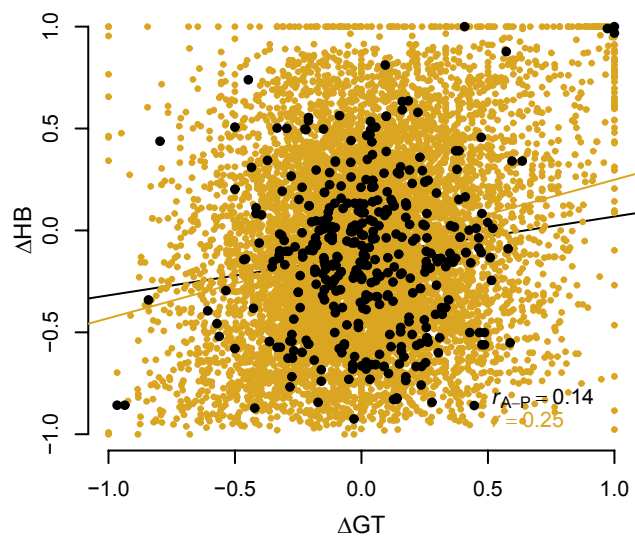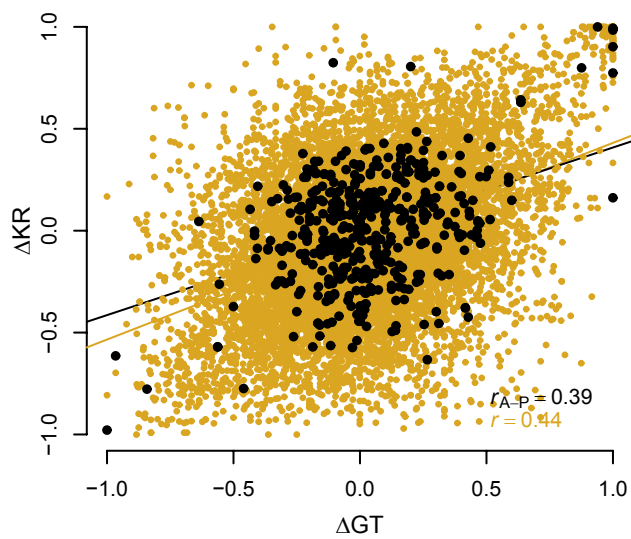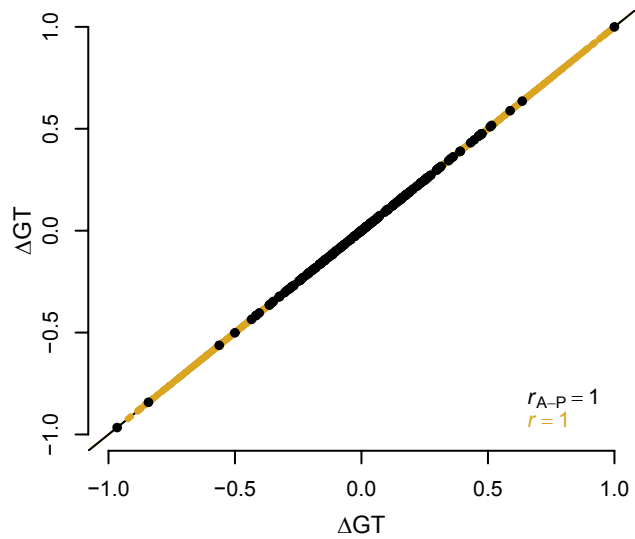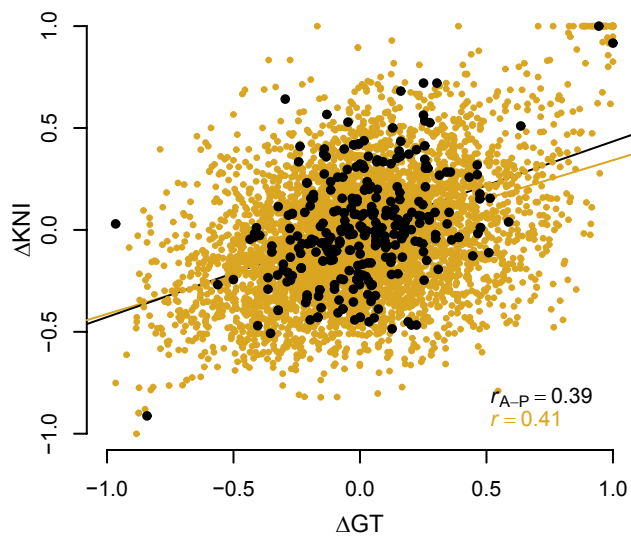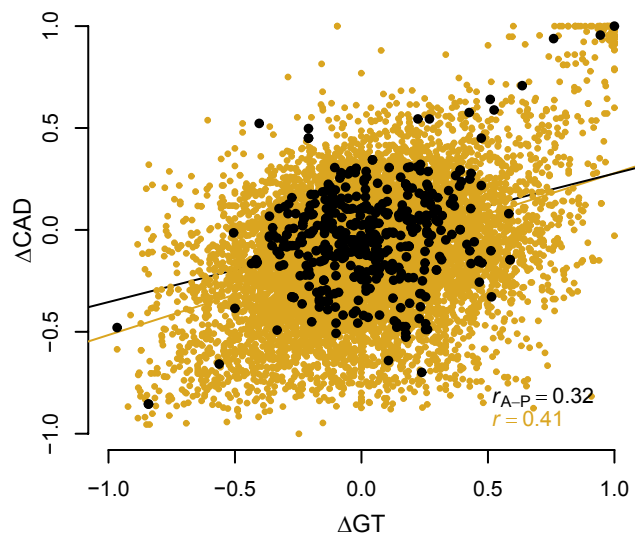

Supplement: Figure S31 — Correlations of fractional binding divergence of GT with other factors. Correlations for fractional binding divergence, defined as (D. melanogaster − D. yakuba) / (D. melanogaster + D. yakuba). (3.08 MB PDF) [file pbio.1000343.s031.pdf]

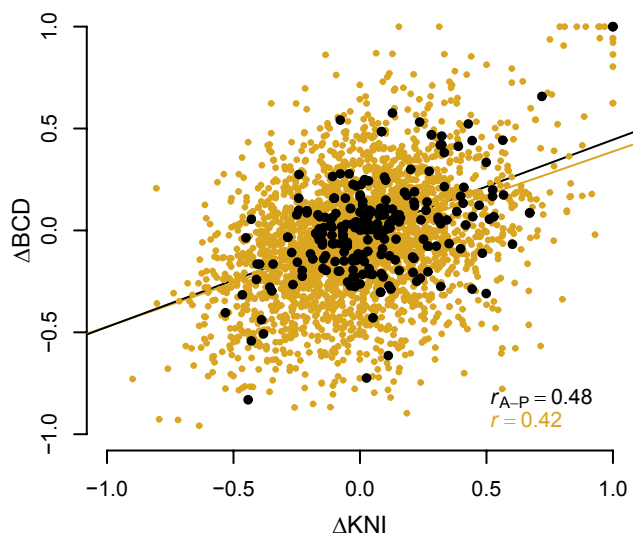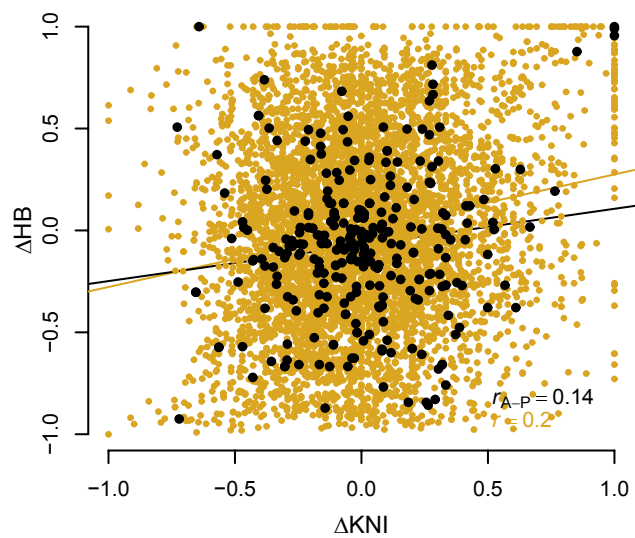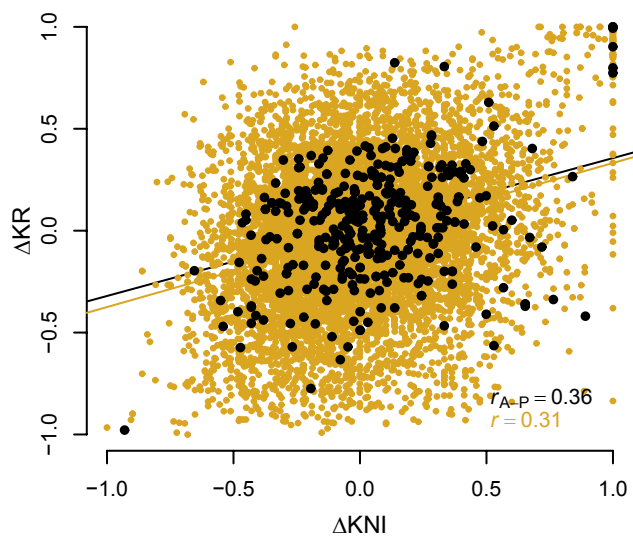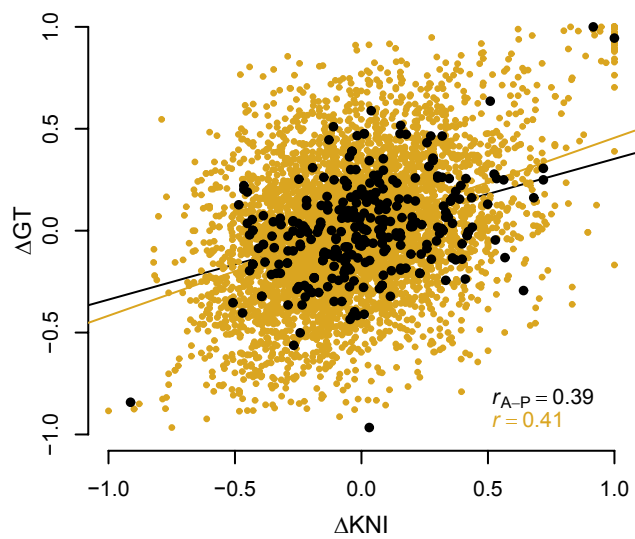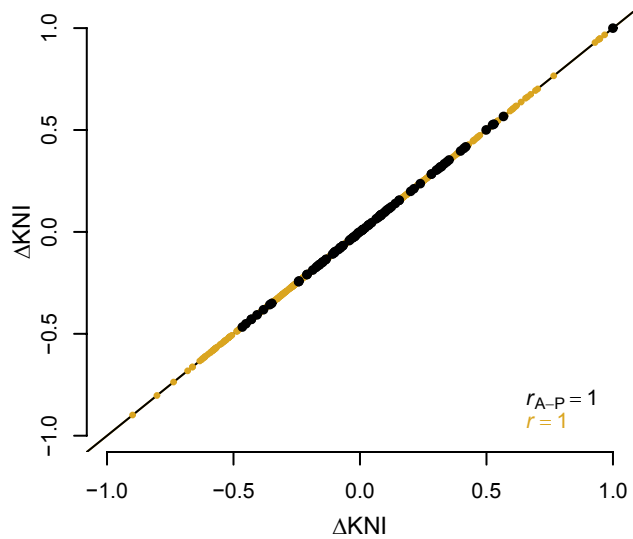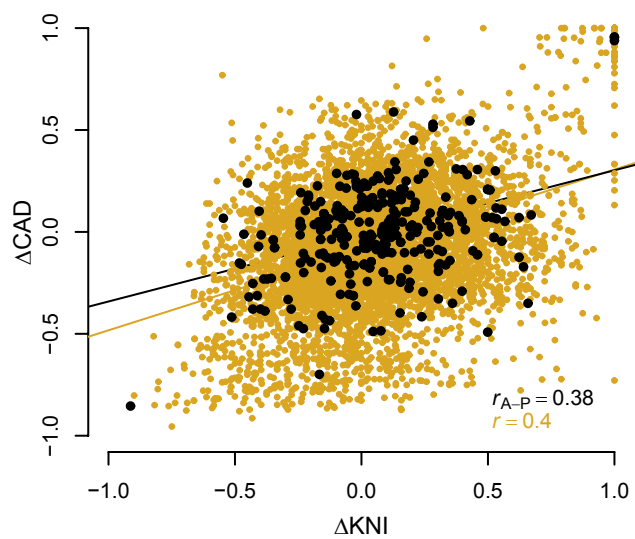

Supplement: Figure S32 — Correlations of fractional binding divergence of KNI with other factors. Correlations for fractional binding divergence, defined as (D. melanogaster − D. yakuba) / (D. melanogaster + D. yakuba). (1.89 MB PDF) [file pbio.1000343.s032.pdf]

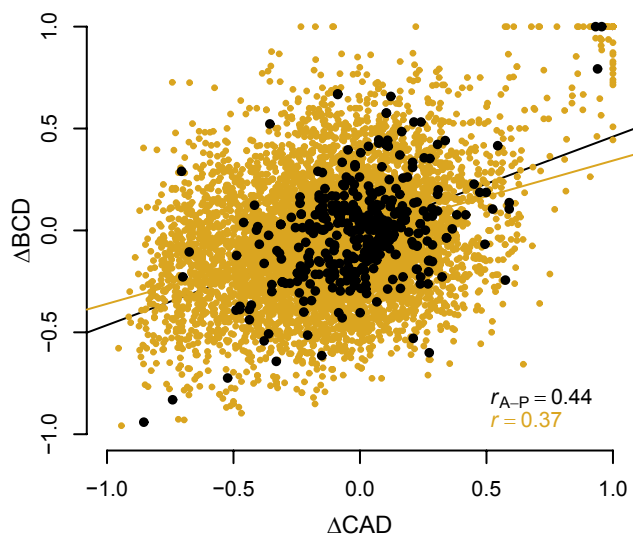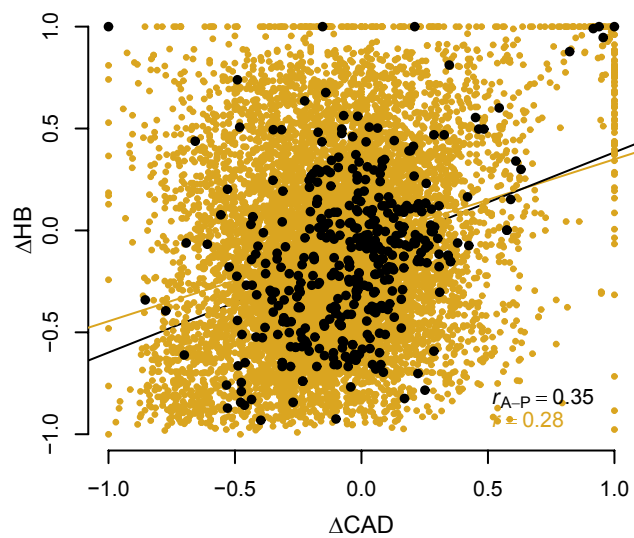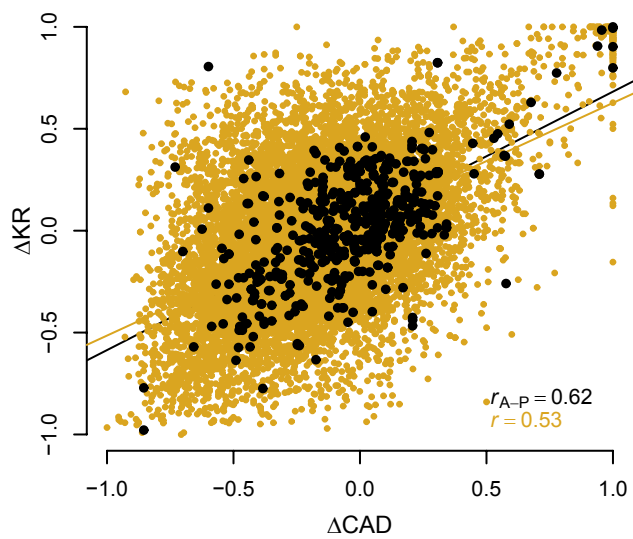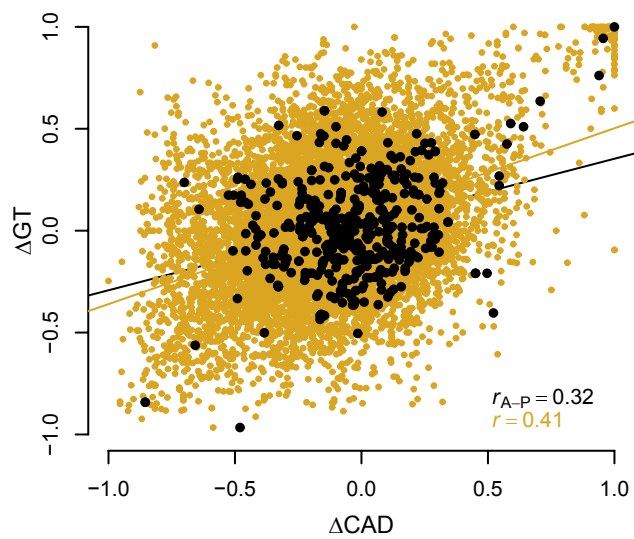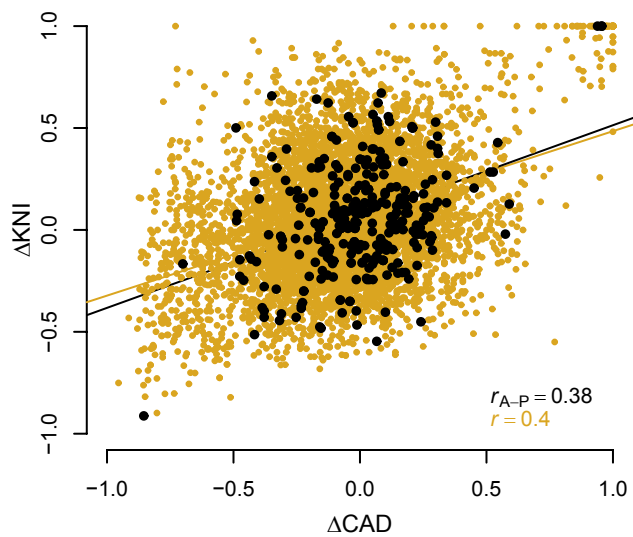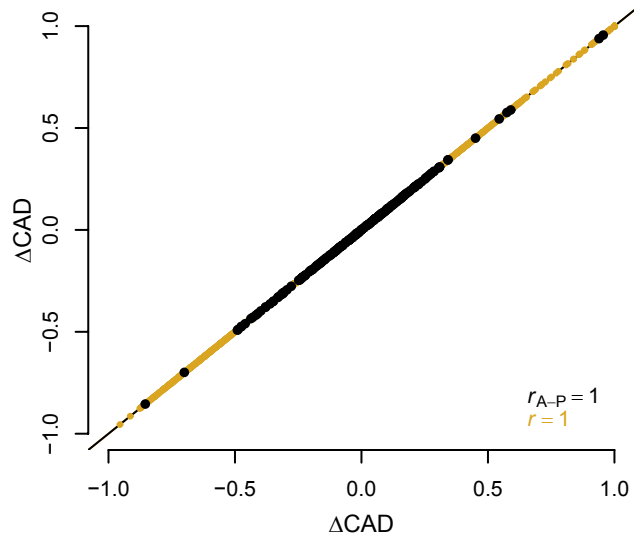

Supplement: Figure S33 — Correlations of fractional binding divergence of CAD with other factors. Correlations for fractional binding divergence, defined as (D. melanogaster − D. yakuba) / (D. melanogaster + D. yakuba). (3.30 MB PDF) [file pbio.1000343.s033.pdf]

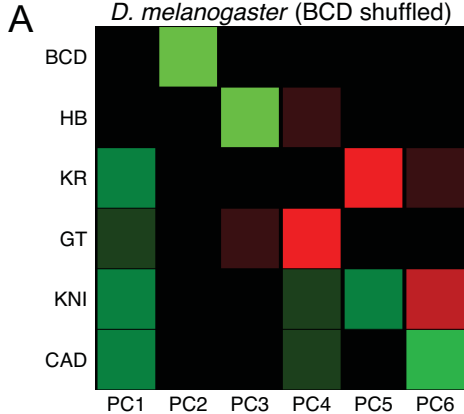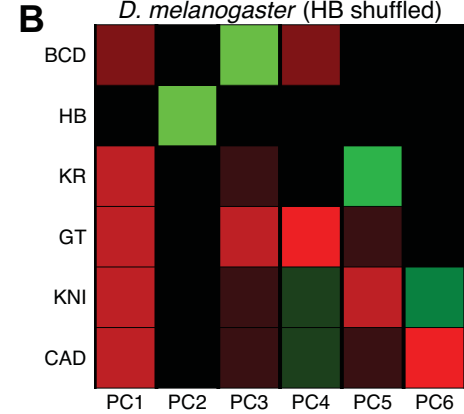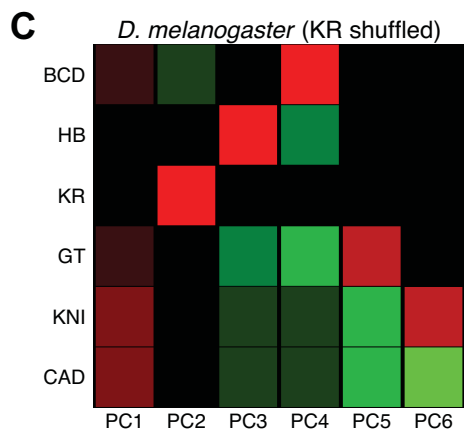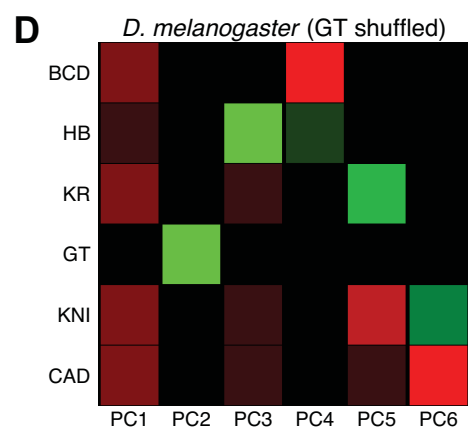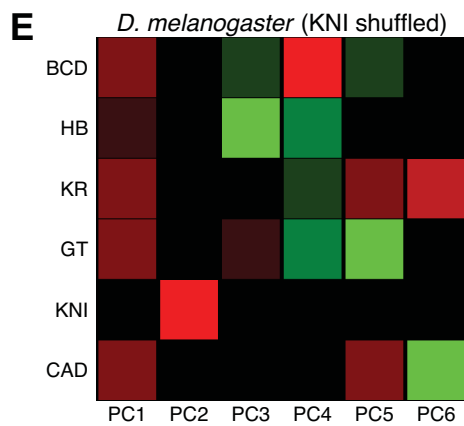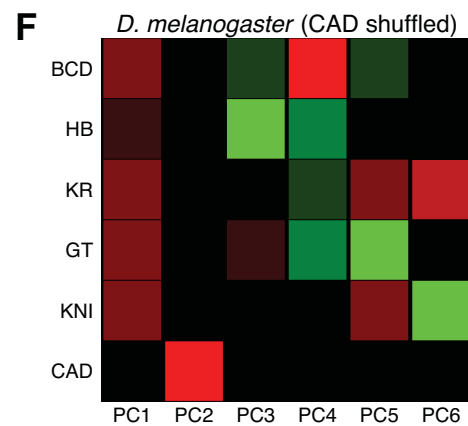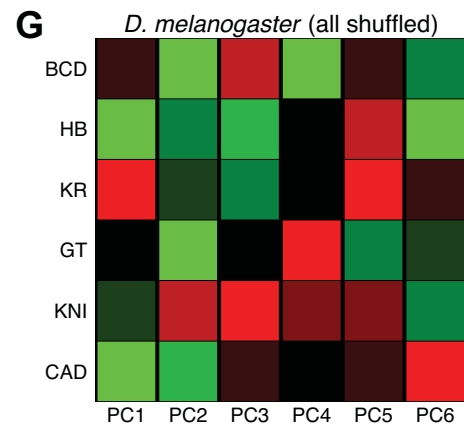

Supplement: Figure S34 — PCA controls. PCA after (A–F) randomly interchanging the measured binding strengths for single factors across called peaks while holding all others unchanged, and (G) similarly randomly interchanging the binding strengths of all factors. These operations remove spatial correlations between the binding of single factors and the other five (A–F) and spatial correlations between the binding of any factors (G). As expected, the chromatin signal disappeared after performing any of these transformations on the data. (0.43 MB PDF) [file pbio.1000343.s034.pdf]
